# Supplementary material for: Researching COVID to enhance recovery (RECOVER) tissue pathology study protocol: Rationale, objectives, and design
Source: PLoS One. 2024 Jan 10;19(1):e0285645. doi: 10.1371/journal.pone.0285645 (PMC10781091; doi:10.1371/journal.pone.0285645)
Supplement: S1 Protocol — (PDF) [file pone.0285645.s001.pdf]

S1 Protocol. NIH RECOVER Tissue Pathology CNS MRI Protocol

Authors: Natalie Adolphi and Alan Seifert

The ex vivo MRI protocol is intended to be feasible to harmonize across multiple autopsy cohort sites without relying on advanced research-specific hardware or software. For this reason, the protocol was developed for a clinical-grade 3 Tesla MRI scanner (Skyra, Siemens Healthineers, Erlangen, Germany), a standard 20-channel head and neck radiofrequency coil, and standard vendor-supplied pulse sequences. The entire protocol is limited to 60 minutes for logistical feasibility across all participating sites.

For cases where ex vivo MRI is planned, the right hemisphere is harvested at autopsy and fixed by immersion in 10% neutral buffered formalin for a minimum of 4 weeks at 4°C. In preparation for imaging, the brain is agitated under vacuum to dislodge air bubbles from sulci and ventricles. The hemibrain is then packaged securely into a custom-designed imaging vessel, which is filled with a ^{1}H-free susceptibility matching liquid (Fluorinert or Fomblin), exposed to vacuum again to dislodge any remaining air bubbles, and then sealed, placed into a secondary containment bag, and brought to the scanner.

The MRI protocol consists of localizer images and multiple iterations of shimming by 3D mapping, followed by 3D fluid-attenuated inversion recovery (FLAIR), 3D T2-weighted turbo-spin echo (TSE) with optimized flip angle schedules for isotropic imaging, 3D multi-echo gradient-echo (GRE), 3D susceptibility-weighted imaging (SWI), and multi-slice 2D multi-shell diffusion-weighted imaging (b = 0 plus 64 directions at each of b = 1000, 2000, 5000 s/mm^2, with additional phase-reversed b = 0 images). Images are sent to a centralized PACS system for subsequent interpretation and quantitative analysis.
