# Supplementary material for: Researching COVID to enhance recovery (RECOVER) tissue pathology study protocol: Rationale, objectives, and design
Source: PLoS One. 2024 Jan 10;19(1):e0285645. doi: 10.1371/journal.pone.0285645 (PMC10781091; doi:10.1371/journal.pone.0285645)
Supplement: S2 Protocol — (PDF) [file pone.0285645.s002.pdf]

STUDY TITLE: NIH RECOVER: A Multi-site Pathology Study of Post-Acute Sequelae of SARS-CoV-2 Infection

SHORT TITLE: NIH RECOVER Tissue Pathology:

Understanding the Long-Term Impact of COVID

DATE 7 FEB 2022

Date: 7 FEB 2022

Protocol Revision History:

Version Number	Status/Summary of Revisions Made	Version Date
1.0	Autopsy protocol submitted to NIH	09 JUL 2021
1.1	Title correction	15 SEP 2021
1.2	Clarification of site responsibilities	10 NOV 2021
1.3	Additional detail regarding statistical considerations	07 DEC 2021
1.4	General editing for clarity	09 JAN 2022
2.0	Autopsy protocol submitted to OSMB for final approval	07 FEB 2022
2.1	Added CNS Brain MRI protocol to Study Procedures	12 JUL 2022
3.0	Expanded Protocol Acute timing definition changes	25 APR 2023

Date: 7 FEB 2022

**Principal Investigators**<br>**Clinical Science Core**	**Stuart Katz, MD MS**<br>NYU Grossman School of Medicine<br>Leon H. Charney Division of Cardiology<br>530 First Avenue, Skirball 9R<br>New York, NY 10016<br><br>**Leora Horwitz, MD**<br>NYU Grossman School of Medicine<br>Department of Population Health<br>Division of Healthcare Delivery Science<br>227 East 30th Street<br>New York, NY 10016<br><br>**Andrea B. Troxel, ScD**<br>NYU Grossman School of Medicine<br>Department of Population Health<br>Division of Biostatistics<br>180 Madison Avenue, Suite 5-55<br>New York, NY 10016
**Contact Principal Investigator**<br>**Data Resource Core**	**Andrea Foulkes, ScD**<br>Mass General Hospital<br>Harvard Medical School Department of Medicine<br>50 Staniford Street, Suite 560<br>Boston, MA 02114<br>afoulkes@mgh.harvard.edu
**Protocol Biostatistician**	**Marie-Abele Bind, PhD**<br>Mass General Hospital<br>Harvard Medical School Department of Medicine<br>50 Staniford Street, Suite 560<br>Boston, MA 02114<br>mbind@mgh.harvard.edu
**Bioethicist**	Brendan Parent, JD<br>NYU Grossman School of Medicine<br>Dept. Population Health<br>Division of Medical Ethics<br>Brendan.Parent@nyulangone.org
**NYU sIRB study number**	Not applicable

Statement of Compliance

According to the Department of Health and Human Services Office for Human Research Protections' Code of Federal Regulations 45 CFR 26, human subjects are "living individuals."(1) Study of autopsy materials, therefore, is deemed to be not human subjects research and therefore is not subject to Institutional Review Board (IRB) governance for review. All autopsies will be conducted with consent from next of kin and in accord with all institutional, local government, and Federal government laws, statutes, and regulations. Development and

implementation of educational materials to increase awareness of the scientific importance of body donation and research with the recently dead will occur in consultation with a patient advisory panel and review by NIH Office of Human Subjects Research.

This proposed research study of standardized autopsy procedures in the recently dead and transfer of collected decedent tissues to a centralized biorepository will be conducted in accordance with established ethics guidelines.(2) These guidelines incorporate the principles of review and oversight, community involvement, eligibility, research procedures, consent, confidentiality, and resource use and cost. This protocol will undergo review by the RECOVER OSMB in order to ensure harmonization of the protocol procedures with the distinct ethical issues involved in research with the recently dead.

The Principal Investigator will assure that no deviation from, or changes to, the protocol will take place without prior agreement from the sponsor.

The signature below provides the necessary assurance that this study will be conducted according to all stipulations of the protocol including confidentiality statements, and according to local legal and regulatory requirements, and US federal regulations (if applicable).

Version Date: 7 FEB 2021

___________________________________ __________________________

Signature of Site Principal Investigator Date

____

Printed Name of Site Principal Investigator

____

Name of Facility

____

Location of Facility (City, Country)

Date: 7 FEB 2022

TABLE OF CONTENTS

1 PASC PROTOCOL SUMMARY 8

2 INTRODUCTION, BACKGROUND INFORMATION, AND SCIENTIFIC RATIONALE 14

2.1 INTRODUCTION AND BACKGROUND INFORMATION 14
2.2 SCIENTIFIC RATIONALE 15

3 STUDY OBJECTIVES 16

4 SPECIFIC AIMS 16

5 STUDY DESIGN 17

5.1 OVERALL STUDY DESIGN 17
5.2 STUDY RECRUITMENT 18
5.3 ELIGIBILITY CRITERIA 19
5.4 WORKING DEFINITION OF PASC 20
5.5 GENERALIZABILITY 21
5.6 SAMPLE SIZE AND POWER CALCULATIONS 21

6 STUDY METHODS 23

6.1 DATA SOURCES AND PRIORITIZATION 23
6.2 METHODS OF DATA COLLECTION 23
6.3 RETURN OF RESULTS TO FAMILIES 24
6.4 STRATEGIES FOR STUDY MODIFICATIONS 24
6.5 OVERVIEW OF ANALYTIC APPROACH TO AIMS 24
6.6 DATA ENTRY AND MANAGEMENT 26

7 ETHICAL CONSIDERATIONS 30

7.1 RESEARCH INVOLVING THE DEAD 30
7.2 INFORMED CONSENT PROCESS 30
7.3 POTENTIAL RISKS 30
7.4 CONFIDENTIALITY, PROTECTION AGAINST RISKS 31
7.4.1 Storage of Study Materials 31
7.4.2 Hashed identifiers 31
7.5 CERTIFICATE OF CONFIDENTIALITY 31
7.6 POTENTIAL BENEFITS 32
7.7 RISK/BENEFIT RATIO AND IMPORTANCE OF INFORMATION TO BE OBTAINED 32

7.8 WOMEN AND MINORITY INCLUSION IN CLINICAL RESEARCH 33
7.9 DATA SAFETY MONITORING PLAN 33

8 STUDY POLICIES 34

8.1 CONFLICT OF INTEREST 34
8.2 PUBLICATIONS 35
8.3 MONITORING BOARD 35

9 RESOURCE SHARING PLAN 36

10 APPENDICES 38

10.1 GROSS AUTOPSY TEMPLATE 38

11 REFERENCES 45

LIST OF TABLES

Table 1 provides detectable effect sizes and detectable differences in proportions between PASC+ and PASC- individuals for quantitative and dichotomous pathological features. Results are given with and without a multiple comparison adjustment (assuming 50 tests, alpha=0.001 conservative control). 21

LIST OF FIGURES

Figure 1: Recruitment Source for RECOVERY Autopsy 18
Figure 2 Architecture of Data Flow from Study Sites to the DCC and DRC 29

LIST OF ABBREVIATIONS

AE	Adverse Event/Adverse Experience
BMI	Body mass index
CAP	College of American Pathologists
CFR	Code of Federal Regulations
COVID-19	Coronavirus Disease 2019
CRF	Case Report Form
DHHS	Department of Health and Human Services
DSMB	Data and Safety Monitoring Board
EHD	Electronic Health Database
EHR	Electronic Health Record
FAD	Final Anatomic Diagnosis
GCP	Good Clinical Practice
HIPAA	Health Insurance Portability and Accountability Act of 1996
ICF	Informed Consent Form
ICH	International Conference on Harmonisation
ICMJE	International Committee of Medical Journal Editors
IRB	Institutional Review Board
N	Number (typically refers to participants)
NIH	National Institutes of Health
OHRP	Office for Human Research Protections
OHSR	Office of Human Subjects Research
OSMB	Observational Study Monitoring Board
PASC	Post-Acute Sequelae of COVID-19
PI	Principal Investigator
REDCap	Research Electronic Data Capture
SOP	Standard Operating Procedure
US	United States
WHO	World Health Organization

1 PASC PROTOCOL SUMMARY

The Post-Acute Sequelae of SARS-CoV-2 (PASC) Autopsy Study is a cross-sectional study designed to define and characterize the epidemiology, natural history, clinical spectrum, and underlying mechanisms of post-acute effects of SARS-CoV-2 infection in a diverse population representative of the general COVID-19 population in the US. The autopsy study will characterize the pathology of PASC in (i) non-hospitalized patients who die 60 days or later from symptom onset of COVID-19, . The study will include decedents who had previously fully recovered from SARS-CoV-2 infection (i.e., >60 days from onset in non-hospitalized), and decedents who meet clinical criteria of PASC as defined by the recent World Health Organization publication (see Section 5.4 below). The autopsy study will also explore the pathology of acute SARS-CoV-2 infection in a smaller subset of patients who died 15-60 days from symptom onset. This protocol defines the common set of clinical data elements, autopsy procedures for tissue collection, core measures, pathology protocols, shared pathology tissues, data elements, and methodology. Each investigator site is expected to perform autopsies on the decedents to address the pathophysiology of the potential long-term effects of SARS-CoV-2 infection on human health. The Consortium analysis plan aims to address research questions by incorporating: 1) tissue obtained from autopsies performed at each Phase II participant's site; and 2) tissue available from other pathology investigators/autopsy sites within the Consortium.

RECOVER cohort participants from centers across the United States will receive educational material to increase awareness of the scientific importance of body donation and research of the recently dead in the RECOVER initiative. Identified decedents from the RECOVER cohorts and other referral sources will be enrolled in a stratified approach to ensure adequate diversity of race and ethnicity. Cohort participants may be ascertained before confirmed SARS-CoV-2 infection, at the time of acute infection, or after acute infection. Gross and microscopic findings at autopsy will be analyzed to evaluate findings associated with pre-COVID medical conditions as well as findings that may be associated with PASC, and to evaluate possible correlations with risk factors. Biospecimens will be collected from all participants and

stored in the RECOVER biorepository for future analysis. Advanced post-mortem imaging (brain MRI) will be conducted in a subset of decedents.

The study is funded for 4 years by the NIH.

Protocol Synopsis

**Title**	NIH RECOVER: A Multi-Site Pathology Study of Post-Acute Sequelae of SARS-CoV-2 Infection
**Short Title**	NIH RECOVER Tissue Pathology: Understanding the Long-Term Impact of COVID
**Primary Objectives**	**Aim 1.** Characterize the prevalence and types of organ injury/disease, pathological features, and distinct phenotypes in decedents with acute SARS-CoV-2 infection (dying 15-60 days after infection), and in decedents with prior SARS-CoV-2 infection (dying >60 days after infection) with and without PASC.<br><br>**Aim 2.** Characterize the association of pathological findings with clinical measures of disease severity and associated risk factors in decedents with prior SARS-CoV-2 infection who die more than 60 days after initial onset or after hospital discharge, whether in-hospital or out-of-hospital at time of death, with and without PASC.<br><br>*2a.* Characterize the association of COVID-19 disease severity and treatment with pathological findings in decedents with prior SARS-CoV-2 infection with and without PASC .<br><br>*2b*. Determine whether pre-infection and peri-infection risk and resiliency factors (e.g., demographic, biological factors, and preexisting clinical comorbidities) are associated with pathological findings in decedents with prior SARS-CoV-2 infection with and without PASC.<br><br>**Aim 3.** Define the pathophysiology of and mechanisms associated with pathological findings, including the direct and indirect causal effects of clinical risk factors (e.g., comorbidities, infection severity, hospitalization, intubation, steroid treatment) on pathological findings (e.g., inflammation, fibrosis, necrosis) via viral persistence (investigated systematically via molecular methods), in decedents with and without PASC.<br><br>**Aim 4.** Establish a multi-center post-mortem tissue biobank and post-mortem brain imaging bank from decedents with prior SARS-CoV-2 infection with and without PASC. The primary goal of the brain imaging is to find and characterize lesions in the CNS via image-directed sampling of identified lesions. A secondary goal is to correlate decedent's MRI findings with those observed in pre-mortem neuroimaging.

**Study Design**	Cross-sectional study. Decedents may be enrolled from extant NIH funded cohort studies; clinical cohorts of those with acute infection or PASC; or from new clinic and community recruitment (acute infection, post-infection, or PASC). Autopsies of all of these decedents will follow a standardized protocol.
**Observational Mode**	Cross-sectional
**Time Perspective**	Prospective
**Endpoints**	Primary Endpoint: Presence of pathological findings indicative of candidate PASC symptoms.<br><br>Secondary Endpoints: Biological effects of SARS-CoV-2 infection; organ injury; other clinical disease.
**Study Duration**	Four years.
**Participant Duration**	Not applicable.
**Population**	PASC Cases: Individuals meeting WHO criteria for suspected, probable, or confirmed SARS-CoV-2 infection who die more than 60 days after initial onset or after hospital discharge, whether in-hospital or out-of-hospital at time of death, and who meet the working definition of PASC given in Section 5.4.<br><br>Non PASC Individuals:<br>1. Acute infected: Individuals meeting WHO criteria for suspected, probable, or confirmed SARS-CoV-2 infection who die 15-60 days after initial onset or 15-60 days after discharge, whether in-hospital or out-of-hospital at time of death;<br>2. Infected without PASC: Individuals meeting WHO criteria for suspected, probable, or confirmed SARS-CoV-2 infection who die without symptoms of PASC more than 60 days after initial onset.<br><br>Next of kin will provide consent in accordance with local legal requirements to participate in autopsy and biological sample collection as specified in the protocol procedures.
**Study Sites**	Phase 2 PASC Consortium Autopsy Cohort Sites
**Number of decedents**	Approximately 700 total decedents, including 400 with prior SARS-CoV-2 infection (dying > 60 days after SARS-CoV-2 infection) with PASC, 100 with acute SARS-CoV-2 infection (dying 15-60 days from infection), and 200 with prior SARS-CoV-2 infection (dying > 60 days after SARS-CoV-2 infection) without PASC.
**Key Procedures**	Investigators will use common data elements and models set forth by the FDA for the study of COVID (https://www.fda.gov/drugs/coronavirus-covid-19-drugs/covid-19-real-world-data-rwd-data-elements-harmonization-project)

	and adapt and expand these as needed to specific projects and as determined by the NIH for PASC via this mechanism.<br><br>**Autopsy**. Autopsies must be performed within 24 hours of death. Procedures must comply with those set forth in Appendix 1: Gross Autopsy Template and the Manual of Procedures.<br>1. Prior to conducting the autopsy, collect blood from decedent for serum separator tubes and blood cards. Allow the blood cards to dry completely prior to closing and room temperature storage. Cerebrospinal fluid (CSF) will be obtained via a cisternal tap and frozen.<br>2. Apply the appropriate modified CAP Gross Autopsy protocol (adult), including both gross and microscopic examination, and collect the autopsy common data elements (demographic, external, and internal findings).<br>3. Generate the final anatomic diagnosis (FAD) from a standardized list of approved terms.<br>4. Collect fresh tissue from 50 anatomic sites representing many organ systems as dictated by the Autopsy Template, to be snap frozen (two 1cm cubes) and paraffin embedded (one 1cm cube). Two matched tissue samples from the Autopsy Template should be placed in a tissue cassette, with additional sample types as necessary for viral quantification. From the visceral organs 40 or more sites should be sampled, and from central nervous system (CNS) 15 or more sites should be sampled, including cerebrospinal fluid (CSF).<br>5. Specimens in tissue cassettes will be fixed in neutral buffered formalin (NBF) for approximately 6 hours not to exceed 48 hours.<br><br>**Submission of biospecimens to the RECOVER Biospecimen Core (PBC).** A centralized biorepository has been selected to process and store biospecimens for this study. All autopsy study sites will collect and prepare biospecimens, including blood, body fluids, snap frozen and formalin-fixed tissue samples, as per the protocol, for shipping to the centralized RECOVER biorepository, where all specimens will be processed and stored. In situ hybridization (ISH), ddPCR, and whole slide imaging (WSI) will be performed centrally.<br><br>**Post-mortem MRI studies of CNS.** It is anticipated that 50% or more of patients will be subject to examination of the CNS by autopsy, and will be evaluated with post-mortem MRI. The collection method is outlined in the CNS SOP protocol in the RECOVER Autopsy Manual of Procedures.**Clinical Data.** Abstracted decedent medical records will be linked when possible to RECOVER cohort and EHR information. The following elements of clinical data will be obtained from review of the decedent's EHR and available data collected

	from enrollment and follow-up in the RECOVER adult cohort studies, if applicable.<br>• Electronic Health Records (EHR) SARS-CoV-2 vaccination status<br>• RECOVER study questionnaires, if applicable<br>• Physical examinations<br>• Biomarkers<br>• Biospecimens<br>• Imaging

2 INTRODUCTION, BACKGROUND INFORMATION, AND SCIENTIFIC RATIONALE

2.1 Introduction and Background Information

The severe acute respiratory syndrome coronavirus 2 (SARS-CoV-2) is a novel coronavirus strain that emerged at the end of 2019. This strain primarily spreads through aerosols in expiratory gases in infected individuals with and without symptoms, resulting in the highly contagious coronavirus disease 2019 (COVID-19) and a global pandemic. As of June 2021, approximately 177 million people were infected with COVID-19, with at least 3.8 million deaths globally (3). COVID-19 positive cases are identified with SARS-CoV-2 polymerase chain reaction test or an antigen test using saliva, nasopharyngeal, or bronchial samples (4). Fever, chills, cough, shortness of breath, fatigue, muscle aches, loss of taste and/or smell, nausea, diarrhea, and other symptoms are typical of the acute phase of the disease (5,6). The spectrum of symptoms in acute infection in children is similar to adults, but typically less severe, and with a substantial proportion with little or no symptoms. Chest pain or burning, labored breathing, disorientation, and delirium are less common emergent symptoms in both adults and children associated with increased risk of hospitalization and death. Increased risk of severe COVID-19 disease and death in adults is associated with increased age, (7) male sex, obesity, and co-morbidities such as diabetes, cardiac disease, or cancer (5,8). Additionally, non-White adults and children have experienced much higher rates of infection, severe disease, and death when compared with non-Hispanic Whites (9).

Multi-organ injury and dysfunction in acute COVID-19 have been well-characterized in both adults and children (8,10-12). After recovery from acute COVID-19, 30-70% of those infected report a diverse array of persistent mild to severe symptoms and diseases lasting >4 weeks after the initial SARS-CoV-2 infection. Commonly reported persistent symptoms include fatigue, post-exertional malaise, dyspnea, palpitations, and loss of taste or smell. Stroke, renal failure, myocarditis, neurological syndromes, arterial and venous thromboembolism, and pulmonary fibrosis have been reported to occur >4 weeks after initial COVID-19 infection (5,9,13-20). Persistent or new symptoms and organ dysfunction after COVID-19 infection are now termed post-acute sequelae of SARS-CoV-2 (PASC). The underlying pathophysiology of persistent symptoms after SARS-CoV-2 infection is unknown but has been proposed to be attributable to viral persistence, reactivation of other viruses such as Epstein-Barr virus, vascular endothelial damage, small fiber autonomic nerve damage, neuroinflammation in the central nervous system, immune dysregulation including activation of auto-immunity, and organ damage caused by hyper-inflammatory response during the

acute phase of the disease (9). Other contributing causes include complications from critical illness related to prolonged intubation, prolonged bed rest, and malnutrition, as well as impacts of pandemic-related stressors, disruptions of school, and disruptions of health care access. The interaction of sociodemographic, clinical, and biologic effects is likely to be complex and multifaceted.

The most common symptom after COVID-19 infection is fatigue, closely followed by respiratory symptoms such as shortness of breath, which may occur independent of demonstrable abnormalities in lung structure or function (9,21,22). PASC is multifaceted, with many patients reporting metabolic disorders (21), neurological or nervous system symptoms (7,16,17), cardiovascular symptoms (9,21,22), or gastrointestinal symptoms (9,21). The severity of the acute COVID-19 manifestation has consistently been found to be directly proportional to the severity of post-COVID manifestation, but severe post-acute symptoms have been reported in subjects with mild or asymptomatic acute disease (21). Psychosocial effects such as anxiety, depression, post-traumatic stress disorder, and sleep disturbances are commonly reported in the adult PASC population (6,9,22-25).

Post-mortem analysis of patients dying of COVID-19 uncovered the multi-system nature of the SARS-CoV2 infection. Morphologic investigation and analysis detailed the fatal respiratory infection, viremia, and downstream affected tissues, and characterized systemic tissue destruction. In addition, autopsies provided the answers to the clinical challenges of unexpected and sudden clinical decompensation by showing the macro and microvascular thrombotic manifestations of the disease and endothelial injury. Scientific investigation on the autopsy tissue bolstered the clinical suspicions of cytokine interactions with both the innate and adaptive immune system.(26)

The goal of the PASC autopsy study is to identify, evaluate, and characterize the clinical manifestations of PASC in adults who die following SARS-CoV-2 infection with and without PASC, and the risk factors associated with the severity of these clinical manifestations. This cross-sectional study will focus on the pathological manifestations of SARS-CoV-2 infection, while explicitly considering sex as a biological variable and the impact of racial and ethnic disparities on autopsy findings. Data acquired from this study will provide accurate and quantifiable measures for PASC symptoms in selected populations to allow for comparisons among groups and provide insights into mechanisms related to pathogenesis of PASC and its underlying biology.

2.2 Scientific Rationale

PASC stands to pose a profound public health crisis in future years, with symptoms that can be incapacitating, prevent people from working, and increase the risk of mortality in both

adults and children. As of June 2021, there were over 33 million diagnosed cases of COVID-19 in the United States, certainly an underestimate because of undertesting and underreporting particularly in children **(27,28)**. Given the estimated total number of cases, even a low incidence of PASC would affect millions. However, the incidence, prevalence, phenotypes, risks, and etiology of PASC are currently unknown, limiting opportunities for prevention and treatment **(27)**. Therefore, autopsy studies are urgently needed. This proposed autopsy study will enhance knowledge of the effects of SARS-CoV-2 infections and define and categorize the clinical spectrum of PASC in decedents. Ultimately, we expect that these data will elucidate potential mechanisms to inform future preventive and treatment studies.

3 STUDY OBJECTIVES

The overall scientific objectives of this proposed study are:

1. To enhance knowledge of the effects of SARS-CoV-2 infection;
2. To define and categorize the clinical spectrum of PASC;
3. To elucidate potential mechanisms to inform future preventive and treatment studies.

4 SPECIFIC AIMS

The following specific aims are proposed to achieve these objectives.

Aim 1. Characterize the prevalence and types of organ injury/disease, pathological features, and distinct phenotypes in decedents with acute SARS-CoV-2 infection (dying 15-60 days after infection), and in decedents with prior SARS-CoV-2 infection (dying > 60 days after infection) with and without PASC.

Aim 2. Characterize the association of pathological findings with clinical measures of disease severity and associated risk factors in decedents with prior SARS-CoV-2 infection (dying > 60 days after SARS-CoV-2 infection) with and without PASC.

2a. Characterize the association of COVID-19 disease severity and treatment with pathological findings in decedents with prior SARS-CoV-2 infection with and without PASC .

2b. Determine whether pre-infection and peri-infection risk and resiliency factors (e.g., demographic, biological factors, and preexisting clinical comorbidities) are associated with pathological findings in decedents with prior SARS-CoV-2 infection with and without PASC.

Aim 3. Define the pathophysiology of and mechanisms associated with pathological findings, including the direct and indirect causal effects of clinical risk factors (e.g., comorbidities, infection severity, hospitalization, intubation, steroid treatment) on pathological findings (e.g., inflammation, fibrosis, necrosis) via viral persistence (investigated systematically via molecular methods), in decedents with and without PASC.

Aim 4. Establish a multi-center post-mortem tissue biobank and post-mortem brain imaging bank from decedents with prior SARS-CoV-2 infection with and without PASC. The primary goal of the brain imaging is to find and characterize lesions in the CNS via image-directed sampling of identified lesions. A secondary goal is to correlate decedent's MRI findings with those observed in pre-mortem neuroimaging.

5 STUDY DESIGN

5.1 Overall Study Design

This is a cross-sectional study of decedents who will enter the autopsy cohort at varying stages after infection with SARS-CoV-2 (Figure 1). This study will be conducted in the United States and decedents will be enrolled through interaction with families of the deceased at inpatient, outpatient, and community-based settings. Study data including age, demographics, medical history, vaccination history, details of acute SARS-CoV-2 infection, overall health and physical function, and PASC symptom screen will be collected from the electronic health record using a case report form.

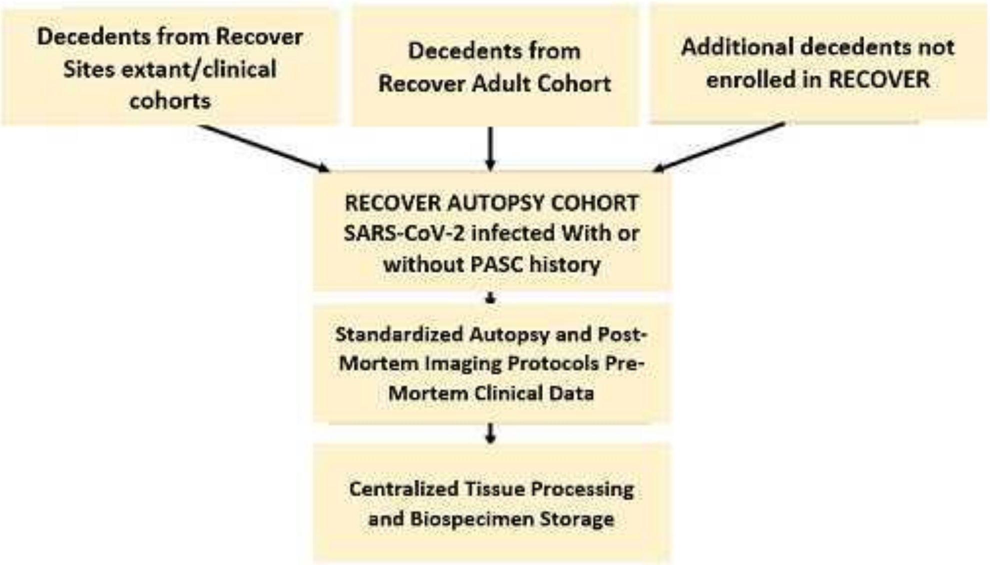

Figure 1: Recruitment Source for RECOVERY Autopsy

Biologic specimens will be collected, with some tests performed in local clinical laboratories and others performed by centralized research centers or banked in the RECOVER Consortium Biospecimen Repository. Advanced clinical examinations and radiologic examinations will be performed at local study sites with cross-site standardization.

5.2 Study recruitment

The SARS-CoV-2 PASC RECOVER autopsy study will enroll individuals with SARS-CoV-2 infection, defined as probable, suspected, or confirmed according to the WHO guideline, and at varying stages after infection. Some autopsy sites will be linked with a clinical RECOVER site and will perform autopsies on RECOVER decedents. Additional decedents from extant cohorts and clinical cohorts at these sites and at other PASC consortium autopsy cohort sites who are not enrolled in RECOVER, but who have relevant clinical data collected, will be identified for possible later enrollment in the autopsy cohort.

Extant, clinical, and de novo (newly enrolled in RECOVER) cohorts:

- SARS-CoV-2 infected individuals with and without current or prior PASC-like symptoms, including infected individuals with MIS-A.

Acute cohort:

- Newly SARS-CoV-2 infected individuals

Infected individuals in the extant and clinical and de novo cohorts will be enrolled after a qualifying initial infection.

If available, relevant retrospective data prior to enrollment will be extracted from the electronic health record or existing extant and clinical cohort data.

Decedents will contribute to aims according to their SARS-CoV-2 infection/PASC history and progression.

Any autopsy sites that are not connected with RECOVER adult cohort study sites will select decedents for protocol autopsies according to eligibility listed below.

5.3 Eligibility Criteria

Decedents over the age of 18 years at the time of death will be eligible for inclusion. Infected individuals will have suspected, probable, or confirmed SARS-CoV-2 infection as defined by WHO criteria..

Patients with suspected SARS-CoV-2 infection

a) Patients who meet the clinical and epidemiological criteria listed below:
   Clinical criteria: Acute onset of fever and cough or acute onset of ANY THREE OR MORE of the following signs or symptoms: fever, cough, general weakness/fatigue, headache, myalgia, sore throat, coryza, dyspnea, anorexia/nausea/vomiting, diarrhea, altered mental status.

   Epidemiological criteria:

   i. Having resided or worked in an area with a high risk of transmission of virus: closed residential, school, or camp settings any time within the 14 days before symptom onset; or

   ii. Having resided or traveled to an area with community transmission any time within the 14 days before symptom onset; or

   iii. Any known household contact or any member of the household working in any health care setting, including within health facilities or within the community, any time within the 14 days before symptom onset.

b) An asymptomatic person not meeting epidemiologic criteria with a positive SARS-CoV-2 Antigen-RDT.

Patients with probable SARS-CoV-2 infection

a) A patient who meets clinical criteria above AND is a contact of a probable or confirmed case or linked to a COVID-19 cluster; or

b) A suspected case with chest imaging showing findings suggestive of COVID-19 disease; or

c) A person with recent onset of anosmia (loss of smell) or ageusia (loss of taste) in the absence of any other identified cause; or

d) Death, not otherwise explained, in an adult with respiratory distress preceding death AND who was a contact of a probable or confirmed case or linked to a COVID-19 cluster.

Patients with confirmed SARS-CoV-2 infection

a) A person with a positive Nucleic Acid Amplification Test (NAAT); or

b) A person with a positive SARS-CoV-2 Antigen-RDT AND meeting either the probable case definition or suspected criteria a) or b); or

c) An asymptomatic person with a positive SARS-CoV-2 Antigen-RDT who was a contact of a probable or confirmed case.

General eligibility notes

i. Decedents with or without history of MIS-A or MIS-C are eligible;
ii. Decedents with or without history of SARS-CoV-2 vaccination are eligible;
iii. Decedents with recurrent SARS-CoV-2 infections and those with post-vaccination (breakthrough) infections are eligible;
iv. Decedents are eligible without exclusion related to sex, race/ethnicity, geography, nationality, severity of disease, or underlying health conditions.

Study recruitment strategy and sampling

Enrollment of decedents with history of SARS-CoV-2 infection will be stratified to ensure adequate representation by age, sex, race/ethnicity. Decedents with history of SARS-CoV-2 infection will be recruited from the adult RECOVER cohorts, from community cohorts of decedents identified at home or in care facilities, and from local medical examiners' offices.

For decedents enrolled after acute infection, preference will be given to those who have data and/or biospecimens collected before or during the acute phase available to the investigators, though efforts should still be made in such cases to recruit an unbiased sample of such decedents (for instance, by oversampling this group for underrepresented minorities or communities not already well represented in the cohort).

5.4 Working Definition of PASC

The initial working definition of PASC will be that published by the World Health Organization (WHO) on October 6, 2021. They define PASC as a condition occurring "in individuals with a history of probable or confirmed SARS-CoV-2 infection, usually 3 months from the onset of COVID-19 with symptoms that last for at least 2 months and cannot be explained by an

alternative diagnosis. Common symptoms include fatigue, shortness of breath, cognitive dysfunction but also others* and generally have an impact on everyday functioning. Symptoms may be new onset following initial recovery from an acute COVID-19 episode or persist from the initial illness. Symptoms may also fluctuate or relapse over time." The list of additional symptoms indicated by the asterisk includes abdominal pain, menstrual and period problems, altered smell/taste, anxiety, blurred vision, chest pain, cough, depression, dizziness, intermittent fever, gastrointestinal issues, headache, memory issues, joint pain, muscle pain/spasms, neuralgia, new onset allergies, pins and needles sensations, post-exertional malaise, sleep problems, tachycardia/palpitations, and tinnitus and other hearing issues.

As the definition of PASC evolves in light of information obtained from the RECOVER Adult Cohort Study, reviewed at quarterly intervals, we will apply the adjusted definitions to decedents in real time.

5.5 Generalizability

To enhance generalizability, a diverse population of decedents will be enrolled. Enrollment will be stratified by age group, sex, and race/ethnicity to ensure ≤ 40% non-Hispanic Whites with the remainder consisting of non-Hispanic Blacks, Hispanic/Latinx, Asian Americans, Native Hawaiians, and Pacific Islanders with equal distributions between male and female populations.

We will enhance generalizability by ensuring that enrolled decedents are drawn from as unbiased a denominator as possible, including community-based recruitment. Because the RECOVER autopsy study does not aim to estimate the incidence of PASC, the threat of bias due to sampling strategies is lower than for the adult and pediatric cohorts.

5.6 Sample Size and Power Calculations

Aim 1. Characterize the prevalence and types of organ injury/disease, pathological features, and distinct phenotypes in decedents with acute SARS-CoV-2 infection dying 15-60 days after infection, and in decedents with prior SARS-CoV-2 infection (dying >60 days after infection) with and without PASC.

Table 1 provides detectable effect sizes and detectable differences in proportions between PASC+ and PASC- individuals for quantitative and dichotomous pathological features.

Date: 7 FEB 2022

Results are given with and without a multiple comparison adjustment (assuming 50 tests, alpha=0.001 conservative control).

Total sample size (not including acute)	Number SARS/ CoV2+/ PASC+	Number SARS/ CoV2+/ PASC-	***Detectable standardized effect size*** for 80% power, alpha=0.05, 0.001, based on two independent group, two-sided comparison of means	***Detectable difference in proportion*** between PASC+ and PASC- groups with 80% power, alpha=0.05, 0.001, based on two independent groups, two-sided comparison of proportions. Proportion with feature = 0.2 in PASC- individuals	Proportion with feature = 0.4 in PASC- individuals
300	200	100	0.34, 0.51	0.15, 0.24	0.17, 0.25
400	200	200	0.28, 0.42	0.13, 0.19	0.14, 0.21
400	267	133	0.30, 0.44	0.13, 0.20	0.15, 0.22
600	300	300	0.23, 0.34	0.10, 0.15	0.12, 0.17
600	400	200	0.24, 0.36	0.11, 0.16	0.12, 0.18
700	350	350	0.21, 0.31	0.09, 0.14	0.11, 0.16
700	467	233	0.23, 0.33	0.10, 0.15	0.11, 0.17
800	400	400	0.20, 0.29	0.09, 0.13	0.10, 0.15
800	534	266	0.21, 0.31	0.09, 0.14	0.11, 0.16
900	450	450	0.19, 0.28	0.08, 0.12	0.10, 0.14
900	600	300	0.20, 0.29	0.09, 0.13	0.10, 0.15

Aim 2. Characterize the association of pathological findings with clinical measures of disease severity and associated risk factors in decedents with prior SARS-CoV-2 infection (dying > 60 days after SARS-CoV-2 infection) with and without PASC.
A sample size of 400 within one group (e.g., PASC+) will have greater than 80% power to detect a small effect size of d=0.24 between individuals with and without the risk factor (within the specified group), assuming 50% of individuals have the risk factor. This is based on a two group, two-sided comparison of means, and controlling type 1 error at 0.05.

To characterize the detectable effect sizes from the subset of decedents who undergo brain imaging, please see the first row of the table. A sample size of 300 provides 80% power to detect a moderate effect size of d=0.34 between individuals with and without the risk factor (within the specified group), assuming 50% of individuals have the risk factor. This is based on a two group, two-sided comparison of means, and controlling type 1 error at 0.05.

6 STUDY METHODS

6.1 Data Sources and Prioritization

Data may be obtained from multiple sources: directly from the decedent's next of kin, from the decedent's existing research records (if participating in an ongoing research study, e.g., RECOVER, extant cohorts, clinical cohorts), from the decedent's electronic health record (EHR) or existing registries, or from insurance claims data. Pre-existing EHR data will be extracted electronically where possible or abstracted and recorded on structured electronic case report forms (eCRF). Unstructured EHR data may be collected when available. Prior to data use, a HIPAA authorization will be obtained from next of kin, subject to the special provisions that apply post-mortem (i.e., to alert law enforcement to the death of the individual, when there is a suspicion that death resulted from criminal conduct; to alert coroners, medical examiners, and/or funeral directors; to conduct research that solely involves the protected health information of decedents; and to assist organ procurement organizations or other entities engaged in the procurement, banking, or transplantation of cadaveric organs, eyes, or tissue for the purpose of facilitating organ, eye, or tissue donation and transplantation). De-identified data collected from any source will be recorded in the PASC Consortium study database with eCRFs. When the same data element is available from multiple data sources, the following hierarchy will be used to represent data:

- For demographics and patient-reported outcomes, existing patient-reported data will be prioritized over EHR, and then claims data;
- For clinical data elements, EHR will take precedence over claims data. Patient-reported data will be given the least weight in this category.

6.2 Methods of data collection

The majority of the study data will be collected at autopsy according to the standardized autopsy protocol described in the appendix. These data will include structured fields for the gross findings. The microscopic findings will be captured as text fields, and will later be systematically coded into approved terms and SNOMED codes; these codes will be finalized following review by the responsible pathologist. Similarly, the FAD will be coded using a standard set of approved terms. Additional EHR and other research data will be extracted and entered into electronic case report forms.

Biospecimen collection will be handled in two ways, depending on stability of the sample:

1) Samples for analytes that require rapid freezing will be processed locally and sent to the central RECOVER biorepository on dry ice;
2) Other samples will be shipped directly to the centralized RECOVER biorepository for processing.

6.3 Return of results to families

Decedent's next of kin will receive the standard clinical autopsy report with final anatomic diagnosis (FAD) if they choose to do so. This report will normally be available to next of kin within 60 working days. Next of kin will not receive any research results from the RECOVER study.

6.4 Strategies for study modifications

This protocol is designed to be pragmatic and flexible in design. We will undertake the following procedures to guide protocol modifications over time:

1) The frequency of PASC will be monitored in real time during the study. If the incidence is found to be lower than anticipated, recruitment strategies will be altered to deliberately oversample PASC cases;
2) Data elements that are not NIH recommended CDE may be modified based on ongoing analysis by DRC; data elements that are not informative to PASC definition models may be removed, with substitution by new data elements;
3) PASC definition will be revised in an iterative manner based on existing PASC data, medical literature, and feedback from patient representatives, participants, and the scientific community.

6.5 Overview of analytic approach to aims

Application of a standardized coding process supports consistency in clinical documentation and reporting. Coding autopsy reports using a standardized, rule-based approach to represent free-text data provides a set of concepts and relationships that offer a common reference point for comparisons and aggregation of data. Such coding will be a key component underlying the analytic strategies outlined below.

Aim 1. Characterize the prevalence and types of organ injury/disease, pathological features, and distinct phenotypes in decedents with acute SARS-CoV-2 infection (dying 15-60 days after infection), and in decedents with prior SARS-CoV-2 infection (dying >60 days after infection) with and without PASC.

We will statistically compare endpoints such as inflammation, fibrosis, thrombosis, and necrosis in individual organs in decedents dying more than 60 days after SARS-CoV-2 infection with PASC symptoms and without PASC symptoms. We will first assess covariate balance between the two groups using standard causal inference techniques (e.g., “Love” plots) and use matched-sampling approaches (e.g., propensity score matching) to reduce any covariate imbalance. We will conduct similar statistical analyses comparing decedents with acute SARS-CoV-2 infection (dying 15-60 days after infection) to decedents with prior

SARS-CoV-2 infection (dying >60 days after infection) without PASC symptoms. The primary analysis will be to determine if there are pathologies specific to PASC that can be correlated with tissue/organ/clinical dysfunction.

Aim 2. Characterize the association of pathological findings with clinical measures of disease severity and associated risk factors in decedents with prior SARS-CoV-2 infection (dying > 60 days after SARS-CoV-2 infection) with and without PASC.

2a. Characterize the association of COVID-19 disease severity and treatment with pathological findings in decedents with prior SARS-CoV-2 infection with and without PASC .

We will estimate conditional associations and 95% confidence intervals (CIs) from multivariate models regressing pathological findings on COVID-19 disease severity adjusting for background covariates (e.g., sex, age, race / ethnicity) and stratifying by PASC status. If the estimated regression coefficients for the background covariates are similar for PASC positive and PASC negative decedents, we will use an interaction term between COVID-19 disease severity and PASC status. We will assess covariate balance between groups defined by COVID-19 disease severity using standard causal inference techniques (e.g., "Love" plots) and use matched-sampling approaches (e.g., propensity score matching) to reduce any covariate imbalance.

2b. Determine whether pre-infection and peri-infection risk and resiliency factors (e.g., demographic, biological factors, and preexisting clinical comorbidities) are associated with pathological findings in decedents with prior SARS-CoV-2 infection with and without PASC.

Similar to Aim *2a*, we will estimate conditional associations and 95% confidence intervals (CI) from multivariate models regressing pathological outcomes on pre-infection and peri-infection risk and resiliency factors (e.g., social determinants of health, demographic, behavioral, biological factors, preexisting clinical comorbidities, and acute infection treatment) and stratifying by PASC status. If the estimated regression coefficients for the background covariates are similar for PASC positive and PASC negative decedents, we will use interaction terms between pre-infection and peri-infection risk and resiliency factors and PASC status. We will assess covariate balance between groups defined by the risk factors using standard causal inference techniques (e.g., "Love" plots) and use matched-sampling approaches (e.g., propensity score matching) to reduce any covariate imbalance.

Aim 3. Define the pathophysiology of and mechanisms associated with pathological findings, including the direct and indirect causal effects of clinical risk factors (e.g., comorbidities, infection severity, hospitalization, intubation, steroid treatment) on pathological findings (e.g., inflammation, fibrosis, necrosis) via viral persistence (investigated systematically via molecular methods), in decedents with and without PASC.

Risk factors for PASC could include viral persistence. We will explore whether clinical risk factors (e.g., comorbidities, infection severity, hospitalization, intubation, or treatments such as steroids) contribute to viral persistence (measured by ddPCR and RNA in situ hybridization). We will conduct a mediation analysis to estimate the direct and indirect causal effects (with 95% CIs) of clinical risk factors such as steroid treatment on pathological outcomes (e.g., histopathologic changes such as inflammation, fibrosis, and/or necrosis) via viral persistence (the possible mediator), in decedents with and without PASC. We will assess covariate balance between groups defined by the clinical risk factors using standard causal inference techniques (e.g., “Love” plots) and use matched-sampling approaches (e.g., propensity score matching) to reduce any covariate imbalance.

Aim 4. Establish a multi-center post-mortem tissue biobank and post-mortem brain imaging bank from decedents with prior SARS-CoV-2 infection with and without PASC. The primary goal of the brain imaging is to find and characterize lesions in the CNS via image-directed sampling of identified lesions. A secondary goal is to correlate decedent’s MRI findings with those observed in pre-mortem neuroimaging.

The biobank will serve as a resource for further investigation of mechanisms and pathophysiology. Biospecimens will be stored in the centralized RECOVER repository for future analyses. The post-mortem images will be stored in a centralized RECOVER repository for future analyses.

Sex as a Biological Variable

The proposed study will enroll both male and female decedents with accrual goal of equal representation by sex. Sex will be used to report disaggregated data, and will be incorporated into multivariable models for assessment of risk and resiliency factors, and as a potential modifier of PASC progression.

6.6 Data Entry and Management

Data Sources

There are six primary sources of data for the entire RECOVER initiative:

1. Prospective observational cohort studies
    a. Adult (15 expected hubs)
    b. Pediatric (8 expected hubs)
    c. Pregnancy (2 expected hubs)
2. Autopsy studies (5 expected hubs)
3. EHR repositories (3 expected hubs)
4. RECOVER biorepository
5. Digital/mobile health platform (Cohort/Non-cohort studies)
6. Data repositories (one each)
    a. Imaging
    b. Pathology

Data Categories

RECOVER study data may be divided into two broad categories: structured and unstructured. Structured data can be simple (e.g., anatomic characterization) or complex (e.g., text-based microscopic findings). REDCap is best suited to capture structured data electronically; i2b2 is best suited to capture complex and unstructured data electronically.

Data Types

RECOVER study data may be divided into at a number of different operational data types.

1. EHR data (unstructured)
2. EHR data (structured, complex)
3. Claims data separate from EHR data (structured, complex)
0. Biorepository inventory data (biospecimens, slides; structured, complex)
1. Biorepository assay data (structured, complex)
2. Advanced imaging data (CT and MRI; unstructured)
    i. Post-mortem MRIs
3. Vaccination status data (structured)
4. Physical examination and gross findings (structured, complex)
5. Whole slide imaging data (unstructured)
    i. Section cut, stained with H&E and scanned (20X resolution)
6. Autopsy visceral sites ddPCR for SARS-CoV-2 virus (structured, complex)
7. Immunoassays and ‘omics data (genomics, proteomics, transcriptomics, metabolomics, meta-genomics microbiome, and epigenetics) (structured, complex)

Data Management Requirements

1. Ability to capture and QC all study data from the study sites in electronic form (EDC) using pre-specified CDEs as appropriate

2. Ability to limit PII/PHI on a Central Hub via UUIDs and/or PPRLs
3. Ability to load and transmit all study data to the DRC
4. Ability to load and transmit select, pertinent historical data to the DRC
5. Ability to maintain a common ontology for all the data coming from each source
6. Ability to maintain provenance of all the data coming from each source
7. Ability to store and/or access all study data across all sources and data types
8. Ability to query and obtain reports on all study data across all sources and data types
9. Ability to maintain metadata (such as consenting status of patients) that can be labelled and managed with common queries
10. Ability to obtain real-time reports on the status of the data at the study sites

Electronic Data Capture (EDC) Methods

Electronic Data Capture Methods for RECOVER			
**Data Type**	**REDCap**	**i2b2**	**RECOVER Data Repository**
Advanced imaging (CT, MRI)		√	**Imaging**
Whole slide imaging		√	**Imaging**
Autopsy visceral sites ddPCR		√	
Prior study (extant) data		√	
Biospecimen data	√ *	√	**Biorepository**
Assays (immunoassays, genomics, proteomics, transcriptomics, metabolomics, meta-genomics microbiome, epigenetics)		√	
Structured EHR data		√	
Unstructured EHR data		√	
Claims data (distinct from EHR data)		√	

*”Simple” data only

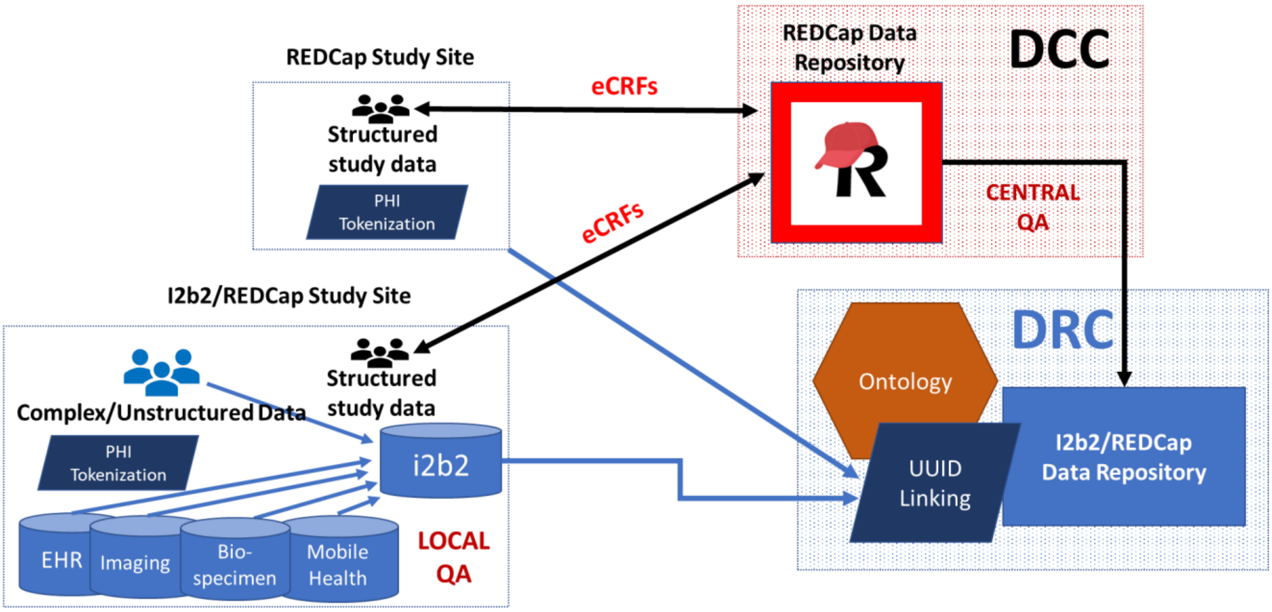

Figure 2 Architecture of Data Flow from Study Sites to the DCC and DRC

<u>**Description of Data Flows**</u>

REDCap study sites using only REDCap Electronic Case Report Forms (eCRFs) will capture data as coded (linked through UUIDs) HIPAA-defined limited data sets within the **REDCap RECOVER Data Repository** at the Data Coordinating Center (DCC). Data QA will occur centrally at the DCC and then the DRC will import the data into a **i2b2/REDCap RECOVER Data Repository**. The Data Dictionary / Ontology for the eCRFs is hosted in both the **REDCap RECOVER Data Repository** and in the **i2b2/REDCap RECOVER Data Repository**. The Universally Unique Identifier (UUID) for each decedent is linked using Protected Health Information (PHI) that is maintained locally by each study site.

i2b2/REDCap RECOVER study sites will be enabled for capturing both structured data via REDCap eCRFs (coded as described above within the **REDCap RECOVER Data Repository**), complex structured data, and unstructured data via a local i2b2 data portal. The UUID for each decedent will be linked using Protected Health Information (PHI) that is maintained locally by each study site, and the DRC will import data directly into its **i2b2/REDCap RECOVER Data Repository**. The Data Dictionary / Ontology for the multi-dimensional data will be hosted in the DRC Central i2b2 data RECOVER repository. Regardless of how the data are captured and ingested by the DRC, it will be made interoperable through common data dictionaries / ontologies and UUIDs that are maintained by the DRC, and via the Common Data Model (CDM) in the DRC's RECOVER i2b2 data repository. Ultimately the i2b2/REDCap study sites will be able to query and create reports from these data.

7 ETHICAL CONSIDERATIONS

7.1 Research Involving the Dead

All procedures conducted as part of this protocol will follow the principles outlined in the report of The Committee for the Oversight of Research Involving the Dead.(29) These include, but are not limited to, dignity, requirement for reasonable scientific validity, confidentiality, consent, non-interference with organ procurement for donation, and protection from harm.

7.2 Informed Consent Process

Informed consent for autopsy and HIPAA authorization will be obtained and documented in writing from next of kin (autopsy) and legally authorized representative (HIPAA authorization) prior to study entry. Study sites will identify potential decedents during RECOVER follow-up. The research study will be explained in lay terms to the decedent's next of kin in their preferred language. The consent process may be conducted by telephone, video conference, or in person. The investigator or suitable delegate listed on the Investigator of Record Form will conduct a meeting with the decedent's next of kin to review all relevant topics related to research involving the recently dead, including HIPAA considerations for deceased individuals. Documentation of process for obtaining autopsy consent and HIPAA authorization will be recorded electronically in the research record.

7.3 Potential Risks

Participation in the study is associated with a small risk of breach of confidentiality. Study procedures to reduce risk of breach are described below.

Genetic Testing

There is a risk of discovering potentially pathogenic genetic variants of unclear clinical significance during the study. The RECOVER Tissue Pathology study will not share any genetic results with the decedent's next of kin or legal representatives. The study will include a statement in the consent form and in the accompanying information sheet apprising the next of kin that they will not be offered any report on whether their relative was found to carry a clinically actionable potentially pathogenic genetic change. For next of kin who inquire about information on genetic results, they will be informed that they have an option to request a copy of the clinical autopsy final report, but that any genetic variant discovered will not be shared with the site PI or designated study personnel.

7.4 Confidentiality, Protection against Risks

Investigators in this research will take all reasonable measures to protect the confidentiality of the medical records of patients and their families. Measures to protect confidentiality are as follows:

7.4.1 Storage of Study Materials

Investigators will take all reasonable measures to protect the confidentiality of the study decedents through the measures used in all PASC studies, including storage of study materials in locked, secure locations accessible only to study investigators, and knowledge of the subject's name only at the local institution. In addition, tissue specimens stored locally or submitted to the PASC Biorepository Core will be labeled with only the unique RECOVER study ID, and will not include any patient identifiers such as name or initials.

7.4.2 Hashed identifiers

A subject-specific universally unique identifier (UUID) will be assigned to each study decedent. The hashed identifier is a universal subject ID that allows researchers to share data specific to a study decedent without exposing personally identifiable information (PII), while also enabling linking of decedents across labs, databases, or research studies. Personal information will not leave the research site, but rather will be used only to generate a unique set of encrypted codes that can then be decrypted to determine if the decedent already exists within a data repository. The hashed identifier will allow data from this study to be combined with data from other research studies or databases.

7.5 Certificate of Confidentiality

To enhance protection of the privacy of decedents participating in the RECOVER biorepository, we will obtain a Certificate of Confidentiality from the National Institutes of Health (NIH). With this Certificate, the researchers of this study cannot be forced to disclose information that may identify a decedent, even by a court subpoena, in any federal, state, or local civil, criminal, administrative, legislative, or other proceedings. The Certificate cannot be used to resist a request for information from the United States government when it is used for evaluating federally funded study projects or for information that must be disclosed to meet the requirements of the Food and Drug Administration (FDA). A Certificate of Confidentiality does not prevent a decedent's family from voluntarily releasing information about the decedent's involvement in this research. If an insurer, employer, or other person

obtains a family's written consent to receive research information, then the researchers will not use the Certificate to withhold that information.

Information from DNA analyses and clinical studies or medical records may be placed into a central data repository in the future, such as the National Center for Biotechnology Information repository. Data and samples will be de-identified before submission to this or any other central repository.

7.6 Potential Benefits

For most families of decedents in this observational study, there will be no direct benefit. Although an individual family may not benefit from participation, the results of the study will make important contributions to the understanding of the long-term outcomes after SARS-CoV-2 infection.

An indirect benefit may come from the awareness that the results of this study may serve to help improve care of people with similar problems in the future. Families may derive a sense of altruism, accomplishment, and contribution to furthering understanding of PASC through their participation.

At the end of the study, decedents' families will be sent a description of the overall study results in lay language. In this study, families will be given the contact information for the study PI and/or coordinator, in the event the they wish to discuss the results or have questions. If desired, a separate notification will also be provided to the treating/referring physician (cardiologist, etc.) describing overall study results.

7.7 Risk/Benefit Ratio and Importance of Information to Be Obtained

The risk/benefit ratio is favorable for this study. There is no risk to the decedent. The study procedures for data collection present minimal risk for the family of the decedent. Although the individual decedent's family may not benefit from participation, the results of this study will make important contributions to the design of optimal management algorithms for people with history of SARS-CoV-2 infection. Data generated from this study will help provide guidance to providers and people who have had evidence of PASC after SARS-CoV-2 infection.

7.8 Women and Minority Inclusion in Clinical Research

COVID-19 disproportionately affects individuals of underrepresented minorities. All eligible decedents will be enrolled in the PASC study without regard to gender, race, or ethnicity. Stratification and sampling will be use to enhance ensure that diversity in the study population is representative of the population with COVID-19.

7.9 Data Safety Monitoring Plan

The Data and Safety Monitoring Plan for this trial will follow recommended monitoring principles for an observational study of a vulnerable population. Oversight of data and safety will be provided by a PASC Observational Safety Monitoring Board appointed by NHLBI. The OSMB will meet at least twice a year to review data on AEs, adverse reactions, suspected adverse reactions, unanticipated events, patient-reported outcomes, data quality, and study recruitment as described in the committee charter, and make recommendations about study conduct to the NHLBI. As the PASC study does not involve any interventions, an early stopping rule for efficacy or futility is not indicated.

After each OSMB meeting, the OSMB determination letter and a summary report of adverse events will be prepared within 30 days and will be distributed by NHLBI staff to each principal investigator and study coordinator for review. The summary report will contain the following information:

A statement that OSMB review of outcome data, adverse events, and information relating to study performance across all centers took place on a given date;

A statement as to whether or not the frequency of adverse events exceeded what was expected and indicated in the informed consent;

A statement that a review of recent literature relevant to the research took place;

The OSMB's recommendation with respect to progress or need for modification of the protocol or informed consent. If the OSMB recommends changes to the protocol or informed consent, the rationale for such changes and any relevant data will be provided;

A statement noting that if safety concerns are identified, the NHLBI Program Official will communicate these promptly to the investigators.

8 STUDY POLICIES

8.1 Conflict of Interest

All recipient institutions and investigators in the PASC consortium will comply with the requirements of 42 CFR 50, Subpart F, "Responsibility of Applicants for Promoting Objectivity in Research for which PHS Funding is Sought" (FCOI Regulation), as implemented in the 2011 Final Rule for grants and cooperative agreements.

The requirements promote objectivity in research by establishing standards that provide a reasonable expectation that the design, conduct, and reporting of research funded under PHS grants or cooperative agreements will be free from bias resulting from any conflicting financial interest of an investigator. An "investigator" is someone defined as the PD/PI and any other person, regardless of title or position, who is responsible for the design, conduct, or reporting of research funded by PHS, or proposed for such funding; investigators may also include collaborators or consultants.

Each institution shall maintain an up-to-date, written, enforced policy on financial conflicts of interest that complies with the regulation and make the policy available via a publicly accessible Web site.

These FCOI requirements do not apply to Federal employees or Federal agencies. Federal agencies have their own set of rules governing financial conflicts of interest for employees.

When submitting a grant application, the signature of the Authorized Organization Representative (AOR) will certify each PASC Consortium applicant institution's compliance with the requirements of 42 CFR 50, Subpart F, including that:

- There is in effect at the institution an up-to-date, written, and enforced administrative process to identify and manage Financial Conflicts of Interest (FCOI) with respect to all research projects for which NIH funding is sought or received;
- The institution shall promote and enforce investigator compliance with the regulation's requirements including those pertaining to disclosure of Significant Financial Interests;
- The institution shall identify and manage FCOIs and provide initial and ongoing FCOI reports to the NIH consistent with this subpart;
- When requested, the institution will promptly make information available to the NIH/HHS relating to any investigator disclosure of financial interests and the institution's review of, and response to, such disclosure, whether or not the disclosure resulted in the institution's determination of an FCOI;

- The institution shall fully comply with the requirements of the regulation.

8.2 Publications

The objectives of the publications and presentations editorial policy are to assure and uphold:

- Expeditious and timely dissemination to the scientific community of all pertinent data resulting from the PASC Consortium RECOVER protocols;
- Accurate and scientifically sound publications and presentations from the PASC Consortium investigators;
- Promotion of and encouragement for analysis and submission of manuscripts among the PASC Consortium investigators.
- A system for fair determination of authorship on PASC Consortium collaborative publications.
- Opportunities for investigators from all participating PASC Consortium cohort sites to participate and be recognized in study-wide publications and presentations; and
- Procedures that allow the NIH to review in a timely fashion publications and presentations summarizing data collected during the course of the trial.

A publications committee will be appointed to establish transparent standard operating procedures to meet these policy objectives in the oversight of publications based on PASC Consortium data.

8.3 Monitoring board

Oversight of data and safety for all three RECOVER cohort studies will be provided by a PASC Observational Safety Monitoring Board appointed by NHLBI. Relevant to the autopsy study, the OSMB will meet at least twice a year to review data on data quality and study recruitment as described in the committee charter, and make recommendations about study conduct to the NHLBI. As the PASC study does not involve any interventions, an early stopping rule for efficacy or futility is not indicated.

After each OSMB meeting, the OSMB determination letter and a summary report of adverse events will be prepared within 30 days and will be distributed by NHLBI staff to each principal investigator and study coordinator for review. The summary report will contain the following information:

- A statement that an OSMB review of outcome data and information relating to study performance across all centers took place on a given date;
- A statement that a review of recent literature relevant to the research took place;

- The OSMB's recommendation with respect to progress or need for modification of the protocol or informed consent. If the OSMB recommends changes to the protocol or informed consent, the rationale for such changes and any relevant data will be provided;
- A statement that if concerns are identified, the NHLBI Program Official will communicate these promptly to the investigators.

9 RESOURCE SHARING PLAN

All resultant data obtained under the aegis of the PASC Consortium will be shared in conformance with the requirements described in the NIH Final Statement on the Sharing of Research Data. Final research data will consist of the recorded factual material commonly accepted in the scientific community as necessary to document, support, and validate research findings. These materials will include all computerized datasets upon which accepted publications are based, and may contain raw data or derived variables, both of which would be described in the documentation associated with the dataset. The content, documentation, and data storage format of the limited access data set will conform to NIH requirements. We are aware of the need to protect human subject information and protected health information in accordance with HIPAA standards and in other situations where data sharing may not be appropriate or allowed. PASC Consortium investigators and administrators have made remarkable efforts to protect the identity of PASC Consortium study participants and family members. We will adhere to these principles in the PASC Consortium longitudinal study of enrolled participants.

Data products from this study will be made available to researchers and analysts through PASC Consortium Data Resource Core. User registration will be required in order to access or download files. As part of the registration process, users must agree to the conditions of use governing access to the public release data, including restrictions against attempting to identify study participants, assurances of destruction of the data after analyses are completed, reporting responsibilities, restrictions on redistribution of the data to third parties, and proper acknowledgement of the data resource. Registered users will receive user support, as well as information related to errors in the data, future releases, workshops (if held), and publication lists. The information provided to users may not be used for commercial purposes and may not be redistributed to third parties. We will take steps to protect the proprietary information of our institutions and those of any collaborators or other participating third parties.

With regard to data sharing for imaging studies, data that are received from external sites and/or collected from internal hospital data sources will be stored on a redundantly backed up server that is located behind the host institution firewall. Access to the server file system will be limited to authorized users only. All access and logins to the servers will be logged. During the upload process, any patient health information (PHI) will be removed from the uploaded dataset. The process will also include elimination of PHI that may reside in the image metadata. Every uploaded dataset will have a secondary manual review to ensure no PHI is stored on hospital servers. Any temporary breaches of PHI will be remedied and logged. All IRB approved collaborators at participating sites will be given unique user ids. All users will be able to share and view data among themselves on the web interface. Users at all sites will be able to run batch analyses through popular pipelines. Incorporation of other imaging analysis software will also be supported if there is consensus across the consortium. When image analysis is performed, the site providing analysis will log operational parameters, assuring complete reproducibility of data analysis. Processed data sets and results of the data analysis will be shared with all sites for secondary analysis. No data will physically leave the Core Lab file servers for batch data processing. In addition, the Core Labs will quantify and summarize de-identified reports and store individual level summary data in the RECOVER Consortium Data Resource Core, or other RECOVER Consortium Data Repository.

During the course of the study, we anticipate that we will perform ancillary studies with other consortia or networks, as well as individual investigators, in order to delineate the fundamental mechanisms of disease that contribute to clinical outcomes. These projects will follow the PASC Consortium ancillary studies policy. Early in the project period, we expect to develop a set of principles for the development and review of joint projects; delineate the processes for sharing clinical, genomic, and other basic science data between consortia or individual centers; and ensure that PASC investigators have the opportunity to participate and be recognized in research projects resulting from collaboration with other investigators or networks.

Recognizing that the value of data often depends on their timeliness, every effort will be made to release and share data upon the acceptance for publication of the main findings from any dataset in accordance with the governance structure of the PASC Consortium.

10 APPENDICES

10.1 Gross Autopsy Template

PASC Autopsy Template

PATIENT IDENTIFICATION AND CONSENT FOR AUTOPSY

Patient name:	[***]
Consent and patient ID reviewed by	[Dr. *** | ***]
Two unique patient identifiers reviewed	[Patient Name: ***]<br>[Date of birth: *** | MRN: *** | ***]
Type of autopsy	[Complete | Brain only | No head | Chest only | Abdomen only | ***]
Name of consenter	[***]
Relationship to the deceased	[***]
Post Mortem Interval	[***] hours
Ante-mortem Imaging	[MRI; CT; Organ] [Finding]
Sars-CoV2 Nasopharyngeal PCR at point of diagnosis	[positive/negative/not available/unconfirmed] [cycle threshold if positive]
Severity of disease	[NIH Scale: mild; moderate; severe]
Severity of disease	[hospitalization; yes or no]
Severity of pulmonary disease	[Method of oxygenation] [none, nasal cannula, mask, high flow, ventilator]
Treatment for infection/PASC	[medication; monoclonal antibody; other]
Non-PASC medications	[medication names]
Global Health Scale (1-5)	
Chronic medical history	[ICD-10]
Sars-CoV2 Nasopharyngeal PCR at collected autopsy	[positive/negative] [cycle threshold if positive]
Sars-CoV2 variant	
Cohort	[15-60; >60 no PASC; >60 PASC; MIS-A]
PASC symptoms	[POTS, PASC symptom checklist]
IgG spike Ab	[positive/negative]
IgG nucleocapsid Ab	[positive/negative]
Vaccination status	[yes; no] [type; date]
Geocode, place of death	
Geocode, zip code address	

The autopsy will be performed using standard biosafety practices and personal protective equipment.
Tissues will be Snap frozen, 1 cm cubes at -70 C, as indicated by the biobanking/biospecimen protocol. If tissues are optionally taken for OCT or RNA later biobanking, please note.
Tissues will be sampled accordingly, placed in a tissue cassette and fixed in 10% buffered formalin for 24 hours and not to exceed 48 hours followed by paraffin embedding. Any fixation in excess of 48 hours is to be noted in the protocol. All tissue sampling should be performed using clean sterile blades.

Date: 7 FEB 2022

Blood collection (aorta)	2 serum separator tubes
Blood card	4 spots
Sural nerve	Snap Frozen and FFPE
Peroneal nerve	Snap Frozen and FFPE
Skin, calf including subcutis, 5 mm punch	Snap Frozen and FFPE
Muscle, gastrocnemius	Snap Frozen and FFPE
Muscle, psoas (proximal)	Snap Frozen and FFPE

EXTERNAL APPEARANCE

Race	[Black | Asian | White | Native American | Asian Pacific Islander | ***]
Sex	[***]
Weight	[***] kg
Height	[***] cm
BMI	***
Hair	[None | Balding | Short| Medium| Long| Black | Brown | Blonde]
Sclerae	[Icteric | Anicteric]
Ears	[Normal]
Nose	[Normal]
Oral cavity	[Good dentition | Poor dentition | Dentures | No teeth | Exam not performed due to rigor]
External genitalia (phenotypic)	[male | female | ambiguous | intersex]
Toe-/ fingernails	[Normal | Onychomycosis | Splinter hemorrhages | Cyanotic]
Edema	[None | Peripheral | Generalized]
Skin	[Scars| Incisions| Lesions]
Evidence of therapy	[None | NG tube | PEG tube | ET tube | Foley catheter| Rectal tube| IV catheters]
Back	[No lesions| Decubitus ulcer, depth]

INCISIONS AND BODY CAVITIES

Tissue fixation time	
Incision	[Standard Y-shaped | Biparietal | ***]
Organs in normal anatomic positions	[Yes | No, describe]
Peritoneal fluid	[None | *** ml | Serous | Cloudy | Serosanguinous | Sanguinous]
Peritoneal surfaces	[Smooth | Adhesions]
Pleural fluid	Right: [None | *** ml | Serous | Serosanguinous | Sanguinous]<br>Left: [None | *** ml | Serous | Serosanguinous | Sanguinous]
Pleural cavity	Right: [Smooth | Adhesions]<br>Left: [Smooth | Adhesions]
Cultures taken	[organ; location - result]

EYES

Eyes	[Submitted | Not submitted]

Date: 7 FEB 2022

MUSCULOSKELETAL SYSTEM

Ribs:	[Fractures | No fractures]
Diaphragm	[Normal | lesions]
Skeletal muscles	[Red-brown and firm | Appropriate mass for age/gender | Atrophied]
Vertebral column	[Normal curvature | Kyphosis | Scoliosis]

DIGESTIVE SYSTEM (Bowel Examination)

Appendix	[Present | Surgically absent |Lesions?]
Small bowel	[Usual caliber | Dilated | Stricture | Contents?| Serosa?| Mucosa?| Lesions, describe location]
Large bowel	[Usual caliber | Dilated | Stricture | Contents?| Serosa?| Mucosa?| Lesion, describe location]
Duodenum	Snap frozen and FFPE
Ileum	Snap frozen and FFPE
Colon, right	Snap frozen and FFPE
Colon, descending/sigmoid	Snap frozen and FFPE
Stool, if available	Snap frozen

CENTRAL NERVOUS SYSTEM

Brain weight:	[***] g, REF RANGE: 1100 - 1700 g
Cerebral hemispheres	[Symmetric | Lesions, describe size and location]
Hemorrhage	[None | Epidural | Subdural | Subarachnoid | ***]
Circle of Willis	[No atherosclerosis or describe degree and location]
Herniation	[None | Present, describe location]
Dura	[Normal]
Cerebral cortex atrophy	[Present | Absent] [if present Mild | Moderate | Severe] [if present Frontal | Parietal | Temporal | Occipital]
Hippocampus atrophy	[Present | Absent] [if present Mild | Moderate | Severe]
Substantia nigra hypopigmentation	[Present | Absent] [if present Mild | Moderate | Severe]
Locus ceruleus hypopigmentation	[Present | Absent] [if present Mild | Moderate | Severe]
Olfactory bulb	Snap frozen and FFPE
Frontal cortex with leptomeninges	Snap frozen and FFPE
Basal ganglia	Snap frozen and FFPE
Thalamus	Snap frozen and FFPE
Hippocampus	Snap frozen and FFPE
Occipital cortex	Snap frozen and FFPE
Amygdala	Snap frozen and FFPE
Pons	Snap frozen and FFPE
Choroid plexus	Snap frozen and FFPE
Medulla (area postrema)	Snap frozen and FFPE
Cerebellum with dentate	Snap frozen and FFPE
Dura with sinus	Snap frozen and FFPE

Spinal cord (thorax) with dorsal root ganglia	Snap frozen and FFPE
Sympathetic chain with ganglia	Snap frozen and FFPE

A neuropathologic examination will be performed on all decedents in whom consent is obtained for the brain and spinal cord. The algorithm for post-mortem brain imaging is defined in detail in the Manual of Procedures. The scan time for the post-mortem brain imaging will not exceed 90 minutes.

ENDOCRINE SYSTEM

Right adrenal weight	[***] g AVERAGE: 6 g (trimmed)
Left adrenal weight	[***] g AVERAGE: 6 g (trimmed)
Adrenal parenchyma	[Uniform yellow cortices | Good demarcation from the medullae | Autolyzed]
Thyroid weight	[***] g, REF RANGE: 30-70 g
Thyroid parenchyma	[Symmetric | Red-brown | Firm | Lesions, describe location and size]
Adrenal	Snap frozen and FFPE

CARDIOVASCULAR SYSTEM

Aorta (Ascending, Thoracic, Abdominal)	[No atherosclerosis | Mild atherosclerosis | Moderate atherosclerosis | Severe atherosclerosis]
Venae cavae	[Thin walled and patent | Thrombi present]
Pulmonary artery	[Normal | Contains embolus]
Pericardium	[Intact | Adhesions | No adhesions]
Pericardial fluid	[None | *** ml | Serous | Sanguinous]
Heart weight	[***] g, REF RANGE: *** g - ***g
Coronary arteries	
LM/LAD	[Minimal| Mild| Moderate| Severe Stenosis| Calcified, describe location]
LCX	[Minimal| Mild| Moderate| Severe Stenosis| Calcified, describe location]
RCA	[Minimal| Mild| Moderate| Severe Stenosis| Calcified, describe location]
Coronary circulation	[Right dominant | Left dominant | Co-dominant]
Coronary ostia	[Normally positioned | Patent]
Foramen ovale	[Probe patent | Closed]
Chamber dilation	[Yes, describe | No]
Valves	
Tricuspid	[***] cm REF RANGE: 10.0 - 12.5 cm
Abnormalities: valve leaflets/chordae	[None- thin and delicate | thickened, degree| lesions, describe location]
Pulmonic	[***] cm REF RANGE: 7.0 - 9.0 cm
Abnormalities: valve cusps	[None- thin and delicate | thickened, degree| lesions, describe location]
Mitral	[***] cm REF RANGE: 8.0 - 10.5 cm

Date: 7 FEB 2022

Abnormalities: valve leaflets/chordae	[None- thin and delicate | thickened, degree| lesions, describe location]
Aortic	[***] cm REF RANGE: 6.0 - 7.5 cm
Abnormalities: valve cusps	[None- thin and delicate | thickened, degree| lesions, describe location]
Left ventricular free wall	[***] cm NORMAL: Less than 1.5 cm
Septum	[***] cm NORMAL: Less than 1.5 cm
Right ventricular free wall	[***] cm NORMAL: Less than 0.5 cm
Epicardium	[Smooth and thin | Thickened | ***]
Myocardium	[Firm and red brown | lesions, describe size and location]
Aorta,ascending	Snap frozen and FFPE
Right ventricle, posterior	Snap frozen and FFPE
Left ventricle, lateral	Snap frozen and FFPE
Left ventricle, nasal	Snap frozen and FFPE
Right atrium/SVC (near SA node)	Snap frozen and FFPE
Left atrium	Snap frozen and FFPE
Coronary artery, right	Snap frozen and FFPE
Coronary artery, left	Snap frozen and FFPE
Lesional tissue [describe]	Snap frozen and FFPE

RESPIRATORY SYSTEM

Epiglottis, larynx, trachea	[No lesions or describe]
Right lung weight	[***] g, REF RANGE: 360 - 570 g
Left lung weight	[***] g, REF RANGE: 325 - 480 g
Fixation	[Fixed in distention | Cut fresh]
Right lung parenchyma	[Soft and pale red | Consolidated| Lesions, describe location]
Left lung parenchyma	[Soft and pale red | Consolidated | Lesions, describe location]
Bronchi	[No lesions | obstructed, describe]
Vasculature	[Pulmonary emboli, describe location | Atherosclerosis]
Bronchus viral swab	Swab
Hilar/mediastinal lymph nodes	Snap frozen and FFPE
Trachea	Snap frozen and FFPE
Right upper lobe, with pleura	Snap frozen and FFPE
Right middle lobe	Snap frozen and FFPE
Right lower lobe	Snap frozen and FFPE
Left upper lobe, with pleura	Snap frozen and FFPE
Left lower lobe	Snap frozen and FFPE
Lesional tissue [describe]	Snap frozen and FFPE

LYMPHORETICULAR SYSTEM

Spleen weight	[***] g REF RANGE: 150 - 200 g unless over 80 years old, then AVERAGE 100 g
Spleen	[Smooth and intact | Dark red, firm or soft | lesions, describe]
Bone marrow	[Normal| lesions, describe]
Lymph nodes	[Not enlarged | Lymphadenopathy, describe location]

Spleen	Snap frozen and FFPE
Bone marrow (rib squeeze)	Snap frozen and FFPE

DIGESTIVE SYSTEM

Tongue	[No lesions or describe]
Esophagus	[Pale tan mucosa with no lesions or describe]
Squamocolumnar junction	[Sharply defined | Indistinct]
Stomach	[Empty | Distended | Contains partially digested food and liquids | Mucosal lesions]
Superior mesenteric artery	[No | Minimal| Mild | Severe atherosclerosis| Patent]
Pancreatic/common bile duct	[Patent| Obstructed, describe]
Gallbladder	[Surgically absent| Thin walled containing green viscous bile with black | yellow calculi| no calculi]
Liver weight	[***] g REF RANGE: 1500 - 1800 g
Liver	[Smooth and glistening | Slightly firm | Firm | Soft| Maroon-brown | Green tinged | Yellow orange | Rusty brown | Mottled red | Nodular | Lesions, describe location and size]
Pancreas dimensions	[*** , give 3 dimensions] cm AVERAGE: 23.0 x 4.5 x 3.8 cm
Pancreas	[Tan | Firm and lobulated | Fatty replaced| Autolyzed| Lesions, describe location and size]
Pancreas	Snap frozen and FFPE
Liver, right lobe (central)	Snap frozen and FFPE
Mesenteric fat	Snap frozen and FFPE

URINARY SYSTEM

Bladder	[Collapsed | *** ml of urine | congested mucosa| Trabeculated]
Ureters	[Patent | Not patent | Dilated | Not dilated | ***]
Renal arteries	[Patent| No| Mild| Moderate| Severe Atherosclerosis]
Right kidney	[Smooth | Granular and pitted | Thin (<8-10 mm) or normal cortical thickness | Clearly- or Ill-defined corticomedullary junctions | Red-brown| lesions describe location and size]
Left kidney	[Smooth | Granular and pitted | Thin (<8-10 mm) or normal cortical thickness | Clearly- or Ill-defined corticomedullary junctions | Red-brown| lesions describe location and size]
Right kidney weight	[***] g
Left kidney weight	[***] g
REF RANGE combined kidney weight	230 - 440 g
Kidney, right or left	Snap frozen and FFPE

Lesional tissue [describe]	Snap frozen and FFPE

GENITAL SYSTEM – MALE/FEMALE

Prostate (3 dimensions/describe)	[*** x *** x ***cm| Normal| Enlarged| Nodular]
Testes	[Normal size | Enlarged | Brown parenchyma | Tubules string in normal manner | Tubules do not string]
Ovaries (3 dimensions)	[*** x *** x ***cm]
Uterus	[Normal | enlarged | atrophic]
Testis	Snap frozen and FFPE
Ovary	Snap Frozen and FFPE
Fallopian tube	Snap Frozen and FFPE

ADDITIONAL CASSETTES:
[***]

11 REFERENCES

1. NIH. NIH Central Resource for Grants and Funding Information.
2. Pentz RD, Cohen CB, Wicclair M et al. Ethics guidelines for research with the recently dead. Nat Med 2005;11:1145-9.
3. Worldometer. Coronavirus Cases. 2021.
4. Carbone M, Lednicky J, Xiao S-Y, Venditti M, Bucci E. Coronavirus 2019 Infectious Disease Epidemic: Where We Are, What Can Be Done and Hope For. J Thorac Oncol 2021;16:546-571.
5. Nasserie T, Hittle M, Goodman SN. Assessment of the Frequency and Variety of Persistent Symptoms Among Patients With COVID-19: A Systematic Review. JAMA Network Open 2021;4:e2111417-e2111417.
6. Higgins V, Sohaei D, Diamandis EP, Prassas I. COVID-19: from an acute to chronic disease? Potential long-term health consequences. Critical Reviews in Clinical Laboratory Sciences 2020:1-23.
7. Wiersinga WJ, Rhodes A, Cheng AC, Peacock SJ, Prescott HC. Pathophysiology, Transmission, Diagnosis, and Treatment of Coronavirus Disease 2019 (COVID-19): A Review. JAMA 2020;324:782-793.
8. Gupta A, Madhavan MV, Sehgal K et al. Extrapulmonary manifestations of COVID-19. Nature Medicine 2020;26:1017-1032.
9. Nalbandian A, Sehgal K, Gupta A et al. Post-acute COVID-19 syndrome. Nature Medicine 2021;27:601-615.
10. Wadman M, Couzin-Frankel J, Kaiser J, Matacic C. A rampage through the body. Science 2020;368:356-360.
11. Barth RF, Xu X, Buja LM. A Call to Action: The Need for Autopsies to Determine the Full Extent of Organ Involvement Associated With COVID-19. Chest 2020;158:43-44.
12. Hannah E. Davis GSA, Lisa McCorkell, Hannah Wei, Ryan J. Low, Yochai Re’em, Signe Redfield, Jared P. Austin, Athena Akrami. Characterizing Long COVID in an International Cohort: 7 Months of Symptoms and Their Impact. medRxiv 2020.
13. Kamal M, Abo Omirah M, Hussein A, Saeed H. Assessment and characterisation of post-COVID-19 manifestations. Int J Clin Pract 2021;75:e13746-e13746.
14. Türktaş H, Oğuzülgen İ K. [Post-COVID-19 pulmonary sequla: longterm follow up and management]. Tuberkuloz ve toraks 2020;68:419-429.
15. Asly M, Hazim A. Rehabilitation of post-COVID-19 patients. The Pan African medical journal 2020;36:168-168.
16. Davido B, Seang S, Barizien N, Tubiana R, de Truchis P. 'Post-COVID-19 chronic symptoms' - Author's reply. Clinical microbiology and infection : the official publication of the European Society of Clinical Microbiology and Infectious Diseases 2021;27:495-496.
17. Tarawneh O, Tarawneh H. Immune thrombocytopenia in a 22-year-old post Covid-19 vaccine. American journal of hematology 2021;96:E133-e134.
18. Wijeratne T, Crewther S. Post-COVID 19 Neurological Syndrome (PCNS); a novel syndrome with challenges for the global neurology community. Journal of the neurological sciences 2020;419:117179.
19. Dani M, Dirksen A, Taraborrelli P et al. Autonomic dysfunction in 'long COVID': rationale, physiology and management strategies. Clinical medicine (London, England) 2021;21:e63-e67.
20. Health F. A Detailed Study of Patients with Long-Haul COVID -An Analysis of Private Healthcare Claims. A FAIR Health White Paper, June 15, 2020.
21. Al-Aly Z, Xie Y, Bowe B. High-dimensional characterization of post-acute sequelae of COVID-19. Nature 2021;594:259-264.
22. Group TWCftCS. Four-Month Clinical Status of a Cohort of Patients After Hospitalization for COVID-19. JAMA 2021;325:1525-1534.

23. Bo H-X, Li W, Yang Y et al. Posttraumatic stress symptoms and attitude toward crisis mental health services among clinically stable patients with COVID-19 in China. Psychol Med 2021;51:1052-1053.
24. Danet Danet A. Psychological impact of COVID-19 pandemic in Western frontline healthcare professionals. A systematic review. Medicina Clínica (English Edition) 2021;156:449-458.
25. Perrin R, Riste L, Hann M, Walther A, Mukherjee A, Heald A. Into the looking glass: Post-viral syndrome post COVID-19. Medical hypotheses 2020;144:110055.
26. Maiese A, Manetti AC, La Russa R et al. Autopsy findings in COVID-19-related deaths: a literature review. Forensic Sci Med Pathol 2021;17:279-296.
27. Stokes EK ZL, Anderson KN, et al. Coronavirus Disease 2019 Case Surveillance — United States,. MMWR Morb Mortal Wkly Rep 2020;69:759–765 CDC, January 22–May 30, 2020.
28. CDC. United States COVID-19 Cases, Deaths, and Laboratory Testing (NAATs) by State, Territory, and Jurisdiction. COVID cases, Deaths and Testing, June 26th 2021.
29. DeVita MA, Wicclair M, Swanson D, Valenta C, Schold C. Research involving the newly dead: an institutional response. Crit Care Med 2003;31:S385-90.
