# Supplementary material for: Researching COVID to enhance recovery (RECOVER) tissue pathology study protocol: Rationale, objectives, and design
Source: PLoS One. 2024 Jan 10;19(1):e0285645. doi: 10.1371/journal.pone.0285645 (PMC10781091; doi:10.1371/journal.pone.0285645)
Supplement: S1 File — (PDF) [file pone.0285645.s003.pdf]

MASTER STATISTICAL ANALYSIS PLAN (M-SAP)

**Title**	NIH RECOVER: A Multi-site Pathology Study of Post-Acute Sequelae of SARS-CoV-2 Infection
**Short Title**	NIH RECOVER Tissue Pathology: Understanding the Long-Term Impact of COVID
**Sponsor**	National Institutes of Health
**Current Protocol Version**	2.0
**Current Protocol Date**	07 FEB 2022
**Statistical Analysis Plan Version**	1.1
**Statistical Analysis Plan Date**	AUG 2022

M-SAP APPROVAL SIGNATURES

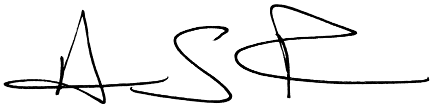

10/31/2022

Date

Principal Investigator, RECOVER Data Resource Core

SAP REVISION HISTORY

Version	Date	Description of Changes
1.0	JUNE 2022	Initial version
1.1	OCT 2022	Revised version based on NIH feedback

TABLE CONTENTS

1 INTRODUCTION 7

1.1 STUDY OBJECTIVES 7

1.2 OBJECTIVES OF THE STATISTICAL ANALYSIS PLAN 8

2 STUDY DESIGN 8

2.1 OVERALL STUDY DESIGN 8

2.2 STUDY POPULATION 8

2.2.1 Decedents with PASC 9

2.2.2 Decedents without PASC 10

2.2.3 Stratified recruitment 10

2.3 SAMPLE SIZE DETERMINATIONS 10

2.4 INTERIM ANALYSES AND POTENTIAL STUDY MODIFICATIONS 12

2.5 DATA COLLECTION 12

2.5.1 Autopsy 12

2.5.2 Submission of biospecimens to the RECOVER Biospecimen Core (PBC) 12

2.5.3 Post-mortem MRI studies of CNS 13

2.5.4 Clinical Data 13

2.5.5 Primary endpoints 13

3 STATISTICAL METHODS 14

3.1 GENERAL STATISTICAL METHODS AND DATA HANDLING 14

3.1.1 Alignment of data across sources 14

3.1.2 Data quality control procedures 14

3.1.3 Summary statistics and data visualizations 14

3.1.4 Possible transformation of outcome variables 15

3.1.5 Overlap assessment 15

3.1.6 Covariate balance assessment and matched-sampling strategy 15

3.1.7 Fisherian inference (e.g., hypothesis testing) 15

3.1.8 Model-based inference (e.g., estimated point estimates, confidence intervals) 16

3.1.9 Statistical software 16

3.2 CHARACTERISTICS OF DECEDENTS 16

3.2.1 Demographic comparisons 16

3.2.2 Comorbidities comparisons 18

3.2.3 Comparisons of clinical characteristics 19

3.2.4 Laboratory comparisons 20

3.3 ANALYTIC APPROACH TO AIM 1 21
3.3.1 Scientific hypotheses related to Aim 1 21
3.3.2 Overview of analytic approach to Aim 1 22
3.3.3 Overlap assessment 22
3.3.4 Covariate balance and matched-sampling strategies 22
3.3.5 Fisherian inference 22
3.3.6 Model-based inference 23
3.4 ANALYTIC APPROACH TO AIM 2 23
3.4.1 Scientific hypotheses related to Aim 2 23
3.4.2 Overview 23
3.4.3 Overlap assessment 24
3.4.4 Covariate balance and matched-sampling 24
3.4.5 Fisherian inference 24
3.4.6 Model-based inference 24
3.5 ANALYTIC APPROACH TO AIM 3 25
3.5.1 Scientific hypotheses related to Aim 3 25
3.5.2 Overview 25
3.5.3 Overlap assessment 25
3.5.4 Covariate balance and matched-sampling 25
3.5.5 Fisherian inference 25
3.5.6 Model-based inference 26
3.6 ANALYTIC APPROACH TO AIM 4 26
3.6.1 Scientific hypotheses related to Aim 4 26
3.6.2 Overview 26
3.6.3 Overlap assessment 26
3.6.4 Covariate balance and matched-sampling 26
3.6.5 Fisherian inference 26
3.6.6 Model-based inference 27
3.7 METHODS FOR MISSING DATA 27
3.8 COMMUNICATIONS 27
3.9 OBSERVATIONAL STUDY MONITORING BOARD 27
4 REFERENCES 29

ABBREVIATIONS

Abbreviation	Definition
ALT	Alanine Transaminase
AST	Aspartate Transaminase
BMI	Body Mass Index
BUN	Blood Urea Nitrogen
CAP	College of American Pathologists
CDEs	Common Data Elements
CI	Confidence Interval
CNS	Central Nervous System
CRF	Case Report Form
CSC	Clinical Sciences Core
ddPCR	droplet digital Polymerase Chain Reaction
DRC	Data Resource Core
EDC	Electronic Data Capture
EHR	Electronic Health Record
FAD	Final Anatomic Diagnosis
FI	Fisherian Interval
HIPAA	Health Insurance Portability and Accountability Act
HHS	Health and Human Services
IQR	Interquartile Range
ISH	In Situ Hybridization
MICE	Multiple Imputation by Chained Equations
MOP	Manual of Procedures
MRI	Magnetic Resonance Imaging
NBF	Neutral Buffered Formalin

NHLBI	National Heart, Lung, and Blood Institute
NSIP	Non-Specific Interstitial Pneumonia
OP	Organized Pneumonia
OSMB	Observational Study Monitoring Plan
PASC	Post-Acute Sequelae of SARS-CoV-2 infection
REDCap	Research Electronic Data Capture
SAP	Statistical Analysis Plan
SNOMED	Systemized Nomenclature of Medicine
UIP	Usual Interstitial Pneumonia
WSI	Whole Slide Imaging

1 Introduction

1.1 Study objectives

The goal of the NIH RECOVER Tissue Pathology Study is to define and characterize the epidemiology, natural history, clinical spectrum, and underlying mechanisms of post-acute sequelae of SARS-CoV-2 infection (PASC) in a diverse population representative of the general COVID-19 population in the US.

This cross-sectional study will characterize the pathology of PASC in non-hospitalized and hospitalized patients who die 30 days or later from the time of SARS-CoV-2 infection (the first SARS-CoV-2 infection, if there is evidence of multiple infections). Also note that hospitalization status for COVID-19 is defined by whether a decedent was ever hospitalized for COVID-19 within 30 days of a SARS-CoV-2 infection. The study will include decedents who meet clinical criteria of PASC as defined by the recent World Health Organization publication (see our working definition of PASC in Section 2.2.1), and decedents who had previously recovered from SARS-CoV-2 infection (i.e., death occurred more than 30 days after SARS-CoV-2 infection). We will examine whether PASC status, pre-COVID medical conditions, and demographic and biological risk factors are associated with gross and microscopic findings at autopsy.

The Tissue Pathology Study will also explore the pathology of acute SARS-CoV-2 infection in a smaller subset of non-hospitalized and hospitalized patients who died 15-30 days from their SARS-CoV-2 infection (the first SARS-CoV-2 infection, if there is evidence of multiple infections). Again, note that hospitalization status for COVID-19 is defined by whether a decedent was ever hospitalized for COVID-19 within 30 days of a SARS-CoV-2 infection.

<u>Aim 1</u>: Characterize the prevalence and types of organ injury/disease, pathological features, and distinct phenotypes in decedents who died during the acute phase of SARS-CoV-2 infection (dying 15-30 days after first SARS-CoV-2 infection), and in decedents who died during the post-acute phase of SARS-CoV-2 infection (dying more than 30 days after first SARS-CoV-2 infection) with and without PASC.

<u>Aim 2</u>: Characterize the association of clinical measures of COVID-19 disease severity and associated risk factors with pathological findings in decedents with prior SARS-CoV-2 infection (dying more than 30 days after SARS-CoV-2 infection) with and without PASC.

- 2a. Characterize the association of patterns of COVID-19 disease severity and treatment with pathological findings in decedents with prior SARS-CoV-2 infection with and without PASC over time.
- 2b. Determine whether pre-infection and peri-infection risk and resiliency factors (e.g., demographic, biological factors, pre-existing clinical comorbidities) are associated with pathological findings in decedents with prior SARS-CoV-2 infection with and without PASC.

<u>Aim 3</u>: Define the pathophysiology of and mechanisms associated with pathological findings, including the direct and indirect causal effects of clinical risk factors (e.g., comorbidities, infection severity, hospitalization, intubation, steroid treatment) on pathological findings via viral persistence (investigated systematically via molecular methods), in post-acute decedents with and without PASC.

<u>Aim 4</u>: Establish a multi-center post-mortem tissue biobank and a post-mortem brain magnetic resonance imaging (MRI) bank from decedents with prior SARS-CoV-2 infection with and without PASC. The primary goal of the brain imaging is to find and characterize lesions in the central nervous system (CNS) via image-directed sampling of identified lesions. A secondary goal is to evaluate a small subset of decedents with pre-infection and post-mortem brain MRIs to examine whether decedent's PASC status is associated with the presence of new lesion(s) in the CNS.

1.2 Objectives of the Statistical Analysis Plan

This statistical analysis plan (SAP) outlines the statistical methods to be used in the primary analyses of RECOVER Pathology Study of PASC data to address the study objective(s) indicated in the protocol. Populations for analysis, data handling rules, and statistical methods are provided. The statistical analyses and summary tabulations described in this SAP will provide the basis for the results sections of associated manuscripts, reports, and presentations. We plan to submit the SAP to *ClinicalTrials.gov*.

2 Study design

2.1 Overall study design

The decedents will enter this cross-sectional Tissue Pathology Study at varying stages after their infection with SARS-CoV-2 (see Figure 1). This study will be conducted in the United States and decedents will be enrolled through interaction with families of the deceased at inpatient, outpatient, and community-based settings.

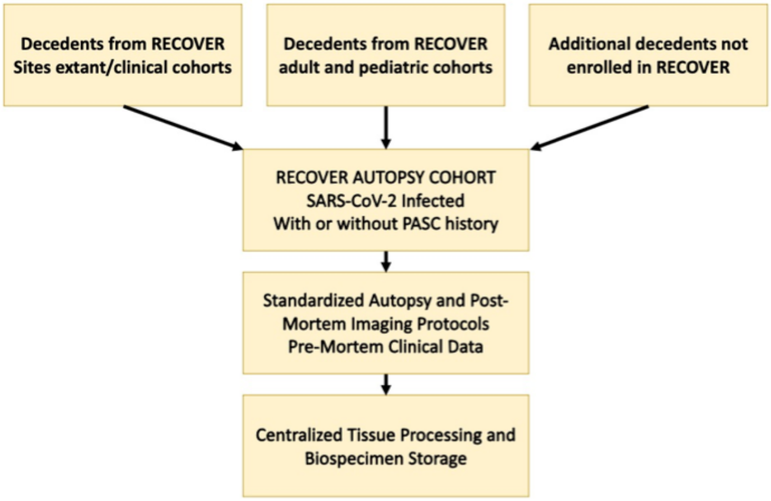

Figure 1: Stages of the Tissue Pathology Study

The Tissue Pathology Study will: (i) characterize the pathology of PASC in hospitalized and non-hospitalized patients who died 30 days or later from their SARS-CoV-2 infection, and (ii) explore the pathology of acute SARS-CoV-2 infection in a smaller subset of patients who died 15-30 days from their SARS-CoV-2 infection.

Recall that we will consider the first SARS-CoV-2 infection, if there is some evidence of multiple infections, and hospitalization status for COVID-19 is defined by whether a decedent was ever hospitalized for COVID-19 within 30 days of a SARS-CoV-2 infection.

Study data, including age, sex, race / ethnicity, medical history, vaccination history, details of acute SARS-CoV-2 infection, overall health and physical function, and PASC symptom screen, will be collected from the electronic health record (EHR) using a case report form (CRF).

2.2 Study population

The RECOVER Tissue Pathology Study population includes four categories of decedents:

1. <u>Post-acute decedents</u> (death occurred 30 days or later after SARS-CoV-2 infection):
   a. Non-hospitalized patients,

   b. Hospitalized patients,
2. <u>Acute decedents</u> (death occurred 15-30 days after SARS-CoV-2 infection):
   a. Non-hospitalized patients,
   b. Hospitalized patients.

Additional criteria for decedent enrollment are included below.

2.2.1 Decedents with PASC

Decedents meeting WHO criteria for suspected, probable, or confirmed SARS-CoV-2 infection, who die more than 30 days after SARS-CoV-2 infection, and decedents who site investigators believe meet the working definition of PASC, will be recruited. Note that the definition of PASC given in this Section may evolve over the time of the Tissue Pathology Study.

The diagnosis of PASC will be based on having one of three primary symptoms:

- fatigue (i.e., being very tired),
- shortness of breath,
- problems thinking or concentrating (i.e., "brain fog"),

or at least two secondary symptoms:

- post-exertional malaise (symptoms worse after even minor physical or mental effort),
- weakness in arms or legs,
- fever, chills, sweats or flushing,
- loss of or change in smell or taste,
- pain in any part of body,
- persistent (chronic) cough,
- palpitations, racing heart, arrhythmia, skipped beats,
- gastrointestinal (belly) symptoms (feeling full or vomiting after eating, diarrhea, constipation),
- nerve problems (tremor, shaking, abnormal movements, numbness, tingling, burning, cannot move part of body, new seizures),
- problems with anxiety, depression, stress, or trauma-related symptoms like nightmares or grief,
- problems with sleep,
- feeling faint, dizzy, "goofy"; difficulty thinking soon after standing up from a sitting or lying position,
- vision problems (blurry, light sensitivity, difficulty reading or focusing, floaters, flashing lights, "snow"),
- problems with hearing (hearing loss, ringing in ears),
- changes to menstrual cycle,

during the post-acute phase of COVID-19, but not prior to SARS-CoV-2 infection.

Note that symptoms occurring during the acute phase of COVID-19 or at an unknown time will not be considered for PASC diagnosis. Also note that, as the definition of PASC evolves in light of information obtained from the RECOVER Adult Cohort Study, reviewed at quarterly intervals, we will apply the adjusted definitions to decedents.

2.2.2 Decedents without PASC

Infected acute and post-acute decedents must meet the following conditions at the time of RECOVER autopsy enrollment:

1. Acute decedents: Individuals meeting WHO criteria for suspected, probable, or confirmed SARS-CoV-2 infection who die 15-30 days after their SARS-CoV-2 infection. Note that acute decedents die before developing PASC.
2. Post-acute decedents: Individuals meeting WHO criteria for suspected, probable, or confirmed SARS-CoV-2 infection who die without symptoms of PASC more than 30 days after their SARS-CoV-2 infection. Note that the absence of PASC symptoms will be based on the contemporaneous working definition of PASC, which may evolve over time.

2.2.3 Stratified recruitment

Recruitment will occur across sites (see the Tissue Pathology Study Protocol for more details). Recruitment of SARS-CoV-2 infected decedents will be stratified to ensure:

- 50% of decedents will be female,
- 53% of decedents will be non-Hispanic Whites, 16% non-Hispanic Blacks, 27% Hispanic/Latinx, and 4% Asian Americans, Native Hawaiians, Pacific Islanders, American Indians, and Native Alaskan,
- 57% of decedents will be post-acute decedents with PASC, 29% post-acute decedents without PASC, and 14% acute decedents.

Note that the autopsy sites will frequently communicate with RECOVER Clinical Sciences Core (CSC) and Data Resource Core (DRC) to ensure that the targeted recruitment is satisfied, and that the groups of decedents with and without PASC are balanced with respect to background covariates (e.g., age, sex, race / ethnicity). For example, it is anticipated that post-acute decedents with PASC may be older than the ones without PASC.

2.3 Sample size determinations

N=700 individuals will be recruited to the RECOVER Tissue Pathology Study, including N=400 post-acute decedents with PASC, N=200 post-acute decedents without PASC, and N=100 acute decedents (**Table 1**).

Table 1. Sample size for the RECOVER Tissue Pathology Study by PASC status

	PASC status		Total
	Without PASC	With PASC	
Post-acute decedents (dying > 30 days after SARS-CoV-2 infection)	200	400	600
Acute decedents (dying 15-30 days from SARS-CoV-2 infection)	100		100
Total	300	400	700

Aim 1: Characterize the prevalence and types of organ injury/disease, pathological features, and distinct phenotypes in decedents with acute SARS-CoV-2 infection (dying 15-30 days after SARS-CoV-2 infection), and in decedents with prior SARS-CoV-2 infection (dying >30 days after SARS-CoV-2 infection) with and without PASC.

Table 2 provides detectable effect sizes and detectable differences in proportions between decedents with and without PASC for quantitative and dichotomous pathological features. Results are given without and with a multiple comparison adjustment (assuming 50 tests, alpha=0.001 as a conservative level of control).

Table 2. Detectable effect measures

Total sample size (not including acute decedents)	Number of post-acute decedents with PASC	Number of post-acute decedents without PASC	***Detectable standardized effect size*** for 80% power, alpha=0.05, 0.001 (i.e., without and with multiple comparison adjustment), based on two independent group, two-sided comparison of means	***Detectable difference in proportion*** between groups of post-acute decedents with and without PASC with 80% power, alpha=0.05, 0.001 (i.e., without and with multiple comparison adjustment), based on two independent groups, two-sided comparison of proportions	
				Proportion with feature = 0.2 in PASC- individuals	Proportion with feature = 0.4 in PASC- individuals
300	150	150	0.32, 0.48	0.14, 0.22	0.16, 0.24
300	200	100	0.34, 0.51	0.15, 0.24	0.17, 0.25
400	200	200	0.28, 0.42	0.13, 0.19	0.14, 0.21
400	267	133	0.30, 0.44	0.13, 0.20	0.15, 0.22
600	300	300	0.23, 0.34	0.10, 0.15	0.12, 0.17
600	400	200	0.24, 0.36	0.11, 0.16	0.12, 0.18
700	350	350	0.21, 0.31	0.09, 0.14	0.11, 0.16
700	467	233	0.23, 0.33	0.10, 0.15	0.11, 0.17
800	400	400	0.20, 0.29	0.09, 0.13	0.10, 0.15
800	534	266	0.21, 0.31	0.09, 0.14	0.11, 0.16
900	450	450	0.19, 0.28	0.08, 0.12	0.10, 0.14
900	600	300	0.20, 0.29	0.09, 0.13	0.10, 0.15

<u>Aim 2:</u> Characterize the association of clinical measures of COVID-19 disease severity and associated risk factors with pathological findings in decedents with prior SARS-CoV-2 infection (dying > 30 days after SARS-CoV-2 infection) with and without PASC.

A sample size of 400 within one group (e.g., post-acute decedent with PASC) will have greater than 80% power to detect a small effect size, i.e., Cohen's d=0.28 between individuals with and without the risk factor, assuming 50% of individuals have the risk factor (i.e., 200 with and 200 without the risk factor; row 3 in Table 2). To characterize the detectable effect sizes from the subset of decedents who

undergo brain imaging, for example, please see the first row of Table 2. A sample size of 300 provides 80% power to detect a moderate effect size of d=0.32 between individuals with and without the risk factor (within the specified group), assuming 50% of individuals have the risk factor. These calculations are based on a two-group, two-sided comparison of means, and controlling type 1 error at 0.05.

2.4 Interim analyses and potential study modifications

The Tissue Pathology Study Protocol is designed to be pragmatic and flexible in design. We will undertake the following procedures to guide protocol modifications over time in consultation with the Observational Study Monitoring Board (OSMB):

1. Data elements that are not NIH recommended Common Data Elements (CDEs) may be modified based on ongoing analysis by DRC.
2. PASC definition will be revised in an iterative manner based on consortium investigator expertise, existing PASC data, medical literature, and feedback from family representatives, and the scientific community. Updated PASC definitions may be used to implement a strategy to modify deeper phenotyping.

2.5 Data Collection

2.5.1 Autopsy

Autopsies must be performed within 24 hours of death. Procedures must comply with those set forth in the Tissue Pathology Study Protocol (Appendix 1 - Gross Autopsy Template) and the Manual of Procedures (MOP). These are summarized here.

1. Prior to conducting the autopsy, collect blood from decedent for serum separator tubes and blood cards. Allow the blood cards to dry completely prior to closing and room temperature storage. Cerebrospinal fluid (CSF) will be obtained via a cisternal tap and frozen.
2. Apply the appropriate modified College of American Pathologists (CAP) Gross Autopsy protocol (adult), including both gross and microscopic examination, and collect the autopsy common data elements (demographic, external, and internal findings). These data will include structured fields for the gross and certain microscopic findings. The microscopic reports will also be captured as text fields, and manually coded into approved terms and systemized nomenclature of medicine (SNOMED) by pathology coders; these codes will be finalized following review by the responsible pathologist.
3. Generate the final anatomic diagnosis (FAD) from a standardized list of approved terms,
4. Collect fresh tissue from 50 anatomic sites representing many organ systems as dictated by the Autopsy Gross Template (see Tissue Pathology Study Protocol, Appendix 1), to be snap frozen (two 1cm cubes) and paraffin embedded (one 1cm cube). Two matched tissue samples from the Autopsy Template should be placed in a tissue cassette, with additional sample types as necessary for viral quantification. From the visceral organs 40 or more sites should be sampled, and from the central nervous system (CNS) 15 or more sites should be sampled, including cerebrospinal fluid (CSF).

Specimens in tissue cassettes will be fixed in neutral buffered formalin (NBF) for approximately 6 hours not to exceed 48 hours.

2.5.2 Submission of biospecimens to the RECOVER Biospecimen Core (PBC)

A centralized biorepository has been selected to process and store biospecimens for this study. All autopsy study sites will collect and prepare biospecimens, including blood, body fluids, snap frozen and

formalin-fixed tissue samples, as per the protocol, for shipping to the centralized RECOVER biorepository, where all specimens will be processed and stored. In situ hybridization (ISH), droplet digital polymerase chain reaction (ddPCR), and whole slide imaging (WSI) will be performed centrally.

2.5.3 Post-mortem MRI studies of CNS

It is anticipated that 50% or more of decedents will be subject to examination of the CNS by autopsy, and will be evaluated with post-mortem brain MRI.

2.5.4 Clinical Data

Abstracted decedent medical records will be linked, when possible, to RECOVER cohort and EHR information. The following elements of clinical data will be obtained from review of the decedent's EHR and available data collected from enrollment and follow-up in the RECOVER adult and pediatric cohort studies, if applicable:

1. Electronic Health Records (EHR) SARS-CoV-2 vaccination status,
2. RECOVER study questionnaires, if applicable,
3. Physical examinations,
4. Biomarkers,
5. Biospecimens,
6. Imaging.

See more details in the Tissue Pathology Study Protocol.

2.5.5 Primary endpoints

Our statistical analyses will examine the following primary pathological outcomes:

- Lung
    - fibrosis
    - inflammation
    - thrombosis
    - presence of SARS-CoV-2
- Cardiovascular system
    - fibrosis
    - non-ischemic inflammation
    - acute ischemic injury
    - infarct
    - atherosclerosis / arteriosclerosis
    - thrombosis
    - presence of SARS-CoV-2
- Liver
    - fibrosis
    - inflammation
    - thrombosis
    - presence of SARS-CoV-2
- Kidney
    - fibrosis
    - inflammation

- thrombosis
- presence of SARS-CoV-2

- Lymph node / spleen
  - Hemophagocytosis
  - reactive lymph nodes
  - presence of SARS-CoV-2
- Brain
  - inflammation
  - infarct
  - thrombosis
  - hemorrhages (non-traumatic)
  - atherosclerosis / arteriosclerosis
  - vasculitis
  - vascular malformation / aneurysm
  - neurodegenerative disease
  - presence of SARS-CoV-2.

3 Statistical methods

3.1 General statistical methods and data handling

3.1.1 Alignment of data across sources

The majority of the study data (i.e., gross and microscopic findings) will be collected at autopsy according to the standardized Tissue Pathology Study Protocol. Recall that the gross and certain microscopic findings will be entered in the Research Electronic Data Capture (REDCap) system as structured fields, and that the microscopic reports as text field, which will later be systematically coded into SNOMED codes. Additional EHR and other research data (e.g., viral persistence, post-mortem brain imaging) may be extracted, entered into electronic case report forms, and aligned across sites.

3.1.2 Data quality control procedures

Rigorous quality control procedures will be applied, including remote and continuous monitoring using field- and form-level validation at the time of data entry, regular backend monitoring of data with clinical oversight, and training and assistance of lead sites in monitoring of their sub-sites. Network-wide accrual and compliance reports will be provided to investigators to encourage quality of data. Data quality and logic checks will be built into every step of data acquisition, capture, transfer, and integration to assure scientific rigor and to minimize potential errors. Whenever possible, automated logic checks will be built into REDCap.

3.1.3 Summary statistics and data visualizations

The analysis pipeline will include calculating basic summary statistics (e.g., means, SDs, percentiles, counts, proportions), cross-tabulating data (e.g., contingency tables), and performing basic data visualization (e.g., histograms, box plots, scatter plots). Additional visualization tools will be applied for deeper data exploration, which will include results from software packages *ggplot2*, *highcharter*, *RGL* available in R, and PhenoTree (Baytas et al., 2016).

Prior to fitting Frequentist and Bayesian statistical models, thorough exploratory data analysis will be performed to identify outliers, determine reasonable modeling assumptions, and refine existing and

formulate new hypotheses and analyses based on any trends and patterns identified. We will make use of standard visual diagnostics (e.g., residual plots, quantile-quantile plots) and/or formal statistical tests (e.g., likelihood ratio test, goodness-of-fit test) to evaluate the adequacy of fitted regression models.

3.1.4 Possible transformation of outcome variables

Outcome variables may be transformed (e.g., Box-Cox power family – Box and Cox, 1964) prior to any statistical modeling. Bayesian models will be used to provide simulation-based inference for transformed outcomes (Rubin, 1978). Original, untransformed values will be used for all summaries.

3.1.5 Overlap assessment

We will assess the overlap in covariate distributions between comparative groups (i.e., acute decedents vs. post-acute decedents without PASC) using the empirical distributions of the estimated propensity scores (e.g., Stuart, 2010; Bind and Rubin, 2019). We will remove “outlier” decedents whose estimated propensity scores do not overlap with the other group of decedents (e.g., Imbens and Rubin, 2015; Bind and Rubin, 2019).

3.1.6 Covariate balance assessment and matched-sampling strategy

Using standard diagnostics (e.g., “Love” plots – Ahmed et al., 2006), we will assess balance in background covariates (e.g., sex, age, race/ethnicity, hospitalization status): (i) between groups of acute decedents and of post-acute decedents without PASC, and (ii) between groups of post-acute decedents with and without PASC. If there is some evidence of covariate imbalance, we will use matched-sampling techniques (e.g., estimated propensity score matching – Rosenbaum and Rubin, 1983) to improve balance (e.g., Stuart, 2010). Note that some decedents’ data may be discarded at this stage. Also note that this design stage task will be performed blinded from the outcome data, by a biostatistician who will not be involved in the statistical analysis stage.

3.1.7 Fisherian inference (e.g., hypothesis testing)

We will test sharp null hypotheses and report unadjusted and adjusted (for multiple comparison - Lee et al., 2016) two-sided Fisher p-values. For example, in Aim 1, for the comparison of acute decedents and post-acute decedents without PASC, a sharp null hypothesis will state that for each decedent, the pathological potential outcomes are the same had the decedent been an acute decedent or a post-acute decedent without PASC. For the comparison of post-acute decedents with and without PASC, a sharp null hypothesis will state that for each post-acute decedent, the pathological potential outcomes are the same, whether the decedent had PASC or not. For Aim 1, the pathological outcomes include the presence and types of organ injury / disease, pathological features, and distinct phenotypes. We will calculate Fisher p-values based on the assumed, hypothetical exposure assignment mechanism implied by the data and the matched-sampling strategy (Bind and Rubin, 2019; Branson, 2021). For test statistics, we will use standard ones (e.g., proportion difference), and less commonly-used ones (e.g., estimated coefficient from a multivariable regression model – Brillinger et al., 1978; Bind and Rubin, 2019; Sommer et al., 2021). Following the advice of Wasserstein et al. (2019), we will “not base our conclusions solely on whether an association or effect was found to be ‘statistically significant’ (i.e., the p-value passed some arbitrary threshold such as $p < 0.05$)”, but rather also explore Fisherian intervals - FIs for unit-level difference in continuous potential outcomes (e.g., Imbens and Rubin, 2015) and for finite sample proportion difference (Volfovsky et al., 2015), and consider model-based inference.

3.1.8 Model-based inference (e.g., estimated point estimates, confidence intervals)

For the comparative (e.g., two-sample) analyses described in the previous section, we will also provide model-based inference (e.g., Neymanian / Frequentist, Bayesian). For continuous variables, we will assess non-linear dose-responses with penalized cubic splines (e.g., *mgcv* package in R – Wood and Wood, 2015). Robustness of study results to alternative model specifications will be assessed in *ad hoc* sensitivity analyses. We will define an estimand of interest and provide a point estimate and a corresponding two-sided uncertainty interval (e.g., 95% confidence interval – CI) for that estimand. We will use standard large-sample approximations for estimated variances of estimated effects (e.g., estimated difference in proportions), and also capitalize on Frequentist and Bayesian multivariable regression models implemented in R.

3.1.9 Statistical software

Statistical analyses will be performed using R version 4.1.1.

3.2 Characteristics of decedents

Summary statistics, including sample sizes, medians and interquartile ranges for numeric variables, and counts and proportions for categorical variables, will be reported for data of decedents including demographics, comorbidities, vaccination, clinical assessments, and laboratory values (**Tables 3-6**). We will also categorize decedents according to calendar quarter of enrollment, as well as calendar quarter of initial infection. This will allow assessment of differences in pathologic features that may occur following infection with different SARS-CoV-2 variants, in addition to the time elapsed between initial infection and death. Summaries will be reported overall and stratified by PASC status, decedent status (acute vs. post-acute), hospitalization history (hospitalized vs. not hospitalized for COVID-19), and time of enrollment. P-values corresponding to univariate (unadjusted) two-sample tests of differences in means or proportions: (i) between the groups of acute decedents and of post-acute decedents without PASC, and (ii) between the groups of post-acute decedents with and without symptoms of PASC.

3.2.1 Demographic comparisons

3.2.1.1 Demographic comparisons between acute decedents and post-acute decedents without PASC

Table 3a. Demographic characteristics for decedents without PASC overall and by acute vs. post-acute status

	Overall (N=)	Acute decedents (death 15-30 days after SARS-CoV-2 infection) (N=)	Post-acute decedents without PASC (death >30 days after SARS-CoV-2 infection) (N=)	Acute decedents vs. post-acute decedents without PASC
**Demographics**				
Female sex – count/total (%)	count/total (%)	count/total (%)	count/total (%)	*p-value*
Age – Median (interquartile range - IQR)	Median (IQR)	Median (IQR)	Median (IQR)	*p-value*
Age 18-34 years	count/total (%)	count/total (%)	count/total (%)	
Age 35-49 years	count/total (%)	count/total (%)	count/total (%)	
Age 50-64 years	count/total (%)	count/total (%)	count/total (%)	
Age ≥*65 years*	count/total (%)	count/total (%)	count/total (%)	
Race/Ethnicity				

White/non-Hispanic	count/total (%)	count/total (%)	count/total (%)	
Black/non-Hispanic	count/total (%)	count/total (%)	count/total (%)	
Hispanic	count/total (%)	count/total (%)	count/total (%)	
Asian	count/total (%)	count/total (%)	count/total (%)	*p-value*
Native Hawaiian/Pacific Islander	count/total (%)	count/total (%)	count/total (%)	
American Indian/Alaska Native	count/total (%)	count/total (%)	count/total (%)	
Other	count/total (%)	count/total (%)	count/total (%)	
Geographic region*				
HHS Region 1	count/total (%)	count/total (%)	count/total (%)	
HHS Region 2	count/total (%)	count/total (%)	count/total (%)	
HHS Region 3	count/total (%)	count/total (%)	count/total (%)	
HHS Region 4	count/total (%)	count/total (%)	count/total (%)	
HHS Region 5	count/total (%)	count/total (%)	count/total (%)	
HHS Region 6	count/total (%)	count/total (%)	count/total (%)	*p-value*
HHS Region 7	count/total (%)	count/total (%)	count/total (%)	
HHS Region 8	count/total (%)	count/total (%)	count/total (%)	
HHS Region 9	count/total (%)	count/total (%)	count/total (%)	
HHS Region 10	count/total (%)	count/total (%)	count/total (%)	
Rural	count/total (%)	count/total (%)	count/total (%)	*p-value*

* https://www.hhs.gov/about/agencies/iea/regional-offices/index.html

3.2.1.2 Demographic comparisons between post-acute decedents with and without PASC

Table 3b. Demographic characteristics for post-acute decedents overall and by PASC status

	Overall (N=)	Post-acute decedents without PASC (N=)	Post-acute decedents with PASC (N=)	Post-acute decedents without vs. with PASC
**Demographics**				
Female sex – count/total (%)	count/total (%)	count/total (%)	count/total (%)	*p-value*
Age – Median (IQR)	Median (IQR)	Median (IQR)	Median (IQR)	
Age 18-34 years	count/total (%)	count/total (%)	count/total (%)	
Age 35-49 years	count/total (%)	count/total (%)	count/total (%)	*p-value*
Age 50-64 years	count/total (%)	count/total (%)	count/total (%)	
Age ≥*65 years*	count/total (%)	count/total (%)	count/total (%)	
Race/Ethnicity				
White/non-Hispanic	count/total (%)	count/total (%)	count/total (%)	
Black/non-Hispanic	count/total (%)	count/total (%)	count/total (%)	
Hispanic	count/total (%)	count/total (%)	count/total (%)	
Asian	count/total (%)	count/total (%)	count/total (%)	*p-value*
Native Hawaiian/Pacific Islander	count/total (%)	count/total (%)	count/total (%)	
American Indian/Alaska Native	count/total (%)	count/total (%)	count/total (%)	

Other	count/total (%)	count/total (%)	count/total (%)	
Geographic region*				
HHS Region 1	count/total (%)	count/total (%)	count/total (%)	p-value
HHS Region 2	count/total (%)	count/total (%)	count/total (%)	
HHS Region 3	count/total (%)	count/total (%)	count/total (%)	
HHS Region 4	count/total (%)	count/total (%)	count/total (%)	
HHS Region 5	count/total (%)	count/total (%)	count/total (%)	
HHS Region 6	count/total (%)	count/total (%)	count/total (%)	
HHS Region 7	count/total (%)	count/total (%)	count/total (%)	
HHS Region 8	count/total (%)	count/total (%)	count/total (%)	
HHS Region 9	count/total (%)	count/total (%)	count/total (%)	
HHS Region 10	count/total (%)	count/total (%)	count/total (%)	
Rural	count/total (%)	count/total (%)	count/total (%)	*p-value*

* https://www.hhs.gov/about/agencies/iea/regional-offices/index.html

3.2.2 Comorbidities comparisons

3.2.2.1 Comorbidities comparisons between acute decedents and post-acute decedents without PASC

Table 4a. Comorbidities for decedents without PASC overall and by acute vs. post-acute status

	Overall (N=)	Acute decedents (N=)	Post-acute decedents without PASC (N=)	Acute decedents vs. Post-acute decedents without PASC
**Comorbidities (prior to infection date)**				
See Table 5	count/total (%)	count/total (%)	count/total (%)	*p-value*

3.2.2.2 Comorbidities comparisons between post-acute decedents with and without PASC

Table 4b. Comorbidities for post-acute decedents overall and by PASC status

	Overall (N=)	Post-acute decedents without PASC (N=)	Post-acute decedents with PASC (N=)	Post-acute decedents without vs. with PASC
**Comorbidities (prior to infection date)**				
See Table 5	count/total (%)	count/total (%)	count/total (%)	*p-value*

Table 5. Comorbidities

1. Immunocompromised condition
2. Rheumatologic, autoimmune or connective tissue disease

11. Asthma
12. Chronic obstructive pulmonary disease including emphysema,

21. Myalgic encephalomyelitis / chronic fatigue syndrome

3. Current cancer or ongoing cancer treatment
4. Chronic liver disease
5. Obesity
6. Diabetes
7. Kidney disease
8. High blood pressure
9. Cardiovascular disease (e.g., heart failure, heart attack)
10. Stroke, transient ischemic attach or mini-stroke), intracerebral hemorrhage or subarachnoid hemorrhage, or cerebral venous thrombosis

chronic bronchitis, obstructive pulmonary disease

13. Other chronic lung disease
14. Use of oxygen at home
15. Sickle cell anemia
16. Dementia, memory impairment, cognitive disorder, or developmental delay
17. Depression or anxiety disorder
18. Bipolar disorder or psychosis
19. Other mental health disorder
20. Chronic pain syndrome or fibromyalgia

22. Postural orthostatic tachycardia syndrome or other form of dysautonomia or autonomic dysfunction
23. Polycystic ovarian syndrome
24. Central nervous system infection, inflammatory disease or demyelinating disease
25. Seizure disorder
26. Neuromuscular disease (e.g., neuropathy, myopathy, myasthenia gravis)
27. Movement disorder (e.g., Parkinsonism, essential tremor)
28. Transplant

3.2.3 Comparisons of clinical characteristics

3.2.3.1 Clinical characteristics comparisons between acute decedents and post-acute decedents without PASC

Table 6a. Clinical characteristics for decedents without PASC overall and by acute vs. post-acute status

	Overall (N=)	Acute decedents (N=)	Post-acute decedents without PASC (N=)	Acute decedents vs. Post-acute decedents without PASC
**Vaccination**				
Complete vaccine series	count/total (%)	count/total (%)	count/total (%)	*p-value*
Incomplete vaccine series	count/total (%)	count/total (%)	count/total (%)	*p-value*
Vaccine booster received	count/total (%)	count/total (%)	count/total (%)	*p-value*
**Clinical Assessments**				
Pregnancy status during acute infection phase	count/total (%)	count/total (%)	count/total (%)	*p-value*
Hospitalization status	count/total (%)	count/total (%)	count/total (%)	*p-value*
Body mass index (BMI) (latest record prior SARS-CoV-2 infection) – Median (IQR)	Median (IQR)	Median (IQR)	Median (IQR)	*p-value*
<18.5 kg/m^2	count/total (%)	count/total (%)	count/total (%)	*p-value*
18.5-24.9 kg/m^2	count/total (%)	count/total (%)	count/total (%)	
25-29.9 kg/m^2	count/total (%)	count/total (%)	count/total (%)	
≥*30* kg/m^2	count/total (%)	count/total (%)	count/total (%)	

3.2.3.2 Clinical characteristics comparisons between post-acute decedents with and without PASC

Table 6b. Clinical characteristics for post-acute decedents overall and by PASC status

	Overall (N=)	Post-acute decedents without PASC (N=)	Post-acute decedents with PASC (N=)	Post-acute decedents without vs. with PASC
**Vaccination**				
Complete vaccine series	count/total (%)	count/total (%)	count/total (%)	*p-value*
Incomplete vaccine series	count/total (%)	count/total (%)	count/total (%)	*p-value*
Vaccine booster received	count/total (%)	count/total (%)	count/total (%)	*p-value*
**Clinical Assessments (latest records)**				
Pregnancy during acute infection phase	count/total (%)	count/total (%)	count/total (%)	*p-value*
Hospitalization status	count/total (%)	count/total (%)	count/total (%)	*p-value*
BMI (latest record prior SARS-CoV-2 infection)– Median (IQR)	Median (IQR)	Median (IQR)	Median (IQR)	*p-value*
<18.5 kg/m^2	count/total (%)	count/total (%)	count/total (%)	*p-value*
18.5-24.9 kg/m^2	count/total (%)	count/total (%)	count/total (%)	
25-29.9 kg/m^2	count/total (%)	count/total (%)	count/total (%)	
≥*30* kg/m^2	count/total (%)	count/total (%)	count/total (%)	

3.2.4 Laboratory comparisons

3.2.4.1 Laboratory comparisons between acute decedents and post-acute decedents without PASC

Table 7a. Laboratory values for decedents without PASC overall and by acute vs. post-acute status

**Laboratory Assessments (latest records prior to SARS-CoV-2 infection)**	Overall (N=)	Acute decedents (N=)	Post-acute decedents without PASC (N=)	Acute decedents vs. Post-acute decedents without PASC
White blood cell count	Median (IQR)	Median (IQR)	Median (IQR)	*p-value*
Platelet count	Median (IQR)	Median (IQR)	Median (IQR)	*p-value*
Lactate dehydrogenase	Median (IQR)	Median (IQR)	Median (IQR)	*p-value*
Ferritin	Median (IQR)	Median (IQR)	Median (IQR)	*p-value*
d-dimer	Median (IQR)	Median (IQR)	Median (IQR)	*p-value*

Aspartate Transaminase (AST)	Median (IQR)	Median (IQR)	Median (IQR)	*p-value*
Alanine Transaminase (ALT)	Median (IQR)	Median (IQR)	Median (IQR)	*p-value*
Creatinine (serum)	Median (IQR)	Median (IQR)	Median (IQR)	*p-value*
Blood Urea Nitrogen (BUN)	Median (IQR)	Median (IQR)	Median (IQR)	*p-value*
Troponin i	Median (IQR)	Median (IQR)	Median (IQR)	*p-value*
Troponin t	Median (IQR)	Median (IQR)	Median (IQR)	*p-value*
High sensitivity Troponin	Median (IQR)	Median (IQR)	Median (IQR)	*p-value*
N-terminal pro-brain natriuretic peptide (NT-Pro BNP)	Median (IQR)	Median (IQR)	Median (IQR)	*p-value*
Viral persistence (measured with ddPCR)	Median (IQR)	Median (IQR)	Median (IQR)	*p-value*
Viral persistence (measured with RNA in situ hybridization)	Median (IQR)	Median (IQR)	Median (IQR)	*p-value*

3.2.4.2 Laboratory comparisons between post-acute decedents with and without PASC

Table 7b. Laboratory values for post-acute decedents overall and by PASC status

**Laboratory Assessments (latest records)**	Overall (N=)	Post-acute decedents without PASC (N=)	Post-acute decedents with PASC (N=)	Post-acute decedents without vs. with PASC
White blood cell count	Median (IQR)	Median (IQR)	Median (IQR)	*p-value*
Platelet count	Median (IQR)	Median (IQR)	Median (IQR)	*p-value*
Lactate dehydrogenase	Median (IQR)	Median (IQR)	Median (IQR)	*p-value*
Ferritin	Median (IQR)	Median (IQR)	Median (IQR)	*p-value*
d-dimer	Median (IQR)	Median (IQR)	Median (IQR)	*p-value*
Aspartate Transaminase (AST)	Median (IQR)	Median (IQR)	Median (IQR)	*p-value*
Alanine Transaminase (ALT)	Median (IQR)	Median (IQR)	Median (IQR)	*p-value*
Creatinine (serum)	Median (IQR)	Median (IQR)	Median (IQR)	*p-value*
Blood Urea Nitrogen (BUN)	Median (IQR)	Median (IQR)	Median (IQR)	*p-value*
Troponin i	Median (IQR)	Median (IQR)	Median (IQR)	*p-value*
Troponin t	Median (IQR)	Median (IQR)	Median (IQR)	*p-value*
High sensitivity Troponin	Median (IQR)	Median (IQR)	Median (IQR)	*p-value*
N-terminal pro-brain natriuretic peptide (NT-Pro BNP)	Median (IQR)	Median (IQR)	Median (IQR)	*p-value*
Viral persistence (measured with ddPCR)	Median (IQR)	Median (IQR)	Median (IQR)	*p-value*
Viral persistence (measured with RNA in situ hybridization)	Median (IQR)	Median (IQR)	Median (IQR)	*p-value*

3.3 Analytic approach to Aim 1

3.3.1 Scientific hypotheses related to Aim 1

The following hypotheses will be addressed in the analytic approach to Aim 1:

Hypothesis 1a: Prevalence and types of organ injury / disease, pathological features, and distinct phenotypes differ between acute decedents and post-acute decedents without PASC.

Note that the directionality of some hypotheses is anticipated. For example, we expect more acute diffuse alveolar damage and acute thrombi for acute decedents than for post-acute decedents without PASC.

Hypothesis 1b: Prevalence and types of organ injury / disease, pathological features, and distinct phenotypes differ between post-acute decedents with and without PASC.

Here too, the directionality of some hypotheses is anticipated. For example, we expect more organized diffuse alveolar damage and organized thrombi for post-acute decedents with PASC than for post-acute decedents without PASC.

3.3.2 Overview of analytic approach to Aim 1

We will statistically compare endpoints such as lesions in individual organs (i) between acute decedents and post-acute decedents without PASC, and (ii) between post-acute decedents with and without PASC. The primary analysis will determine if there are pathologies specific to PASC that are associated with tissue / organ / clinical dysfunction. We will also stratify our analyses by calendar quarter of enrollment or by calendar quarter of initial infection to examine possible effect modification by time of enrollment or by time of infection.

3.3.3 Overlap assessment

We will assess the overlap in covariate distributions between comparative groups (i.e., for Hypothesis 1a, acute decedents vs. post-acute decedents without PASC, and for Hypothesis 1b, post-acute decedents with vs. without PASC) using the empirical distributions of estimated propensity scores (e.g., Stuart, 2010; Bind and Rubin, 2019). We will remove "outlier" decedents with estimated propensity scores that do not overlap with the other group of decedents.

3.3.4 Covariate balance and matched-sampling strategies

We will assess balance in background covariates between the two comparative groups (acute decedents vs. post-acute decedents without PASC, and post-acute decedents with vs. without PASC), using standard causal inference techniques (e.g., "Love" plots – Ahmed et al., 2006) and use matched-sampling approaches (e.g., estimated propensity score caliper matching – Rosenbaum and Rubin, 1983) to reduce any covariate imbalance (e.g., Stuart, 2010). At this stage, a hypothetical exposure assignment mechanism consistent with the data and the matched-sampling strategy (e.g., completely randomized for a matched data set designed using the estimated propensity score) can be assumed (Bind and Rubin, 2019; Branson, 2021).

3.3.5 Fisherian inference

We will test the sharp null hypotheses described in Section 3.3.1 and report unadjusted and adjusted (Lee et al., 2016) two-sided Fisher p-values. Recall, for the comparison of acute decedents and post-acute decedents without PASC, the sharp null hypotheses will state that for each decedent, the presence and type of organ injury / disease, pathological features, and distinct phenotypes are the same had the decedent been an acute decedent or a post-acute decedent without PASC. Similarly, for the comparison of post-acute decedents with and without PASC, the sharp null hypotheses will state that for each post-acute decedent, the presence and type of organ injury / disease, pathological features, and distinct phenotypes are the same, whether the decedent had PASC or not.

For each outcome (e.g., primary endpoints described in Section 2.5.5), we will calculate Fisher p-values based on the assumed, hypothetical exposure assignment mechanism implied by the data and the

matched-sampling strategy (Bind and Rubin, 2019; Branson, 2021). For test statistics, we will use standard ones (e.g., proportion difference, Chi-squared test statistic), and less commonly-used ones (e.g., estimated coefficient from a multivariable regression model – Brillinger et al., 1978; Bind and Rubin, 2019; Sommer et al., 2021). Following the advice of Wasserstein et al. (2019), we will "not base our conclusions solely on whether an association or effect was found to be 'statistically significant' (i.e., the p-value passed some arbitrary threshold such as $p < 0.05$)", but rather also explore Fisherian intervals (e.g., Imbens and Rubin, 2015; Volfovsky et al., 2015) and consider model-based inference.

3.3.6 Model-based inference

For each type of organ injury / disease evaluated, statistical analysis will be limited to the set of decedents who did not have any record of the given organ injury / disease prior to their SARS-CoV-2 infection (the first SARS-CoV-2 infection if there is evidence of multiple infections). Note that for severe inflammation, sepsis, liver, kidney, muscle, and heart failures, the latest laboratory records (e.g., white blood cell count, creatinine levels) prior to the SARS-CoV-2 infection will be used to exclude decedents with prior pathological outcomes from the relevant statistical analyses. Prevalence of organ injury / disease, pathological features, and distinct phenotypes will be estimated using data from the acute and post-acute decedents with and without PASC. We will provide two-sided uncertainty intervals (e.g., 95% CIs) for the difference in proportions using standard large-sample approximations for the estimated variances of estimated proportion differences. Types of organ injury / disease, pathological features, and distinct phenotypes will be similarly compared using univariate, large-sample standard tests (e.g., Chi-squared test).

Pathological outcomes will be also compared between (i) acute decedents and post-acute decedents without PASC, and (ii) between post-acute decedents with and without PASC using simple (for binary outcomes), multinomial (for non-ordinal categorical outcomes), and cumulative (for ordinal outcomes) multivariable logistic regressions, as well as multivariable linear regressions (for continuous outcomes). We will provide two-sided uncertainty intervals (e.g., 95% CIs) for the estimands involving these types of outcomes.

3.4 Analytic approach to Aim 2

3.4.1 Scientific hypotheses related to Aim 2

The following hypotheses will be addressed in the analytic approach to Aim 2:

<u>Hypothesis 2a</u>: The associations of COVID-19 disease severity and treatment with pathological findings differ in decedents with prior SARS-CoV-2 infection (dying more than 30 days after SARS-CoV-2 infection) with and without PASC.

<u>Hypothesis 2b</u>: The associations of pre-infection and peri-infection risk and resiliency factors (e.g., demographic, biological factors, pre-existing clinical comorbidities) with pathological findings differ in decedents with prior SARS-CoV-2 infection (dying more than 30 days after SARS-CoV-2 infection) with and without PASC.

3.4.2 Overview

We will test hypotheses 2a and 2b. While stratifying on PASC status, we will also estimate conditional associations between (i) COVID-19 disease severity and pathological findings, and between (ii) pre-infection and peri-infection risk and resiliency factors (e.g., demographic, biological factors, pre-existing clinical comorbidities, and acute infection treatment) and pathological findings. We will then compare these conditional associations between post-acute decedents with and without PASC. We will also

stratify our analyses by calendar quarter of enrollment or by calendar quarter of initial infection to examine possible effect modification by time of enrollment or by time of infection.

3.4.3 Overlap assessment

We will assess the overlap in covariate distributions between the comparative groups (e.g., for Hypothesis 2a, post-acute decedents with severe COVID-19 and with PASC vs. post-acute decedents with severe COVID-19 and without PASC vs. post-acute decedents without severe COVID-19 and with PASC vs. post-acute decedents without severe COVID-19 and without PASC) as described in Section 3.3.3.

3.4.4 Covariate balance and matched-sampling

We will assess covariate balance between groups (e.g., defined by COVID-19 disease severity and PASC status) using standard causal inference techniques (e.g., "Love" plots) and use matched-sampling approaches (e.g., estimated propensity score matching) to reduce any covariate imbalance.

3.4.5 Fisherian inference

We will test the sharp null hypotheses described in Section 3.4.1 and report unadjusted and adjusted (Lee et al., 2016) two-sided Fisher p-values. Recall that for Hypotheses 2, we will examine whether the associations between exposures (e.g., COVID-19 disease severity, risk factors) and pathological findings are modified by PASC status. For example, for the comparison of post-acute decedents with severe COVID-19 and with PASC vs. post-acute decedents with severe COVID-19 and without PASC vs. post-acute decedents without severe COVID-19 and with PASC vs. post-acute decedents without severe COVID-19 and without PASC, the sharp null hypotheses will state that for each post-acute decedent, the pathological findings are the same regardless of COVID-19 severity (from the first SARS-CoV-2 infection if there is evidence of multiple infections) and regardless of PASC status. For each outcome, we will follow the principles of Fisherian inference as described in Section 3.4.5.

3.4.6 Model-based inference

We will examine whether the conditional associations between exposures of interest (e.g., for Hypothesis 2a, COVID-19 disease severity) and pathological findings are *different* for post-acute decedents with and without PASC by considering multivariable models regressing pathological findings outcomes on the exposures of interest (e.g., for Hypothesis 2a, COVID-19 disease severity) adjusting for background covariates (e.g., age, race / ethnicity) after stratifying by PASC status. If the estimated regression coefficients for the *background covariates* are similar for post-acute decedents with and without PASC, we will pool all post-acute decedents (i.e., with and without PASC) and consider a regression model estimating, e.g., for Hypothesis 2a, main effects on pathological findings of COVID-19 disease severity and of PASC status, as well as an interaction term between COVID-19 disease severity and PASC status. We will estimate measures of effect modifications (or interactions) and their associated two-sided uncertainty intervals (e.g., 95% CIs) using the relevant multivariable regression models. Note that for Hypothesis 2b, we will estimate measures of effect modifications (or interactions) and associated uncertainty intervals (e.g., 95% CIs) using multivariable models regressing pathological outcomes on pre-infection and peri-infection risk and resiliency factors (e.g., demographic, biological factors, pre-existing clinical comorbidities, and acute infection treatment), again stratifying by or adjusting for PASC status.

3.5 Analytic approach to Aim 3

3.5.1 Scientific hypotheses related to Aim 3

The following hypotheses will be addressed in the analytic approach to Aim 3:

Hypothesis 3a: The estimated direct effects of clinical risk factors (e.g., comorbidities, infection severity, hospitalization, intubation, steroid treatment) on pathological findings differ in post-acute decedents with and without PASC.

Hypothesis 3b: The estimated indirect effects of clinical risk factors (e.g., comorbidities, infection severity, hospitalization, intubation, steroid treatment) on pathological findings via viral persistence (investigated systematically via molecular methods) differ in post-acute decedents with and without PASC.

3.5.2 Overview

Risk factors for PASC could include viral persistence. We will examine whether clinical risk factors (e.g., comorbidities, infection severity, hospitalization, intubation, or treatments such as steroids) contribute to viral persistence (measured by ddPCR and RNA in situ hybridization) while stratifying on PASC status. We will conduct mediation analyses to test Hypotheses 3a and 3b and to estimate the direct and indirect effects (and uncertainty intervals such as 95% CIs) of clinical risk factors (e.g., comorbidities, infection severity, hospitalization, intubation, steroid treatment) on pathological outcomes via viral persistence (the possible mediator), in post-acute decedents with and without PASC. If possible, we will also stratify our analyses by calendar quarter of enrollment or by calendar quarter of initial infection to examine possible effect modification by time of enrollment or by time of infection.

3.5.3 Overlap assessment

We will assess the overlap in covariate distributions between the comparative groups (e.g., post-acute decedents with steroid treatment and with PASC vs. post-acute decedents with steroid treatment and without PASC vs. post-acute decedents without steroid treatment and with PASC vs. post-acute decedents without steroid treatment and without PASC) as described in Section 3.3.3.

3.5.4 Covariate balance and matched-sampling

We will assess covariate balance between groups defined by the clinical risk factor and PASC status using standard causal inference techniques (e.g., “Love” plots) and use matched-sampling approaches (e.g., estimated propensity score matching) to reduce any covariate imbalance.

3.5.5 Fisherian inference

We will test the sharp null hypotheses described in Section 3.5.1 and report unadjusted and adjusted (Lee et al., 2016) two-sided Fisher p-values. For example, for the comparison of post-acute decedents with steroid treatment and with PASC vs. post-acute decedents with steroid treatment and without PASC vs. post-acute decedents without steroid treatment and with PASC vs. post-acute decedents without steroid treatment and without PASC in Hypothesis 3a, the sharp null hypotheses will state that for each post-acute decedent, the pathological findings are the same regardless of steroid treatment (related to the first SARS-CoV-2 infection if there is evidence of multiple infections) and regardless of PASC status. For each outcome, we will follow the principles of Fisherian inference as described in Section 3.4.5.

3.5.6 Model-based inference

We will examine whether the conditional associations between the clinical risk factors (e.g., comorbidities, infection severity, hospitalization, intubation, steroid treatment) and pathological findings are *different* for post-acute decedents with and without PASC by considering multivariable models regressing pathological findings outcomes on the clinical risk factors adjusting for background covariates (e.g., age, race / ethnicity) after stratifying by PASC status. If the estimated regression coefficients for the *background covariates* are similar for post-acute decedents with and without PASC, we will pool all post-acute decedents (i.e., with and without PASC) and consider regression models estimating main effects of clinical risk factors and of PASC status, as well as interaction terms between clinical risk factors and PASC status. We will estimate measures of effect modifications (or interactions) and their associated two-sided uncertainty intervals (e.g., 95% CIs) using the relevant multivariable regression models.

We will estimate mediated effects and uncertainty intervals (e.g., 95% CIs) from multivariable models regressing: (i) pathological findings outcomes on clinical risk factors (e.g., comorbidities, infection severity, hospitalization, intubation, steroid treatment) and on viral persistence, and (ii) viral persistence on clinical risk factors (e.g., comorbidities, infection severity, hospitalization, intubation, steroid treatment), after stratifying on PASC status (e.g., VanderWeele, 2015). If the estimated regression coefficients for the background covariates are similar for post-acute decedents with and without PASC, we will pool all post-acute decedents (i.e., with and without PASC) and consider regression models with the relevant main effects and interaction terms.

3.6 Analytic approach to Aim 4

3.6.1 Scientific hypotheses related to Aim 4

The following hypotheses will be addressed in the analytic approach to Aim 4:

Hypothesis 4: The decedent's PASC status is associated with the presence of new lesion(s) in the CNS.

3.6.2 Overview

We will evaluate data from decedents who had both pre-infection and post-mortem brain MRIs to test Hypothesis 4 and to estimate the conditional association between PASC status and the presence of new lesion(s) in the CNS adjusting for background covariates (e.g., sex, age, race / ethnicity). If possible, We will also stratify our analyses by calendar quarter of enrollment or by calendar quarter of initial infection to examine possible effect modification by time of enrollment or by time of infection.

3.6.3 Overlap assessment

We will assess the overlap in covariate distribution between the two comparative groups (i.e., post-acute decedents with vs. without PASC) as described in Section 3.3.3.

3.6.4 Covariate balance and matched-sampling

We will assess covariate balance between post-acute decedents with and without PASC using standard causal inference techniques (e.g., "Love" plots) and use matched-sampling approaches (e.g., propensity score matching) to reduce any covariate imbalance.

3.6.5 Fisherian inference

We will test the sharp null hypothesis in Section 3.6.1. and report unadjusted and adjusted (Lee et al., 2016) two-sided Fisher p-values. For the comparison of post-acute decedents with and without PASC,

the sharp null hypothesis will state that for each post-acute decedent, the presence of new lesion(s) in the CNS does not depend on PASC status. We will follow the principles of Fisherian inference as described in Section 3.4.5.

3.6.6 Model-based inference

We will estimate the conditional association (and associated two-sided uncertainty intervals such as 95% CI) between presence of new lesion(s) in the CNS and PASC status from a logistic model regressing the presence of new lesion(s) in the CNS on PASC status adjusting for background covariates (e.g., sex, age, race / ethnicity).

3.7 Methods for missing data

Some level of missingness is anticipated for all variables being collected in this study. The primary reason for missing data will be lack of records (e.g., demographics, laboratory values) in the EHR database. Throughout, if baseline values (e.g., demographics) are missing, we will first restrict analyses to decedents with complete data and repeat analyses adjusting for missing data using multiple imputation using Multiple Imputation by Chained Equations (MICE - van Buuren and Groothuis-Oudshoorn, 2011). When it is suspected that missing covariates and outcome variables are missing not at random, we will conduct sensitivity analyses considering a range of possible deviations from the missing at random assumption (e.g., “Tipping point” analysis – Liublinska and Rubin, 2015).

3.8 Communications

A Manual of Procedures (MOP) will be provided to study coordinators. The MOP will contain information on: how to screen decedents and obtain informed consent, and how to use the Electronic Data Capture (EDC) system and case report forms (CRFs). Site staff will be prepared for study conduct, including training in the protocol and EDC procedures, review of Good Clinical Practices, and review of Health Insurance Portability and Accountability Act (HIPAA) regulations. The clinical sites will be trained to comply with reporting requirements to promote standardization of study-related procedures. The REDCap EDC system integrates site onboarding, training, study documents, and resources in a central location accessible by authenticated users. A distributed communication network (email list server, website) will be used. The website will have a public section with general information about study protocols and a password-protected section for study staff with study documents (e.g., protocol, MOP), contact information, meeting minutes and agendas, and other important study resources. The RECOVER DRC will monitor screening and enrollment throughout the study. If concerns arise, the DRC and CSC will work with sites to ensure sufficient recruitment.

3.9 Observational Study Monitoring Board

Oversight of data and safety for all three RECOVER cohort studies will be provided by a PASC OSMB appointed by National Heart, Lung, and Blood Institute (NHLBI). Relevant to the autopsy study, the OSMB will meet at least twice a year to review data on data quality and study recruitment as described in the committee charter, and make recommendations about study conduct to the NHLBI. As the RECOVER study does not involve any interventions, an early stopping rule for efficacy or futility is not indicated.

After each OSMB meeting, the OSMB determination letter and a summary report of adverse events will be prepared within 30 days and will be distributed by NHLBI staff to each principal investigator and study coordinator for review. The summary report will contain the following information:

- A statement that an OSMB review of outcome data and information relating to study performance across all centers took place on a given date;
- A statement that a review of recent literature relevant to the research took place;
- The OSMB's recommendation with respect to progress or need for modification of the protocol or informed consent. If the OSMB recommends changes to the protocol or informed consent, the rationale for such changes and any relevant data will be provided;
- A statement that if concerns are identified, the NHLBI Program Official will communicate these promptly to the investigators.

4 References

1 Ahmed, A, Husain, A, Love, TE. Heart failure, chronic diuretic use, and increase in mortality and hospitalization: an observational study using propensity score methods. *Eur Heart J* **27**, 1431–1439 (2006)

2 Baytas, I. M., Lin, K., Wang, F., Jain, A. K. & Zhou, J. PhenoTree: Interactive Visual Analytics for Hierarchical Phenotyping From Large-Scale Electronic Health Records. *IEEE Transactions on Multimedia* **18**, (2016).

3 Bind M-AC, Rubin DB. Bridging observational studies and randomized experiments by embedding the former in the latter. *Statistical Methods in Medical Research,* **28**, 7, Pp. 1958-1978 (2019).

4 Box GEP, Cox DR. An analysis of transformations. *J R Stat Soc B.* **26**:211–52 (1964).

5 Branson Z. Randomization tests to assess covariate balance when designing and analyzing matched datasets. *Observational studies* **7**, 1-36 (2021)

6 Brillinger, DR, Jones, LV, Tukey, JW. Report of the Statistical Task Force to the Weather Modification Advisory Board. In: Anonymous The management of weather resources, **Volume II**, pp. 25, F-5 (1978)

7 Imbens, G, & Rubin, D. Causal Inference for Statistics, Social, and Biomedical Sciences: An Introduction. *Cambridge: Cambridge University Press* (2015).

8 Lee, J, Forastiere L, Miratrix L, and Pillai N. More Powerful Multiple Testing in Randomized Experiments with Non-Compliance. *arXiv*:1605.07242 (2016)

9 Liublinska V, Rubin DB. Sensitivity analysis for a partially missing binary outcome in a two-arm randomized clinical trial.*Stat Med*., **33**, 4170-4185 (2014)

10 Rosenbaum P and Rubin DB. The central role of the propensity score in observational studies for causal effects, *Biometrika*, **70,** 41–55 (1983)

11 Rubin, DB, Bayesian inference for causal effects: the role of randomization. *Annals of Statistics* **6**, 34-58 (1978)

12 Sommer A, Leray E, Lee Y, and Bind MA. Assessing environmental epidemiology questions in practice with a causal inference pipeline: An investigation of the air pollution-multiple sclerosis relapses relationship. *Stat Med*, **40**, 6, Pp. 1321-1335 (2021).

13 Stuart, Elizabeth A. “Matching methods for causal inference: A review and a look forward.” Statistical science : a review journal of the Institute of Mathematical Statistics, vol. **25**,1 (2010).

14 van Buuren, S. & Groothuis-Oudshoorn, K. mice: Multivariate imputation by chained equations in R. *Journal of statistical software* **45**, 1–67 (2011)

15 VanderWeele, T. Explanation in causal inference : methods for mediation and interaction. *Oxford University Press* (2015)

16 Volfovsky A, Airoldi E, Rubin DB**.** Causal inference for ordinal outcomes. arXiv:1501.01234 (2015)

17 Wasserstein R, Schirm A, Lazar N. Moving to a World Beyond “p < 0.05”. *The American Statistician,* **73**, 1-19 (2019

18 Wood, S. & Wood, M. S. Package ‘mgcv.’ *R package version* 1, 29 (2015)
