# Supplementary material for: Researching COVID to enhance recovery (RECOVER) tissue pathology study protocol: Rationale, objectives, and design
Source: PLoS One. 2024 Jan 10;19(1):e0285645. doi: 10.1371/journal.pone.0285645 (PMC10781091; doi:10.1371/journal.pone.0285645)
Supplement: S2 File — (PDF) [file pone.0285645.s004.pdf]

PASC Biorepository Core (PBC) Biospecimen Collection Procedures: Autopsy Cohort

Version: 3.1 **Published: 2/27/2023**

Table of Contents

I. Introduction
- A. Glossary and Keywords

II. Procedures
- A. RECOVER Autopsy Protocol Guideline
- B. RECOVER Equipment Reference Guide
- C. RECOVER PBC Autopsy Training Checklist
- D. Ordering RECOVER Autopsy Kits from PBC
- E. RECOVER Autopsy Biospecimen Collection
  1. Pre-Collection
  2. Blood SST, Blood Spot, CSF, Bronchial Swab, and Stool Collection
     a) SST Processing
  3. Tissue Collection
     a) Block Summary by Tissue Type
  4. Initial Packaging
     a) Frozen Matrix Tubes
     b) FFPE and Cryosette™ Packaging
  5. Final Packaging & Shipping
     a) Frozen specimens
     b) Refrigerate specimens

III. Appendices
- A. Autopsy Kit Contents
- B. RECOVER collection labels and collection tube guide
- C. G-001.03 RECOVER Autopsy Template (1st laminated document)
- D. SOP-021.03 RECOVER Autopsy Specimen Key (2nd laminated document)
- E. CL-002.04 RECOVER Autopsy Checklist
- F. Master Troubleshooting Guide

IV. Forms
- A. F-013.04 PASC Biorepository Core Requestion Form: Autopsy Cohort

V. Revision/Document History

VI. Approvals

I. INTRODUCTION

The purpose of this document is to provide RECOVER collection sites with standardized steps to properly execute the PASC Biorepository Core (PBC) protocol aims. This includes the successful collection, processing, storage, and shipment of specimens for long-term biorepository storage.

Prior to executing these procedures, staff is required to complete the appropriate training checklist for the respective cohort. In addition, staff are required to complete a "Dangerous Good Training module." Below is a link to the Mayo Clinic Laboratories (MCL) provided training. Comparable training provided by your institution would suffice and could be used instead.
https://news.mayocliniclabs.com/2021/06/15/dangerous-goods-training-2/

Trained Staff will be responsible for following the instructions provided in this manual. The Trained Staff are also expected to exercise their best clinical judgment and perform these procedures under standard autopsy and good laboratory practices.

Contacting the PBC for Support:

Submit a RECOVER Help Desk ticket and choose Biospecimen topic category for questions pertaining to RECOVER biospecimens, procedures, and PBC kits.

If during sample collection or processing there is an unusual problem or occurrence that is not covered in these procedures including the Master Troubleshooting guide, and you are not sure how to proceed, please stop and contact the RECOVER PBC at the email address below for guidance.

Email: RECOVERPBC@mayo.edu

A. Glossary and Keywords

Term	Definition
PBC	PASC Biorepository Core
DRC	Data Repository Center
CSC	Clinical Science Core
CSF	Cerebrospinal fluid
FFPE	Formalin fixed paraffin embedded
SOP	Standard Operating Procedures
IATA	International Air Transport Association
DOC Shipping	Day of collection (DOC) shipping describes the process of shipping refrigerate specimens from one or more participant on the day of collection.
Requisition Form	A form included in the kit contents that has information about the collection event. Filled out as a collection of biospecimens occur.
Manifest	An on-site printed PDF of the REDCap Biospecimen form. Manifests are included physically in the refrigerate and frozen shippers sent to the PBC.
Shipper	Styrofoam container with a cardboard shell used to ship samples to the PBC. Shippers come pre-labeled with all DOT/IATA compliant markings and pre-paid FedEx airbill.
MCRCRP	Mayo Clinic Research Client Request Portal
PPE	Personal Protective Equipment such as gloves, lab coats, safety glasses etc.
Ambient/Room Temperature	19°C – 23.5°C

Refrigerated Temperature	2°C – 8°C
Frozen Temperature	-90°C – -65°C
1.4 mL Matrix Aliquot Tubes	A 1.4mL matrix tube provided in the PBC kit used for sample aliquoting that occurs locally, on-site. All 1.4 Matrix aliquot tubes are unlinked. Tubes have a pre-assigned manufacturer barcode ID affixed in both 2D and 1D format (see 1.4 Matrix aliquot tube).

RETURN TO TABLE OF CONTENTS

II. PROCEDURES

A. RECOVER Autopsy Protocol Guideline

1.0 PURPOSE

This document is designed to provide an overview of the samples collected for the Autopsy Cohort for the RECOVER.

2.0 Biospecimen Collection

Collection Container	# of Container(s)	Collection tube Volume (mL)	Fraction	Aliquot Volume	Shipping Temperature
Blood Spot Card	2	N/A	Whole Blood	One card with 4 blood spots	Refrigerate
SST	2	8.5	Serum	10 x 1 mL	Frozen
Cerebrospinal Fluid (CSF)	10	N/A	CSF	10 x 1 mL	Frozen
Bronchial Swab	1	N/A	Cells	Entire tube	Frozen
Stool	1	25	Stool	25 mL	Frozen
52 + 4 Tissues (In duplicate)	112	Cryosette™	Snap Frozen Tissue	17 mm diameter, not to exceed 4 mm in thickness x 2	Frozen
52 + 4 Tissues	56	Standard Tissue Cassette	FFPE Tissue	2 x 2 x 0.4 cm	Refrigerate
Brain	N/A	N/A	N/A	N/A	Refrigerate

3.0 RECOVER Kit Label Example

NOTE: Please ignore minor differences in tube label appearance seen in these procedures from those on the tubes in PBC kits. See image below for a RECOVER Kit label example.

NOTE (2): Please note that this is a temporary label design, as the PBC continues to work through the long-term RECOVER study label development and implementation.

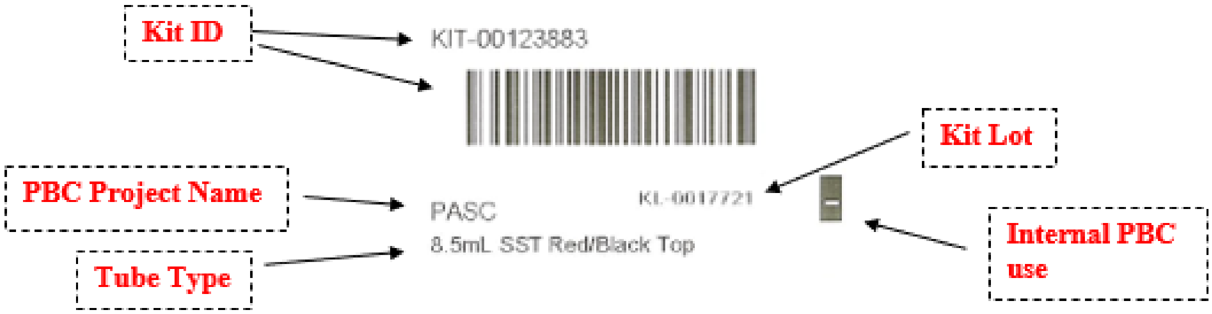

Kit ID: A unique number assigned to all contents in one collection kit, also represented in barcode format for scanning.
PBC Project Name: Project name used internally at PBC
Tube Type: Description of tube type
Kit Lot: Internal identifier used at PBC to track the kit during production

RETURN TO TABLE OF CONTENTS

B. RECOVER Equipment Reference Guide

1.0 INTRODUCTION AND PURPOSE
This document is meant to outline equipment specifications and provide product references for the collection, processing, and stabilization of SST tubes for the RECOVER.

2.0 SCOPE
Acceptable equipment or products are not limited to those listed in this document. Products listed in this document are not being endorsed in any way for a vendor or manufacturer by Mayo Clinic.

3.0 EQUIPMENT GUIDELINES

3.1 CENTRIFUGE

Any product that meets these specifications would be considered acceptable.

- Rotor Type: swinging bucket rotor is optimal, fixed angle is acceptable alternative
- Speed: must be able to spin up to 1,300 x g
- Temperature: Ambient
- Features/Additional considerations:
  - Includes containment cap
  - Programmable timer

Examples of Acceptable Centrifuges. The following list of centrifuges meet the requirements for processing RECOVER specimens and are considered suitable for a laboratory benchtop. Acceptable products are not limited to those listed.

NOTE: It is critical to ensure swinging bucket rotors with the correct adapters are identified when purchasing. Contact the retailer/manufacturer to provide the correct product numbers.

Manufacturer	Product Name	Product Number	Electrical Requirements	Capacity	Hyperlink
ThermoFisher Scientific	Sorvall™ ST 16 Centrifuge	75004240	230V 50/60Hz	4 x 400mL	LINK
		75004241	120V 60Hz		
		75004243	100V 50/60Hz		
		75007201	230V 50/60Hz		
		75007202	100V 50/60Hz		
Beckman Coulter	Allegra X-30	B06314	100V 50/60Hz	4 x 400mL	LINK
		B06315	220-240 V 50/60Hz		LINK
NuWind	General Purpose Bench Top 3 Liter Ventilated Centrifuge	NU-C300V	120V 60Hz	4 x 750mL	LINK

3.2 BARCODE SCANNERS

Barcode scanners improve the efficiency and quality of data entry and are highly recommended. Examples of Acceptable Barcode Scanners include the following.

Manufacturer	Product Number	Connection Type	1D/2D Scanning	Wired	Hyperlink
Honeywell	Xenon 1900h	USB	Both	Yes	LINK
Honeywell	Xenon 1902h	Bluetooth	Both	No	LINK

3.3 THERMAL TRAYS

Thermal trays aid in freezing tissues. Example of acceptable thermal trays include the following.

Manufacturer	Product Name	Product Number	Hyperlink
Corning	ThermalTray™ Thermo-conductive Platforms	432074	LINK
Corning	9L Ice Pan	432093 - 432099	LINK

Thermal trays need to be cooled by pellets of dry ice or liquid nitrogen. The thermal tray should be placed in a container with dry ice pellets or liquid nitrogen such as Styrofoam box or an ice pan.

3.4 LAMINATED TEMPLATES AND KEY RECOMMENDATIONS

Each RECOVER autopsy collection site will be provided with one each of the laminated documents, G-001.03 RECOVER Autopsy Template and SOP-021.03 RECOVER Autopsy Key.

It is the recommendation of the PBC that StatMark™ Pens are used to record data on the laminated cards, the Cryosette™ 21-count Racks, and the 96-well matrix storage box. The quick drying ink is highly resistant to aqueous based stains, alcohols, and xylene. The ink can be removed using Asepti-Zyme™ enzymatic detergent or other institutional enzymatic detergent. To remove any residual StatMark™ residue, wipe clean with acetone.

NOTE: When the either of the laminated cards begin to degrade and become unusable, please send a request for replacement to the RECOVER PBC for a replacement of either G-001.03 RECOVER Autopsy Template or SOP-021.03 RECOVER Autopsy Key.

Email: RECOVERPBC@mayo.edu or submit a RECOVER Help Desk Ticket

StatMark™ Pens

Vendor	Vendor Number	Hyperlink
Thomas Scientific	72109-12	LINK
Sigma Aldrich	Z648191	LINK

Asepti-Zyme™ enzymatic detergent

Vendor	Vendor Number	Hyperlink
Cardinal Health	6023175	LINK
McKesson	962413	LINK
Surgo	900-23175	LINK

RETURN TO TABLE OF CONTENTS

C. RECOVER PBC Autopsy Training Checklist

1.0 INTRODUCTION AND PURPOSE

This document is designed to standardize the procedure for training new Program staff at the collection sites on the processes for the RECOVER.

2.0 SCOPE

This document is a training checklist for new Program staff at the collection sites on the biospecimen collection processes for RECOVER.

3.0 SOP Review for Autopsy Protocol

The trainee must read though the appropriate SOPs to match the work that they will be responsible for:

- ☐ RECOVER Autopsy Protocol Guideline
- ☐ RECOVER Autopsy Equipment Reference Guide
- ☐ Ordering RECOVER Kits from PBC
- ☐ RECOVER Autopsy Biospecimen Collection
- ☐ F-013.04 PASC Biorepository Core Requisition Form: Autopsy Cohort
- ☐ G-001.03 RECOVER Autopsy Template
- ☐ SOP-021.03 RECOVER Autopsy Key
- ☐ CL-002.03 RECOVER Autopsy Checklist for Packaging & Shipping
- ☐ Dangerous Goods Training (from Mayo Clinic Laboratories, linked below, or provided by collection site institution)
    - https://news.mayocliniclabs.com/2021/06/15/dangerous-goods-training-2/

RETURN TO TABLE OF CONTENTS

D. Ordering RECOVER Kits from PBC

Below are protocol specific instructions for using the Mayo Clinic Research Client Request Portal (MCRCRP) for ordering kits from the PASC Biorepository Core (PBC). For additional MCRCRP training and education resources, including video tutorials, please navigate to the external customer education site.

Create an Account – Quick Reference Guide | Video Tutorial

1. To create an account, navigate the Mayo Clinic Research Client Request Portal, MCRCRP. It is required to use a professional email address to create the account.
2. Once an account is created, there will be an email sent to the address used for account creation. Click on the activation link to be directed to the portal.
3. Navigate to the portal and click "Login"
4. Click "create your account" and complete the required fields.
5. Follow the onscreen instructions to activate the account.

Submit an Access Request – Quick Reference Guide | Video Tutorial

An access request is a required step ***after*** account creation and ***before*** a request is submitted. When logging in for the first time after creating an account, you will automatically be directed to create an Access Request.

1. **Access Request**: From the drop-down menu, select Kit Request. Initially, this field will default to Kit Request.
2. **Kit Location:** Enter **Minnesota**
3. **Study:**
   A. For Autopsy Cohort: **Ext. IRB RECOVER PASC Autopsy Cohort Cicek R3006135**
4. **General Comments /reasons**: Name of your Organization (Examples: Mount Sinai, Columbia, UTSA, etc.)
5. Click **Submit** near the bottom of the page to submit the request for approval.

Submit a Kit Request– Quick Reference Guide | Video Tutorial

Once an access request approval email is received, kit requests can be submitted by following these steps.

1. Click **Biobank** (A) under the **Features** section of the navigation menu.

2. Click **Create Kit Request** (B) to start a new request.

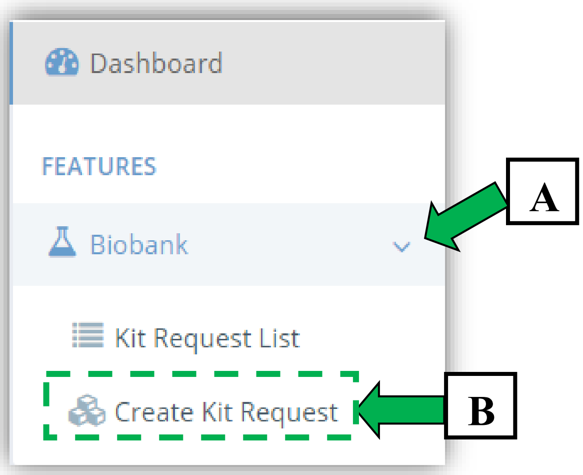

3. Complete the required information on page one of the request:
    a. **Study Site**:
        iii. For Autopsy Cohort: **Ext. IRB (RECOVER_PASC Autopsy Cohort) Cicek R3006135**
    b. **Mayo Location Fulfillment Center**: BAPLB-Minnesota
    c. **Client ID:** B93373009
    d. **Requestor Telephone:** provide a phone number if not automatically populated.
4. **Visit**: Select the first visit requiring a new kit. This selection will populate available kit types.
5. **Kit Type:** Select the kit type being requested. See Appendix A for the RECOVER Kit contents.
6. **Number of kits:** Free text type or use the + or – buttons (A) to specify the quantity of the Kit Type you would like to add to your request.
7. Click **Add New** (B) to add the kits to your order. The items should now appear on the right side of your screen (C) near the bottom under the Kit(s) List Information menu.

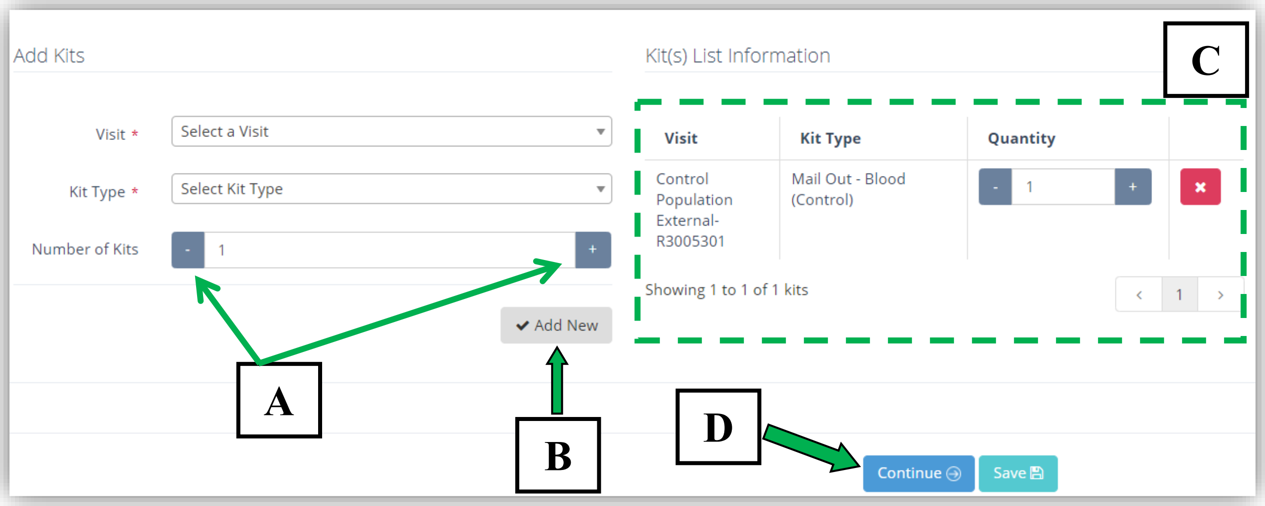

8. Repeat steps 4-7 until all kit types have been added to your request.
9. Click **Continue** (D) to advance to the next page.
10. Complete the required delivery information on page two of the request:
    a. **Delivery Method**: Select **Ship External** from the drop down menu.
        i. **Shipping Address** – Select from the drop down menu (A) the address you would like to ship the kits to. To add an address to the drop down menu click **Create adhoc Address** (B). Complete the required fields and click **Save Adhoc Address.**

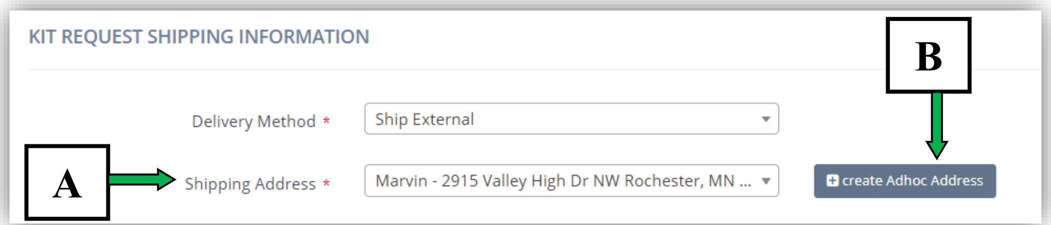

ii. **Rush Delivery** – Make this selection if your kit(s) need to be delivered prior to the first available date in the Date Kits Needed field. If selected, the portal will prompt you to enter a FedEx account number. Please enter 0000000000 as a placeholder.

iii. **Home Delivery** – leave this as No.

iv. **Saturday Delivery**- if the kits require FedEx delivery on Saturday please mark this selection as YES. This should only be select if rush delivery was selected. If the recipent location is not staffed on Saturday, do not select this option.

v. **Date Kits Needed-** Please select the date you would like the kits delivered from the pop-up calendar. If the kits are needed prior to the first available date please make the Rush Request selection and return to the calendar to select the desired delivery date.

vi. **FedEx Ship Manager:** click this button to enter additional shipping information.

   a. **Service Type** – Select **R-FedEx Ground Service**. If "Rush Delivery" was selected use an overnight or 2 Day service option.

   b. **Hold at Location –** Select **No**. Only select yes if the recipent would rather drive to a FedEx store/station to pick-up the package.

   c. **Signature Options** – The standard practice is to select "**No Signature**." If you would like the recipent to have to sign for the package select the appropriate signature option.

   d. **Ship Alert –** it is required to specify at least one email address to receive automated delivery notifications from FedEx.

      i. Click **Ship Alert**.

      ii. Click **Add New.**

      iii. Select **Recipient** from the "Alert Recipient Type" drop down.

      iv. Enter a vaild email address in the "Email ID" field.

      v. Select the notfications you would like to receive from FedEx (A).

      vi. Click **Save Ship Alert** (B).

      vii. Repeat steps ii-vi to request FedEx notifications for up to two additional email addresses.

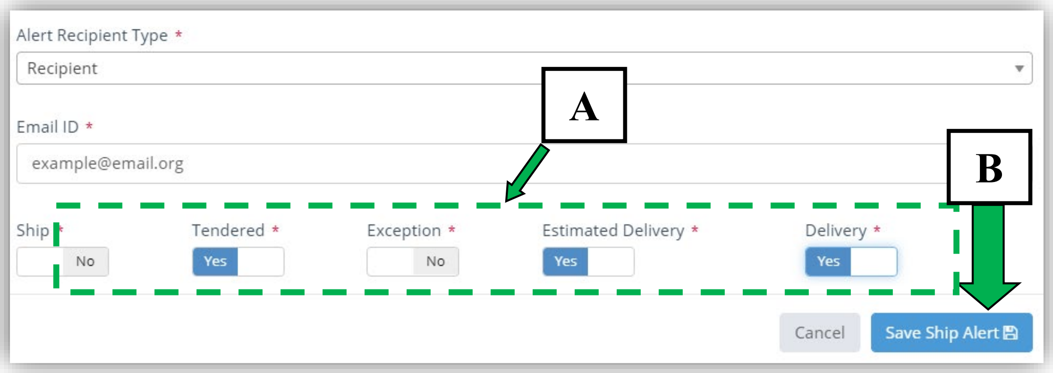

11. Click **Continue** to advance to the last page. Review the details of your order carefully.
12. Once you have reviewed the request details and added attachements (if necessary) click **Submit**.
13. Expect arrival of RECOVER kits within 2 weeks. Please submit a RECOVER Help Desk Ticket or notify RECOVERPBC@mayo.edu if any issues with ordering or a delay in arrival of kits.

Profile Manangment

Once an acount has been created the following steps can be used to manage your profile.

1. To access your account information hover over the used ID (A) in the upper right hand corner of the home page and click on the **My Profile** button (B).

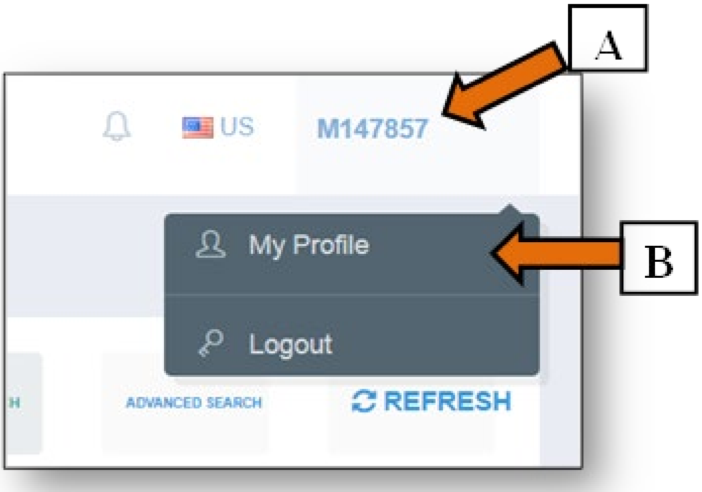

2. **Account Information**- changes to your account information must be submitted through a request. Please follow this hyperlink to update your account information.

3. **Address Information**- To simplify the kit ordering process, commonly used shipping locations can be stored to an address book. Use the following steps to add and save addresses to your account. Saved addresses cannot be accessed by other system users.
   a. Click on **Address Information**.

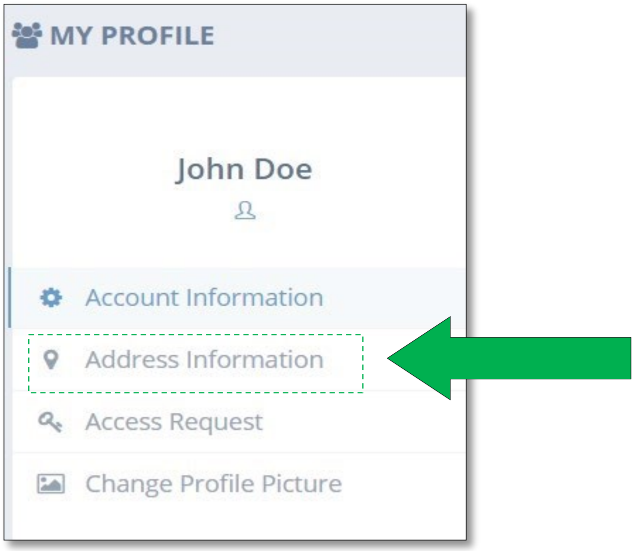

   b. On the right-hand side click **Add address.**

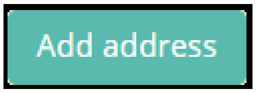

   c. Enter the required information into the pop-up window and click Save.
   d. After an address has been saved it can be selected as a default, edited, or deleted at any time by clicking the individual icons (A) listed next to the address (hint: hover over an icon to see a description).

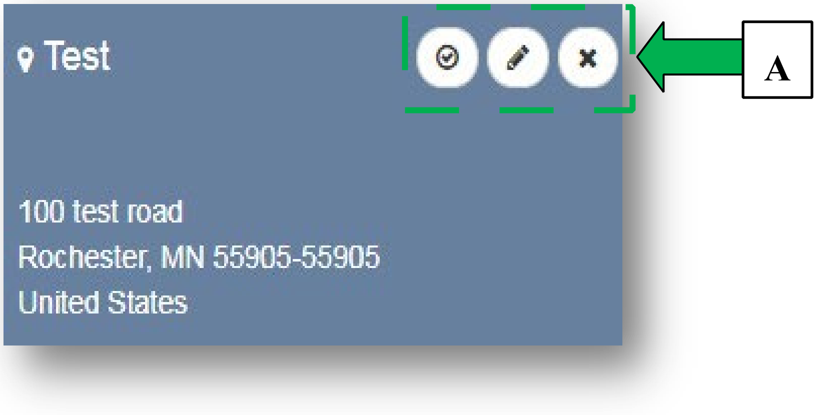

4. Change Profile Picture- you may add and edit an account profile picture at any time using this menu.

RETURN TO TABLE OF CONTENTS

E. RECOVER Autopsy Biospecimen Collection

1.0 INTRODUCTION AND PURPOSE

This document is designed to standardize the procedure for collecting the autopsy specimens for the RECOVER.

2.0 PROCEDURES

2.1 MATERIALS (Included in Kit)

BD Vacutainer® 8.5 mL SST (2)
1.4 mL Matrix tubes (20)
QIAcard FTA Classic (100) WB120205(1)
CLASSIQSwabs Plastic-Swab Bronchial Swab (1)
3.0 mL MicroTest™ M4RT Transport vial (1)
FFPE Tissue Cassettes (56)
4-Count FFPE Block Transport Container (14)
Cryosettes™ (112)
Cryosette™ 21-count Rack (6)
96-well Matrix Storage Box (1)
25 mL Stool Container with Spoon (1)
Parafilm
6 x 9 Biohazard bags (2)
4 x 6 Biohazard bag (1)
Mailable Biospecimen Bag for Bronchial Swab (1)
12 x 15 Return Biohazard bags (2)
Requisition Form
Checklist

MATERIALS (Not included in Kit)

Tape
Manila envelopes

3.0 BIOSPECIMEN COLLECTION

3.1 Pre-Collection Procedure
3.2 SST Blood Collection
    3.2.1 SST Processing
3.3 CSF Collection
3.4 Blood Spot Card
3.5 Bronchial Swab Collection
3.6 Stool Collection
3.7 Tissue Collection
    3.7.1 FFPE (52 Defined Sites +/- 4 Undefined Sites)
    3.7.2 Snap Frozen, in duplicate (52 Defined Sites +/- 4 Undefined Sites)

4.0 PACKAGING & SHIPPING

4.1 Frozen Matrix Tubes
4.2 FFPE and Cryosette™ Initial Packaging
4.3 Final Packaging & Shipping
    4.3.1 Frozen Specimens
    4.3.2 Refrigerate Specimens

3.0 BIOSPECIMEN COLLECTION

3.1 PRE-COLLECTION PROCEDURE

NOTE: Prior to kit registration, confirm that the KIT-ID on the kit box matches the KIT-ID on the requisition form inside the kit box and all collection containers have the same KIT-ID number prior to collection. If it does not match, put the kit aside and submit a RECOVER Help Desk Ticket for Biospecimen resolution.

1. Register the kit to the decedent using REDCap:
    a. Confirm the accuracy of the Date of Biospecimen Form collection field. This will autopopulate upon opening the Biospecimen form.
    b. Enter the Date and Time of Fixation.
    c. From the dropdown, select the Kit Type: RECOVER – Autopsy.
    d. Link the biospecimen kit to the decedent by scanning the KIT-ID barcode on the kit box into the KIT-ID entry 1 and KIT-ID entry 2 fields. It is highly recommended to use a barcode scanner to enter the barcode. The KIT-ID is entered twice to ensure the accuracy of the KIT-ID entered. When entering the KIT-ID it needs to be in the exact format as it is on the kit box label. Required format is KIT-XXXXXXXX

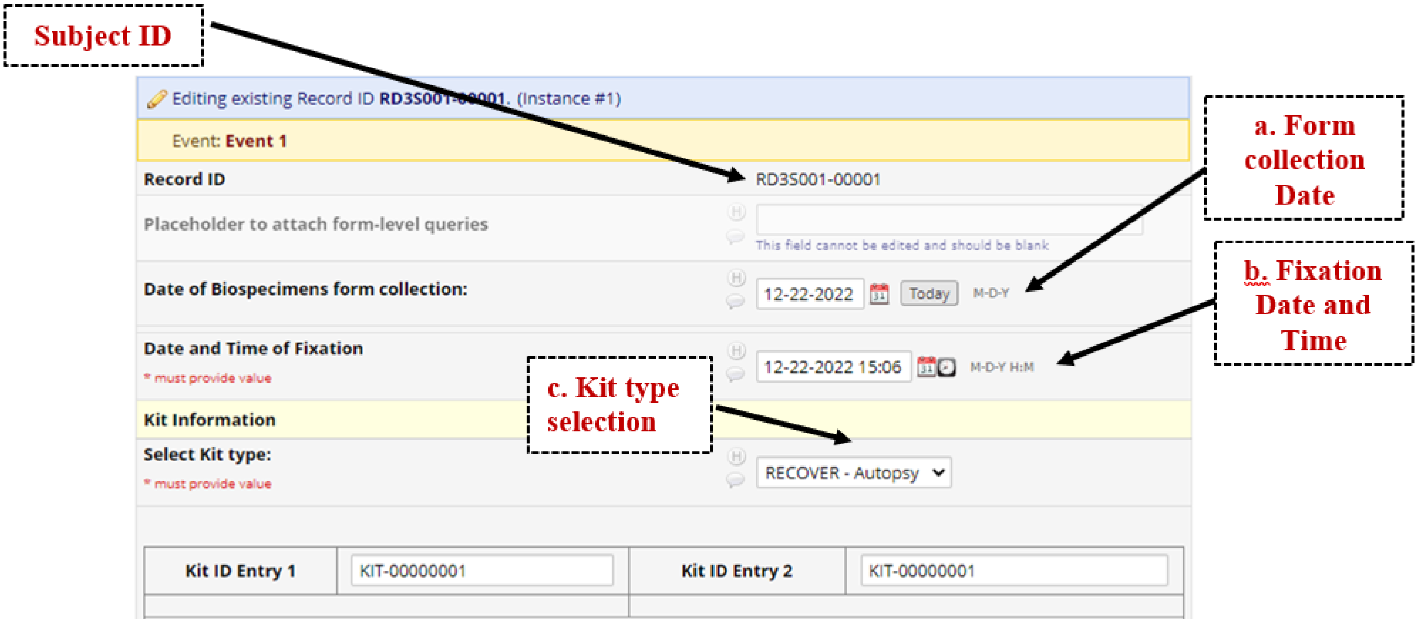

NOTE: The image above contains example data. Please ensure you are entering the Kit-ID for the set of collection tubes/containers in hand.

2. Write the Subject ID on the side of the Cryosettes™ Racks **and** the side of the 96-count matrix storage box included with the kit using a StatMark™ Pen. Writing the Subject ID on the sides while at ambient temperature prior to utilization is recommended due to complications of writing on a frozen racks if labeling at the time of collection.
3. **KIT-IDs are required on all FFPE cassettes and Cryosettes™ containers. Sites should hand write the participant's KIT-ID on the tissue containers using a StatMark™ pen if not already pre-labeled.**

4. At the time of collection, confirm the identity of the decedent providing the specimen by using the information in REDCap.
5. Confirm that the KIT-ID on the kit box matches the KIT-ID registered to the decedent in REDCap.

NOTE: There is no decedent information on the label; therefore, it is critical that the Kit-ID on the box matches to the Kit-ID registered to the participant.

3.2 SST BLOOD COLLECTION

1. Obtain blood from either the aorta or femoral vein, assemble collection device and attach the first collection tube onto the collection assembly.
2. Collect the two SST tubes.
3. Once collected, each SST tube must be inverted 5 times by quick-mixed and gentle inversions.

   NOTE: Quick mix the tubes by gentle inversions ensures that the additives on the inner walls of specimen tubes are properly mixed with the collected blood and ensures proper clotting and processing.
4. Allow the SST tubes to clot for a minimum of 30 minutes at room temperature in an upright position prior to centrifugation.
5. Once the samples have been collected, record the Subject ID, Date and Time of Autopsy, and Hours since Death on the Requisition Form.

   NOTE (1): Please ensure the Subject ID is recorded on the requisition form (see REDCap image above)

   NOTE (2): The specimen Collection Time will be documented as the Date and Time of Autopsy; and the Hours After Death will also be captured on the requisition form.

3.2.1 SST TUBES PROCESSING

NOTE: Processing SST tubes **must** be completed within one hour (preferably) but no longer than 4 hours after collection.

1. Keep the SST collection tube at room temperature until the processing is completed.
2. Allow the SST tube to clot for a minimum of 30 minutes at room temperature in an upright position prior to centrifugation.
3. Centrifuge the SST tubes at room temperature at a speed of **1300 x g** for **10 minutes in a swinging bucket centrifuge (optimal) or for 15 minutes in a fixed-angle centrifuge.**

To convert from G-force RCF (x g) to RPM, use the following formula:

$$RPM = \sqrt{\frac{RCF}{r \times 1.118}} \times 1{,}000$$

- RPM= Rotational speed of the centrifuge (revolutions per minute)
- RCF= Relative Centrifugal force
- r = Rotor Radius (mm), which is measure from the center of centrifuge rotor to the inner bottom of the centrifuge bucket holding the specimens as seen in the image below.

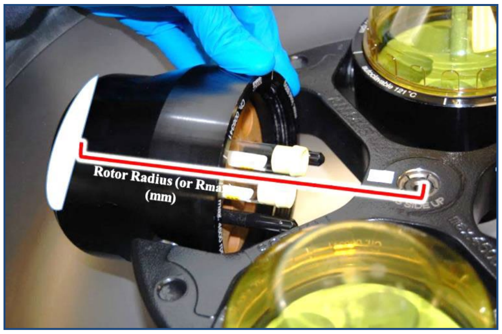

4. Confirm that the SST specimen was processed successfully. Successfully processed SST specimen would have the gel layer migrate from the bottom of the tube to approximately the mid-point of the tube following centrifugation. A distinct and separate layer of serum (in SST tubes) should be observed on top of the gel layer. The whole blood layer should be observed as a separate layer beneath the gel layer. The images below illustrate an example of SST specimen before and after centrifugation.

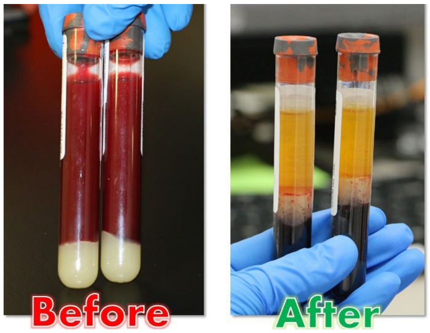

5. Once centrifuged, in REDCap, enter the SST specimen IDs into the sample ID fields. Each SST tube in the kit will have a different specimen ID.
6. In REDCap, record the time and date when the SST tubes were placed into the centrifuge.

SST Collection and Processing

SST Collection 'Please enter the sample id and centrifuge date time for each parent SST tube'			
**SST1 Sample ID**	2021096001	**SST1 Centrifuge Date/Time**	05-11-2022 09:09:11 M-D-Y H:M:S
**SST2 Sample ID**	2021090610	**SST2 Centrifuge Date/Time**	05-11-2022 09:09:14 M-D-Y H:M:S

7. Select Locally on the processing prompt.

Is the SST processing happening locally (at your study site) or centrally (at the PBC)?

- (●) Locally
- ( ) Centrally

reset

8. Obtain 10 1.4 mL matrix tubes from the kit.
9. Uncap the SST tube.
10. Using a pipette, transfer 1000µL into each of the 1.4 mL matrix tubes. **It is recommended to use a P1000 pipette to aliquot the serum. Do not add more than 1000µL to the matrix tube.**

11. Cap all the matrix tubes immediately. **It is critical that the caps are screwed securely to the tubes.**
12. Discard the SST collection tubes according to your institutional guidelines.
13. In REDCap, scan the matrix aliquot IDs into the SST aliquot ID fields and record the corresponding aliquot volume (in microliters) and freeze time. **This step is key to properly link the aliquots to the Subject ID and to capture the associated specimen data.**

SST 1 'Please enter the volume (in microliter) and freeze date time associated with each aliquot processed from the parent tube SST 1'			
SST1 Aliquot 1 ID	0228946297	SST 1 Aliquot 1 Volume (µl)	1000
		SST 1 Aliquot 1 Freeze Date/Time	05-11-2022 09:13:00 M-D-Y H:M:S
SST 1 Aliquot 2 ID	0226747800	SST 1 Aliquot 2 Volume (µl)	1000
		SST 1 Aliquot 2 Freeze Date/Time	05-11-2022 09:13:00 M-D-Y H:M:S
SST 1 Aliquot 3 ID	0228946160	SST 1 Aliquot 3 Volume (µl)	1000
		SST 1 Aliquot 3 Freeze Date/Time	05-11-2022 09:13:00 M-D-Y H:M:S
SST 1 Aliquot 4 ID	0306924450	SST 1 Aliquot 4 Volume (µl)	1000
		SST 1 Aliquot 4 Freeze Date/Time	05-11-2022 09:13:00 M-D-Y H:M:S
SST 1 Aliquot 5 ID	0306927098	SST 1 Aliquot 5 Volume (µl)	1000
		SST 1 Aliquot 5 Freeze Date/Time	05-11-2022 09:13:00 M-D-Y H:M:S

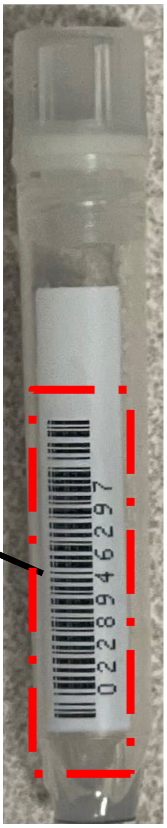

NOTE: If not all 10 aliquots were obtained, leave unused fields blank and dispose of any unused aliquot tubes. Do not send empty aliquot tubes to the PBC.

14. Record the volume of each aliquot, round and record the final volume of the aliquot that is created down to the nearest of 250µL. If the use of P1000 is not possible, use the disposable pipette. In the case of using the disposable pipette, the Trained staff must round the approximate final volume of the aliquot to the nearest 250µL increment (i.e., ½ full = 500µL, and full = 1000µL). **Do not add more than 1000µL to the matrix tube.** (See images below)

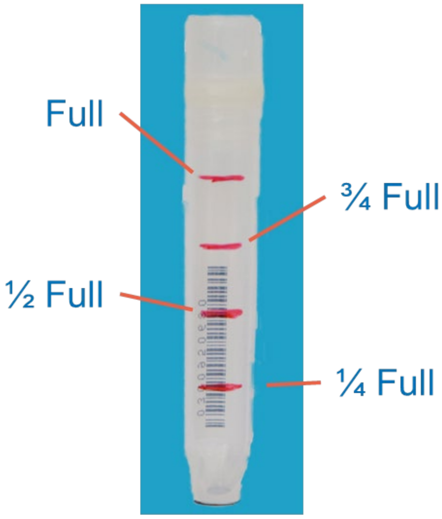

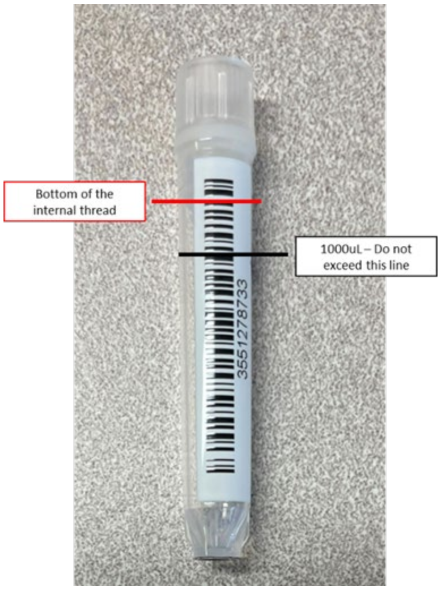

15. After Processing of the SST tubes, store serum matrix tubes in **Row A** of the 96-well matrix tube storage box at frozen temperatures (-80°C) until specimens are ready to be shipped to the PBC.

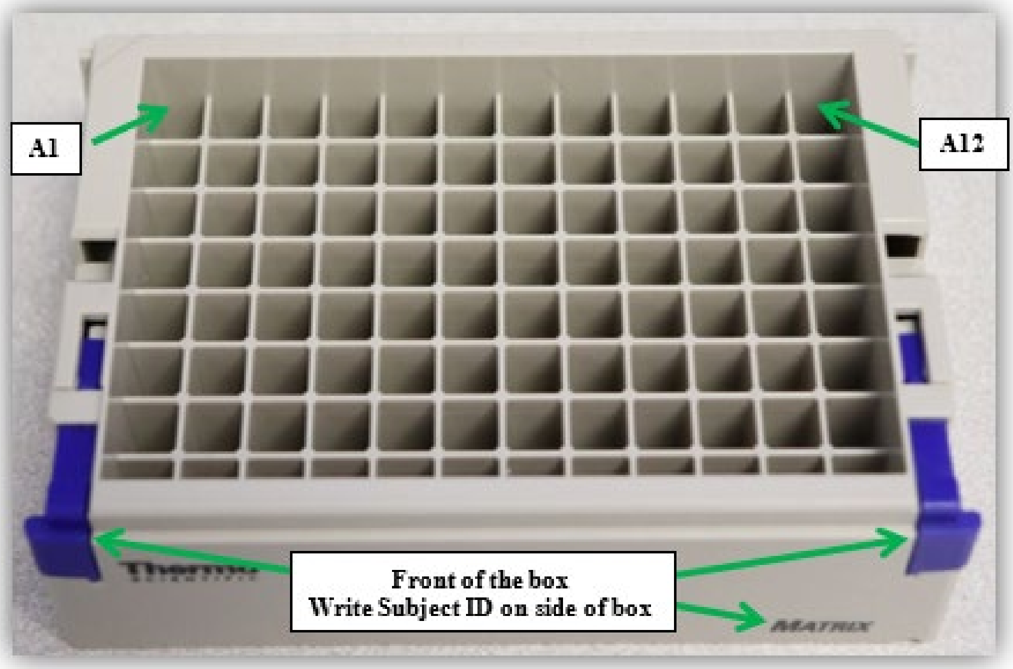

NOTE: Ensure the Subject ID is recorded on the side of the 96-well matrix box prior to freezing.

16. Record Freeze date and time in REDCap (see image above in Step 13).
17. See the Frozen Matrix Tube Packaging Section.

3.3 CSF COLLECTION

1. Perform the CSF collection from a cisternal tap, consistent with standard autopsy collection practices.
2. Using a pipette, transfer 1000µL into each of the 1.4 mL matrix tubes. Aliquot directly into the 1.4 mL matrix tubes matrix tubes (maximum of 10 aliquots). **Do not add more than 1000µL to the matrix tube.**

NOTE: If the quantity of CSF is less than 3 mL, divide the CSF evenly between three 1.4 mL matrix tubes; otherwise, aliquot 1000µL per matrix tube.

NOTE (2): If not all aliquots were obtained, leave all unused fields blank and dispose of any unused aliquot tubes. Do not send empty aliquot tubes to the PBC.

3. In REDCap, scan the matrix aliquot IDs into the CSF aliquot ID fields and record the corresponding aliquot volume (in microliters) and freeze time. **This step is key to properly link the aliquots to the Subject ID and to capture the associated specimen data.**

CSF Collection and Processing

CSF 'Please enter the volume (in microliter) and freeze date time associated with each CSF Aliquot'			
CSF Aliquot 1 ID	0228946297	CSF Aliquot 1 Volume (µl)	1000
		CSF Aliquot 1 Freeze Date/Time	07-26-2022 15:33 M-D-Y H:M
CSF Aliquot 2 ID	0228946589	CSF Aliquot 2 Volume (µl)	1000
		CSF Aliquot 2 Freeze Date/Time	07-26-2022 15:33 M-D-Y H:M
CSF Aliquot 3 ID	0228939420	CSF Aliquot 3 Volume (µl)	1000
		CSF Aliquot 3 Freeze Date/Time	07-26-2022 15:33 M-D-Y H:M
CSF Aliquot 4 ID	2158960304	CSF Aliquot 4 Volume (µl)	1000
		CSF Aliquot 4 Freeze Date/Time	07-26-2022 15:33 M-D-Y H:M
CSF Aliquot 5 ID	2480063047	CSF Aliquot 5 Volume (µl)	1000
		CSF Aliquot 5 Freeze Date/Time	07-26-2022 15:33 M-D-Y H:M

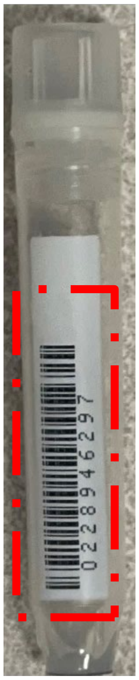

4. Record the volume of each aliquot, round and record the final volume of the aliquot that is created down to the nearest of 250µL. If the use of P1000 is not possible, use the disposable pipette. In the case of using the disposable pipette, the Trained staff must round the approximate final volume of the aliquot to the nearest 250µL increment (i.e., ½ full = 500µL, and full = 1000µL). **Do not add more than 1000µL to the matrix tube**. (See images below)

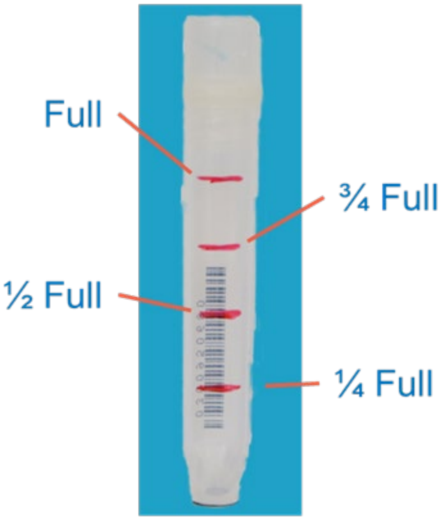

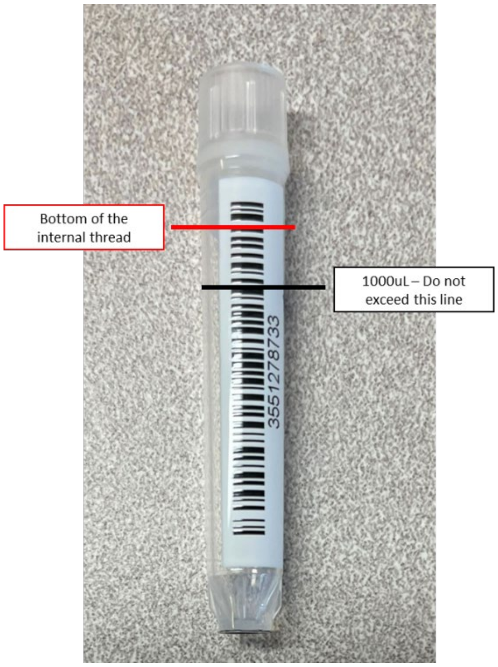

5. Store CSF matrix tubes in **Row C** of the 96-well matrix tube storage box at frozen temperatures (-80°C) until specimens are ready to be shipped to the PBC.

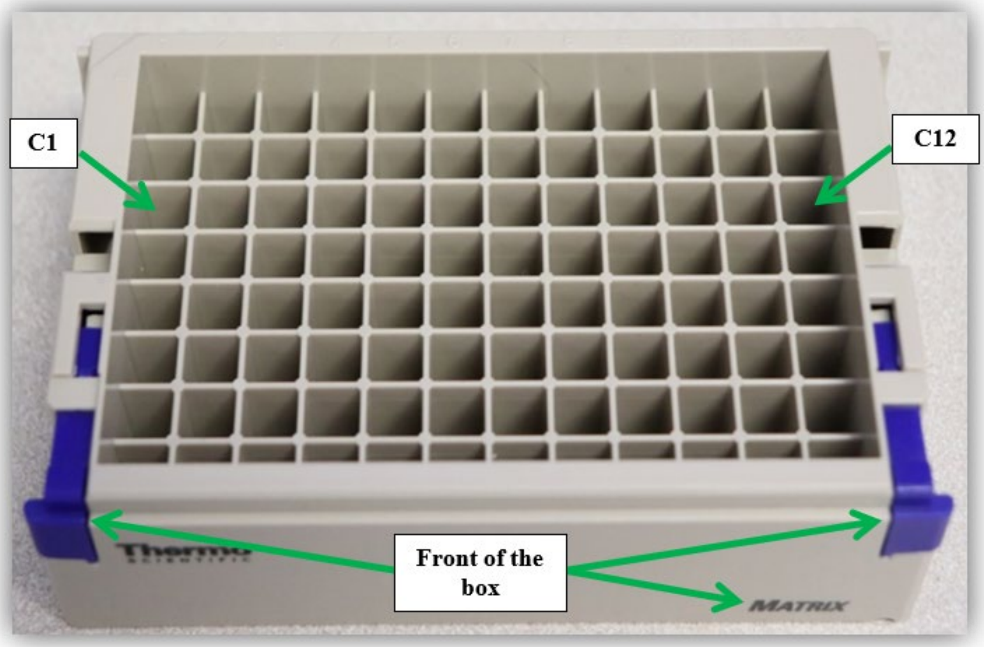

NOTE: Ensure the Subject ID is recorded on the side of the 96-well matrix box prior to freezing

6. Record Freeze date and time in REDCap
7. See the Frozen Matrix Tube Packaging Section.

3.4 BLOOD SPOT CARD COLLECTION

1. Record the RECOVER Subject ID in the provided space on the specimen label located on the flap of the card.
2. Apply a few drops of blood onto each circle on the blood spot card (image below).
3. Allow card to dry completely before closing the attached cover flap.
4. Seal dried blood spot card in a 4 x 6 biohazard bag with absorbant sheet at ambient temperatures until ready for shipping.

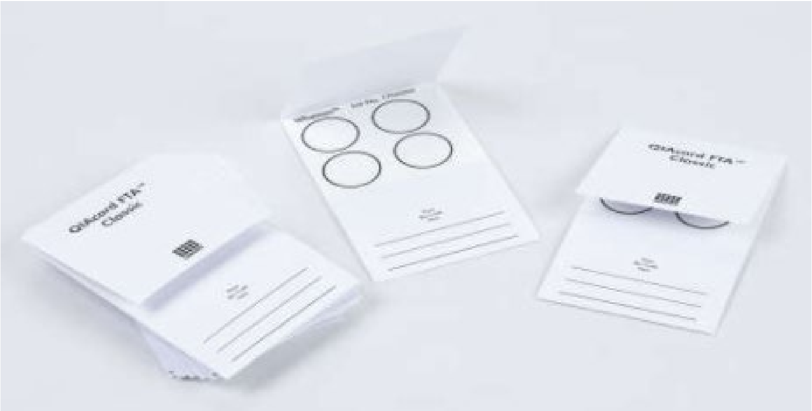

3.5 BRONCHIAL SWAB COLLECTION

1. Retrieve the bronchial swab transport media tube and the CLASSIQSwabs Plastic-Swab Bronchial Swab from the kit.
2. Record the RECOVER Subject ID for the decedent on defined space on the specimen tube label using a waterproof pen.
3. Using the CLASSIQSwabs Plastic-Swab Bronchial Swab, sample the left and right primary bronchi.
4. Gently rub and roll the swab. Leave swab in place for several seconds to absorb secretions.
5. Slowly remove swab while rotating it.
6. Aseptically remove the cap from the 3.0 mL MicroTest^{TM} M4RT Bronchial Transport vial labeled for bronchial swab.
7. Insert the swab into the media.
8. Bend the swab one direction to score the shaft.
9. Bend in the opposite direction to break the swab shaft evenly. Use a pair of sterile scissors if additional trimming is needed.
10. Replace the cap to the vial and close tightly.
11. Place the bronchial swab in Mailable Biospecimen Bag with absorbant sheet.
12. Seal the biohazard bag and store at frozen temperatures (-80°C) until ready for shipping.

3.6 STOOL COLLECTION

1. Retrieve the 25 mL brown cap stool container from the kit.
2. Record the RECOVER subject ID for the decedent on defined space on the specimen tube label using a waterproof pen.
3. Obtain stool specimen from the colon/rectum using the spoon attached to the 25 mL brown cap container.

4. Place the cap onto the tube. Be sure to firmly screw the container cap on the container.
5. Remove the parafilm from the paper backing and stretch it around the top of the stool container.
6. Seal in a 6 x 9 biohazard bag with absorbant at frozen temperatures (-80°C) until ready for shipping.

3.7 TISSUE COLLECTION

Tissue will be collected from 52 defined sites, with the option to submit an additional 4 undefined sites. The nerve sections have special size requirements (up to 3 mm) and are to be embedded on end.

- Tissue collected for the FFPE tissue cassettes should not exceed 2.0 x 2.0 x 0.4 cm.
- Tissue snap frozen for the Cryosette™ should not exceed 17 mm in diameter, and should not exceed a thickness of 4 mm.

NOTE: For any transplanted tissue, do not use the assigned number from the Tissue Blocks section in REDCap. Instead, use one of the additional tissue fields, designations 52 through 55.

In REDCap, ensure a selection is made for every tissue type. The boxes selected need to accurately reflect the tissue that was collected. Do not send any empty tissue collection containers to the PBC.

Defined tissues #19 (Cardiac Lesional tissue), #27 (Lung Lesional Tissue), and #34 (Renal Lesional tissue), require additional data entry in REDCap. (Image below)

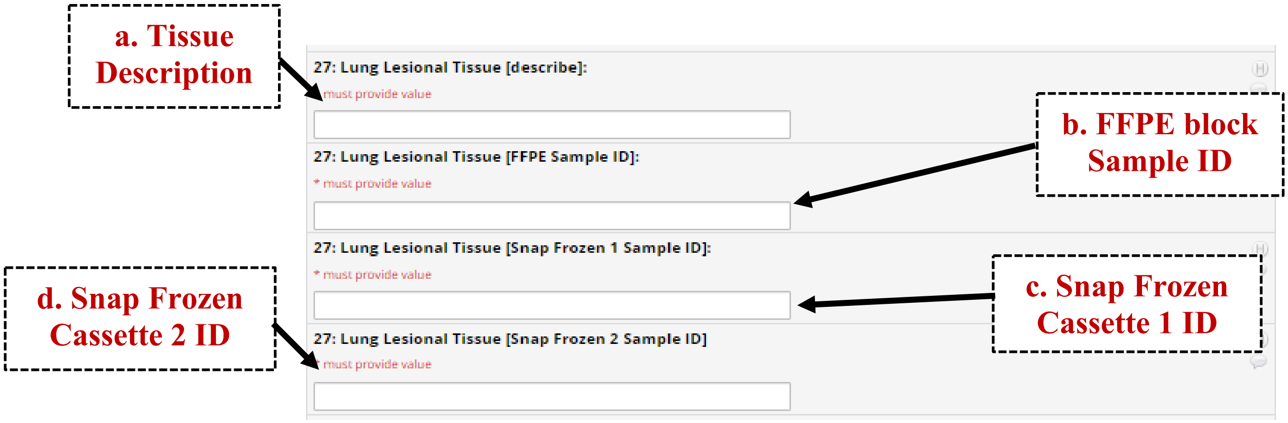

a. Provide a description of the tissue collected for the anatomical site
b. Enter the sample ID that is etched on the FFPE block into this field if collected.
c. Enter/Scan the sample ID that is printed on the frozen tissue cassette if collected.
d. Enter /Scan the sample ID that is printed on the other frozen tissue cassette if collected.

Undefined tissues fields #52-55 will require the same information if additional tissue sections are submitted.

NOTE: When the respective checkboxes are selected for defined tissues #19, #27, #34, and undefined tissues #52-55, the comment/ sample ID boxes will populate towards the bottom of the form. This is where the descriptions and IDs for these tissues should be entered (see second image below).

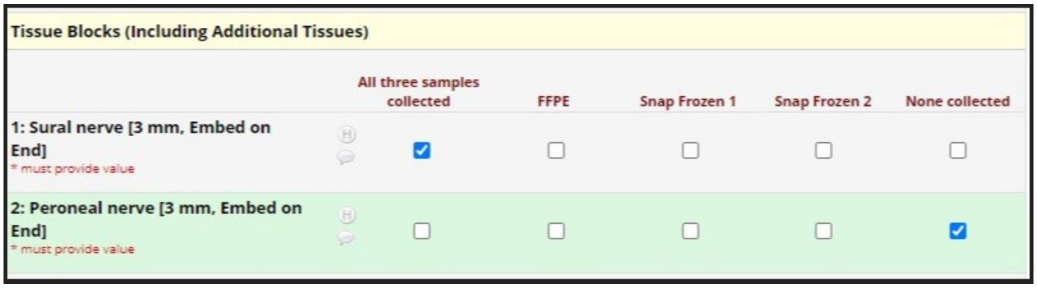

Tissue Blocks (Including Additional Tissues)	All three samples collected	FFPE	Snap Frozen 1	Snap Frozen 2	None collected
1: Sural nerve [3 mm, Embed on End] * must provide value	☑	☐	☐	☐	☐
2: Peroneal nerve [3 mm, Embed on End] * must provide value	☐	☐	☐	☐	☑

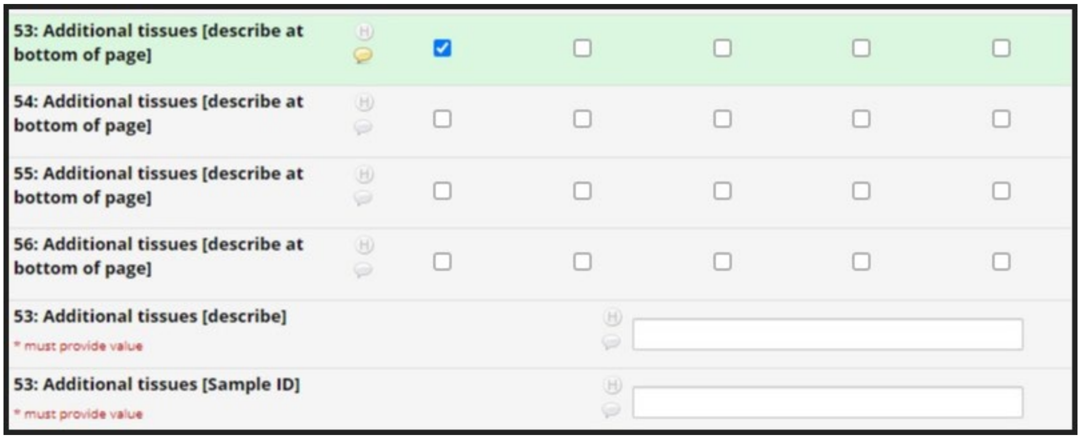

53: Additional tissues [describe at bottom of page]	☑	☐	☐	☐	☐
54: Additional tissues [describe at bottom of page]	☐	☐	☐	☐	☐
55: Additional tissues [describe at bottom of page]	☐	☐	☐	☐	☐
56: Additional tissues [describe at bottom of page]	☐	☐	☐	☐	☐
53: Additional tissues [describe] * must provide value					
53: Additional tissues [Sample ID] * must provide value					

3.7.1 FORMALIN FIXED PARAFFIN EMBEDDED (FFPE):
One tissue per anatomic site will be collected and fixed in 10% buffered formalin for between 16 and 32 hours, followed by processing and paraffin embedding using the provided pre-labeled tissue cassettes. Each anatomic location has a specific number designation, as listed in SOP-021.03 RECOVER Autopsy Specimen Key (Appendix D).

NOTE (1): All tissue types outlined in SOP-021.03 RECOVER Autopsy Specimen Key (Appendix D) are to have 1 FFPE block and 2 snap frozen blocks.
NOTE (2): If there is limited amount or difficulty obtaining any of the tissue types listed, the priority will be for the FFPE tissue**.**
NOTE (3): KIT-IDs are required on all FFPE cassettes. Sites should hand write the participant's KIT-ID on the cassettes using a StatMark™ pen if not already pre-labeled (see image below).

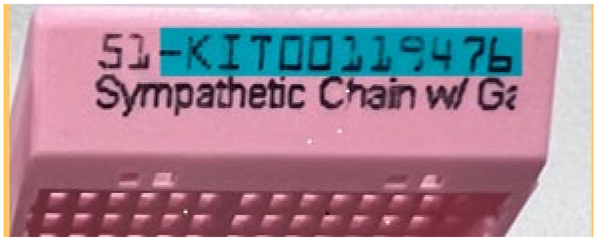

3.7.2 SNAP FROZEN:
Tissue sections (in duplicate) per anatomic site will be collected and snap frozen with dry ice using recommendation below. Each anatomic site has a specific number designation. as listed in SOP-021.02 RECOVER Autopsy Specimen Key (Appendix D)

NOTE: All tissue types outlined in SOP-021.03 RECOVER Autopsy Specimen Key (Appendix D) are to have 1 FFPE block and 2 snap frozen blocks.

NOTE (2): If there is limited of any tissue type listed, the priority will be for the FFPE tissue.
NOTE (3): KIT-IDs are required on all snap frozen Cryosettes™. Sites should hand write the participant's KIT-ID on the Cryosettes™ using a StatMark™ pen if not already pre-labeled (see image below).

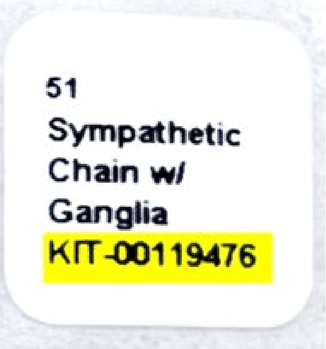

PBC RECOMMENDATION:

1. Place two inches of dry ice pellets at the bottom of the Ice Pan or Styrofoam cooler.
2. Place the thermal tray on top of the layer of dry ice.
3. The tissue can now be placed on top of the thermal tray using one of two methods (see image):
   - **A.** In closed Cryosettes™ placed on top of the thermal tray, or
   - **B.** On a piece of aluminium foil on top of the thermal tray, with the corresponding Cryosette™
     NOTE: The Cryosette™ must be chilled as well prior to placing the frozen tissue inside

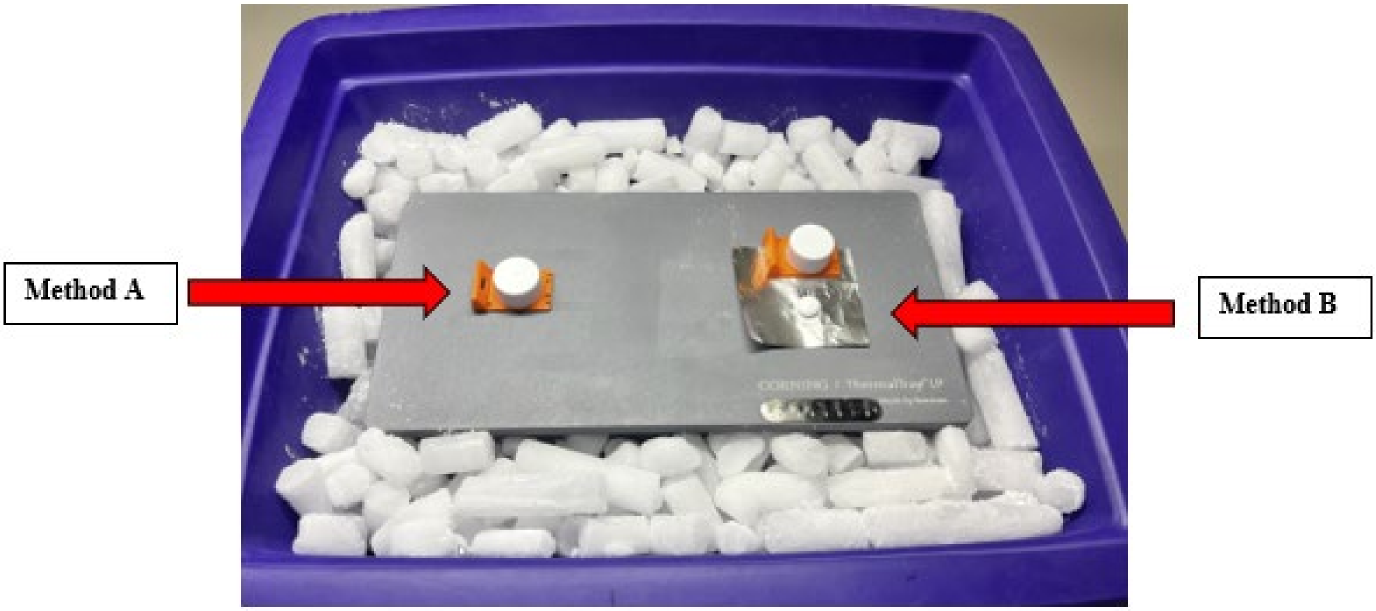

4. Allow tissue to freeze completely.
   - **A.** If Method B above was used, place tissue into corresponding chilled Cryosette™.

ALTERNATIVE RECOMMENDATIONS:

1. Freeze tissue in closed Cryosettes™ in sealed plastic bags in pellets of dry ice.
2. Freeze tissue in closed Cryosettes™ in sealed plastic bags in liquid nitrogen.
3. Freeze tissue on aluminum foil directly on dry ice pellets or slabs (no thermal tray).

4. Freeze tissue closed Cryosettes™ on thermal tray using liquid nitrogen.
5. Freeze tissue in closed Cryosettes™ in liquid nitrogen vapor phase.
6. Freeze tissue in closed Cryosettes™ directly on slabs of dry ice.

4.0 PACKAGING & SHIPPING

Procedural Notes:

- **DO NOT COMBINE MULTIPLE AUTOPSY CASES (DECENDENTS) IN ONE TEMPERATURE SPECIFIC SHIPPER.**
- Do not send any **empty** biospecimen collection containers to the PBC.
- Please confirm with shipping authorities that the FedEx Express pickup location accepts UN3373 Biological Substances, Category B.
- When packing the FFPE blocks and the Cryosettes™, package them in sequential order into the FFPE Block Transport Containers and Cryosette™ 21-count racks, respectively.
- Be sure to make at least 4 copies of the Requisition Form. One will be needed for each temperature specific shipper, and for the brain (if applicable). Sites should retain a copy for their records as well.
- **REDCap data entry must be complete, and Biospecimen form saved as Complete before shippers are handed off to carrier.**
- **Shippers should only be sent Mondays, Tuesdays, or Wednesdays.**
- **On the day a case is sent out to the PBC, send an email to the RECOVERPBC@mayo.edu inbox with the Subject ID(s) and the respective FedEx tracking numbers of the frozen and refrigerate shippers.**

4.1 FROZEN MATRIX TUBE INITIAL PACKAGING

1. See image below for how to properly store the serum matrix tubes and CSF matrix tubes in the 96-well matrix storage box.
   NOTE: Serum aliquots should be place in **Row A** and CSF aliquots should be placed in **Row C**.

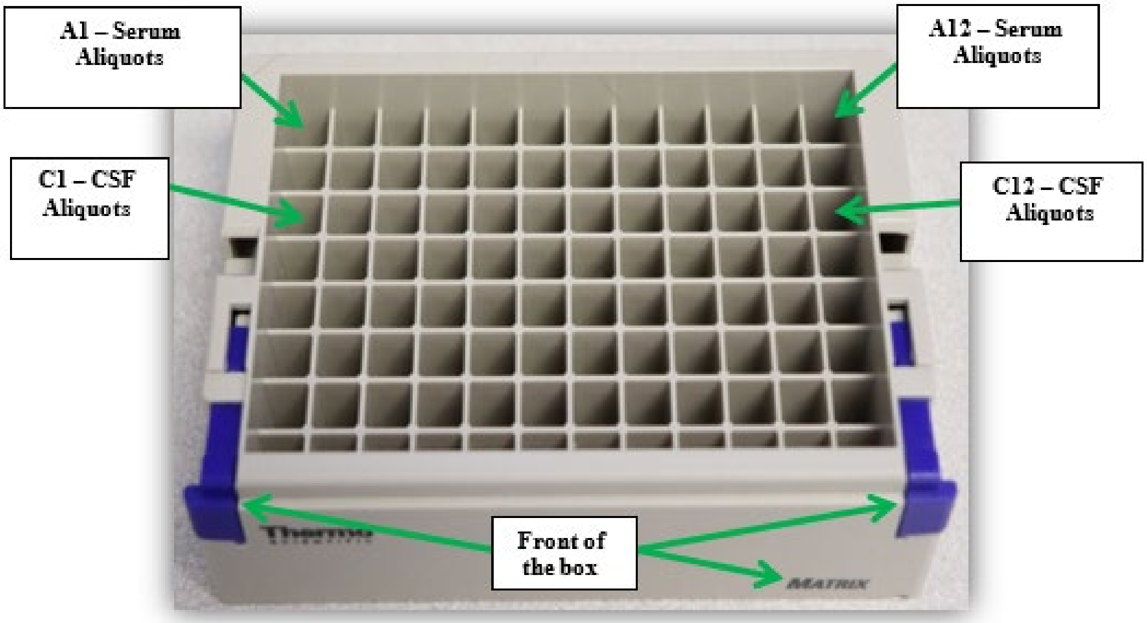

2. Place the closed 96-well matrix storage box into a 6 x 9 biohazard bag with an absorbant sheet and seal.
3. While in the REDCap form, use the Actions dropdown at the top of the form, select the "This data entry form with saved data (via browser's Save as PDF)" to print the completed form with all of the serum and CSF aliquot information (see image below).

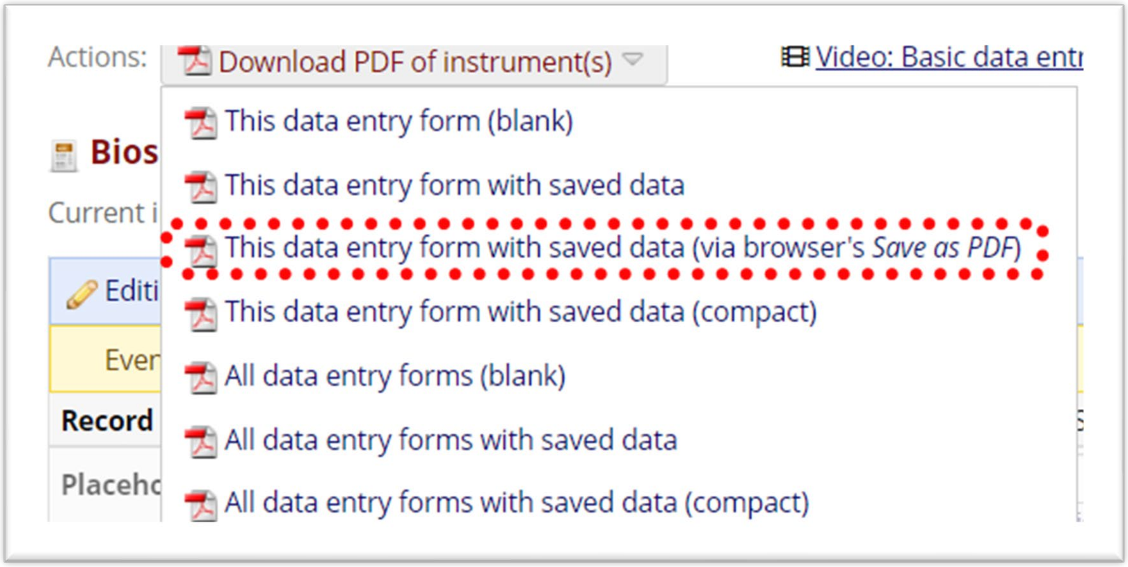

4. Print 2 copies of the completed PDF. This will serve as a manifest for the frozen matrix tubes, and as a QC check for the FFPE and Cryosette manifests.
5. **Enclose the REDCap Biospecimen PDF along with a copy of the completed requisition form** in a manila envelope (not provided) to be included in the frozen shipper.

NOTE (1): This PDF should resemble the REDCap screen. If it does not, ensure the correct option is selected from the Actions dropdown menu.

NOTE (2): Ensure all *required fields on the requisition form have been completed prior to printing.

4.2 FFPE AND CRYOSETTE™ INITIAL PACKAGING

1. Place the FFPE blocks into the provided FFPE Block Transport Containers in sequential order and use the appropriate checkboxes on the requisition form to signify if tissue was present or absent. This should be consistent with the data entered in REDCap.

NOTE: If any discrepancies are noted during this QC check, ensure the REDCap form is updated to accurately reflect what will be sent.

2. Place the closed FFPE Block Transport Containers into the 12 x 15 biohazard bag with an WyPall® and seal.
3. Store at ambient temperatures until ready for shipping.
4. Ensure all the Cryosette™ 21-count Racks (6) sides are labeled with the RECOVER Subject ID using a StatMark™ Pen (see image below).
   Place the snap frozen Cryosette™ into the Cryosette™ 21 count racks in sequential order (see images on next page) and use the appropriate checkboxes on the requisition form to signify if tissue was present or absent. This should be consistent with the data entered in REDCap.
   NOTE: If any discrepancies are noted during this QC check, ensure the REDCap form is updated to accurately reflect what will be sent.

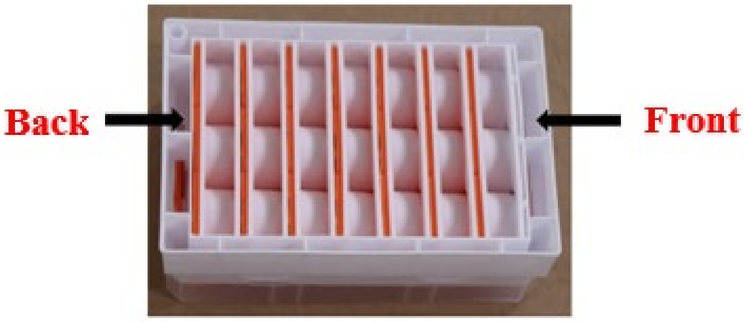

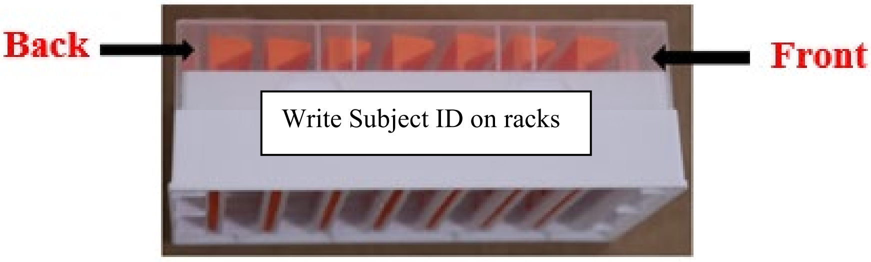

5. Stack the closed Cryosette™ 21 count racks into a 12 x 15 biohazard bag with a WyPall® and seal.
6. Store at frozen temperatures (-80°C) until ready for shipping.

4.3 FINAL PACKAGING AND SHIPPING

4.3.1 Shipping Frozen Specimens

1. Retrieve all frozen RECOVER specimens for **one decedent** to prepare for final packaging into the return shipper.
2. Obtain the RECOVER 20lb Frozen Autopsy Shipper.
3. Place two inches of dry ice covering the bottom of the Styrofoam shipper.
4. Cover with a single layer WyPalls®.

NOTE: Images for the best way to package the frozen return shipper are shown on the next page.

5. Place the 6 x 9 biohazard bag containing the 96-well matrix storage box on top of layer of dry ice and WyPalls®.
6. Place the sealed 12 x 15 biohazard bags with the Cryosette™ 21 Count Racks on top of the WyPalls®, so that each box has some contact with the dry ice and layer of WyPalls®.
7. Place the Mailable Tube Bio-Specimen Bags containing the bronchial swab into the shipper containing dry ice.
8. Place the sealed 6 x 9 biohazard bag that contains the stool sample into the shipper containing dry ice.
9. Cover the top of the biospecimens completely by filling the remaining space of Styrofoam shipping container with dry ice.
10. Place the lid on the Styrofoam shipper.

11. Enclose the REDCap Biospecimen PDF **and** the completed requisition form in a manila envelope (not provided in kit) on top of the Styrofoam lid, underneath the cardboard flap.

NOTE: The first page of the requisition form must always be included with shipment. One completed biospecimen manifest is required within each shipper – either the REDCap Biospecimen PDF hardcopy or the completed requisition form manifests (Cryosette™ Block Manifest and FFPE cassette Block Manifest) is acceptable.

12. Close and seal the cardboard shipper. Seal with packaging tape (not provided in kit).

NOTE: The box will come pre-labeled to meet DOT/IATA requirements. Ensure all markings are complete and not obstructed or damaged.

13. Ensure the REDCap form is saved as Complete before shippers are handed off to carrier.

14. Drop off the package at a FedEx Express pickup location that accepts hazardous materials. Packages should only be shipped **Monday – Wednesday** for arrival the PBC **Tuesday – Thursday**.

<table>
<tr>
<td>Properly sealed frozen specimens in their respective biohazard bags</td>
<td>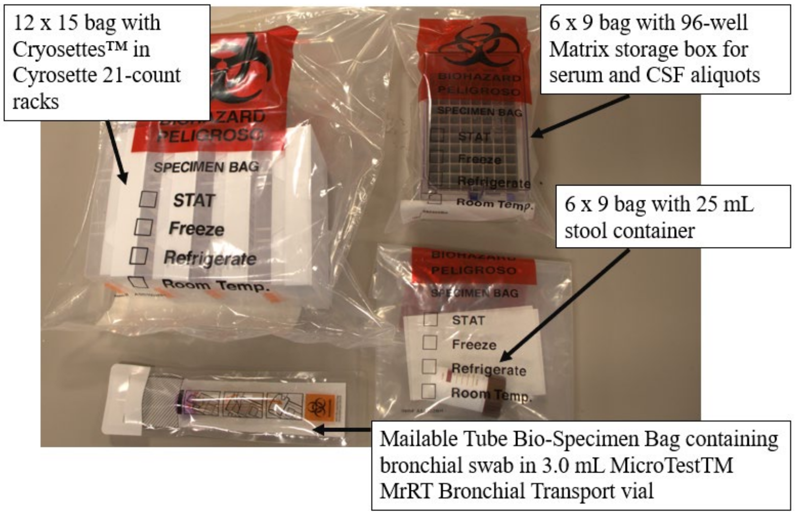
</td>
</tr>
<tr>
<td>Ideal packaging of frozen specimens in the RECOVER 20lb Frozen Autopsy Shipper
• Ensure there is 2 inches of dry ice and a layer of WyPalls® below the specimens
• Cover the top of the specimen bags with dry ice by filing all of the remaining space of the Styrofoam shipper
• Place manila envelope with REDCap Biospecimen PDF and requisition form between Styrofoam lid and cardboard box. Seal cardboard box with tape.</td>
<td>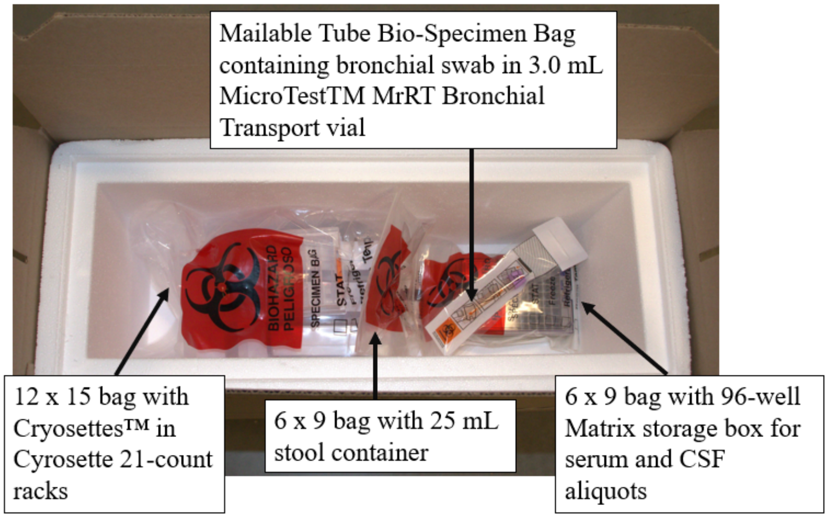
</td>
</tr>
</table>

Do not label over or remove any of the required shipping identifiers for dry ice or biological substances	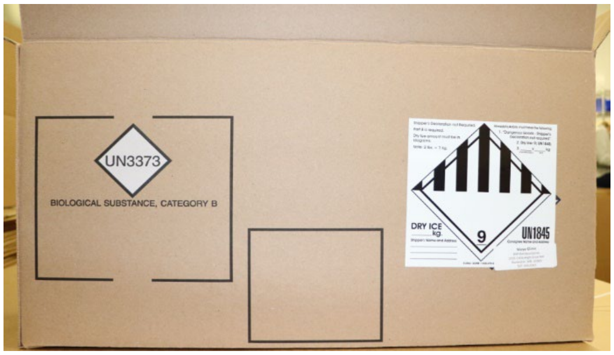

4.3.2 Shipping Refrigerate Specimens

1. Retrieve all refrigerate RECOVER specimens for **one decedent** to prepare for final packaging into the return shipper.
2. Obtain the RECOVER 10lb Refrigerate Autopsy Shipper.

NOTE: Images for the best way to package the refrigerate return shipper are shown below these steps.

3. Place the 12 x 15 biohazard bag containing the FFPE Block Transport Containers on top of the frozen cool pack.

NOTE: This cool pack should be frozen upon receipt of the RECOVER 10lb Refrigerate Autopsy Shipper. This cool pack is meant to keep the specimens at a refrigerate temperature during transit, particularly in the warmer months.

4. Place the sealed return biohazard bag containing the blood spot card on top of the sealed 12 x 15 biohazard bag containing the FFPE Block Transport Containers.

NOTE: Please send the FFPE blocks and dried blood spot card in one refrigerate shipper together.

5. Place the lid on the Styrofoam shipper.
6. Enclose the REDCap Biospecimen PDF **and** the completed requisition form in a manila envelope (not provided in kit) on top of the Styrofoam lid, underneath the cardboard flap.

NOTE: The first page of the requisition form must always be included with shipment. One completed biospecimen manifest is required within each shipper – either the REDCap Biospecimen PDF hardcopy or the completed requisition form manifests (Cryosette™ Block Manifest and FFPE cassette Block Manifest) is acceptable.

7. Close and seal the cardboard shipper. Seal with packaging tape (not provided in kit).

NOTE: The box will come pre-labeled to meet DOT/IATA requirements. Ensure all markings are complete and not obstructed or damaged.

8. **Ensure the REDCap form is saved and finalized before shippers are handed off to carrier.**
9. Drop off the package at a FedEx Express pickup location that accepts hazardous materials. This would not apply if using a scheduled FedEx pick-up. Packages should only be shipped **Monday – Wednesday** for arrival the PBC **Tuesday – Thursday**.

12 x 15 biohazard bag with FFPE Block Transport Containers (bag unsealed only for purposes of photograph)	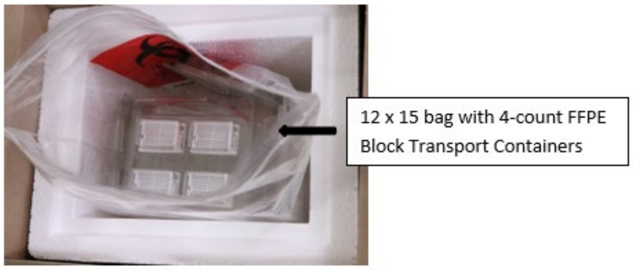
Ideal packaging of specimens in the RECOVER 10lb Refrigerate Autopsy Shipper<br>• Blood spot card and FFPE Block Transport Containers in biohazard bags on top of frozen cool pack<br>• Place manila envelope with REDCap Biospecimen PDF and requisition form between Styrofoam lid and cardboard box. Seal cardboard box with tape.	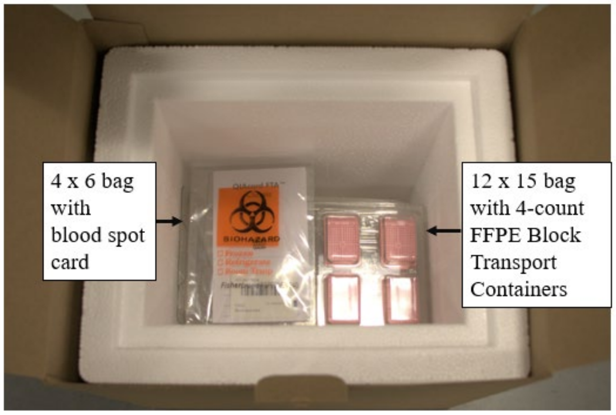

IMPORTANT REMINDER

Please ensure the REDCap Autopsy form is marked as Complete and Saved before sending any shippers to the PBC.

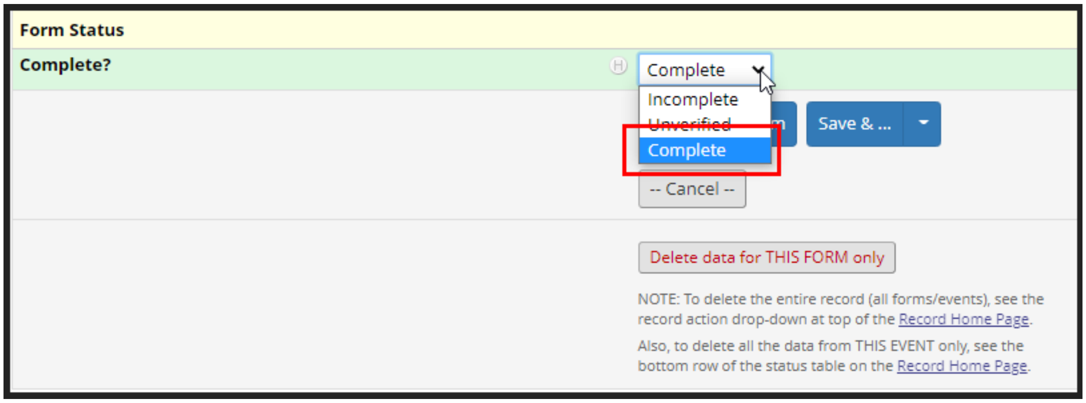

[RETURN TO TABLE OF CONTENTS](#)

III. APPENDICES

A. AUTOPSY KIT CONTENTS

MATERIALS (included in Kit)

- BD Vacutainer® 8.5 mL SST (2)
- 1.4 mL Matrix tubes (20)
- QIAcard FTA Classic (100) WB120205(1)
- CLASSIQSwabs Plastic-Swab Bronchial Swab (1)
- 3.0 mL MicroTest™ M4RT Transport vial (1)
- FFPE Tissue Cassettes (56)
- 4-Count FFPE Block Transport Container (14)
- Cryosettes™ (112)
- Cryosette™ 21-count Rack (6)
- 96-well Matrix Storage Box (1)
- 25 mL Stool Container with Spoon (1)
- Parafilm
- 6 x 9 Biohazard bags (2)
- 4 x 6 Biohazard bag (1)
- Mailable Biospecimen Bag for Bronchial Swab (1)
- 12 x 15 Return Biohazard bags (2)
- Requisition Form
- Checklist

MATERIALS (Not included in Kit)

- Tape
- Manila envelopes

RETURN SHIPPERS

RECOVER 20lb Frozen Autopsy Shipper	• 20 lb Styrofoam cooler with lid<br>• Cardboard sleeve with UN3373 and dry ice designations
RECOVER 10lb Refrigerate Autopsy Shipper<br><br>**NOTE (1): Freeze** the cool pack for the RECOVER 10lb Refrigerate Autopsy Shipper for at least 24 hours at a temperature of -10 to -20°C for shipping<br><br>• **Do not freeze** the cool pack at a temperature less than -20°C/-4°F<br>• **Do not freeze** the cool pack in a -80°C/-112°F freezer or on dry ice.<br><br>**NOTE (2):** Freezing the cool pack in a temperature below -20°C may cause freezing of the specimens.	• 10 lb Styrofoam cooler with lid<br>• Refrigerate cool packs<br>• Cardboard sleeve with UN3373 designations

RETURN TO TABLE OF CONTENTS

B. RECOVER COLLECTION LABELS AND COLLECTION TUBE GUIDE

NOTE: These are the temporary kit labels, as the long-term RECOVER label template is being configured. These will be changed when this solution is ready to go live.

Collection Tube	RECOVER Label	Collection Device Image
8.5 mL SST (Serum Separator Tube)	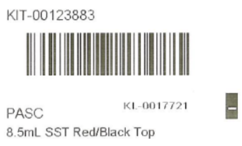	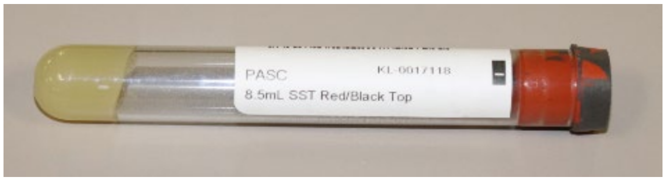
3.0 mL MicroTestTM M4RT Transport vial	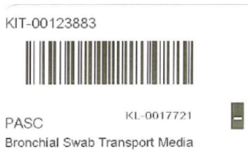	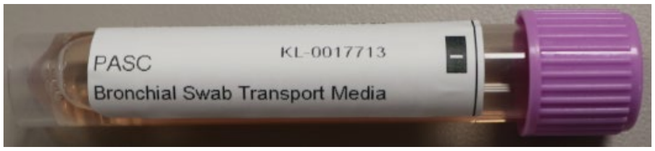
1.4 mL Matrix tubes	Pre-barcoded from manufacturer	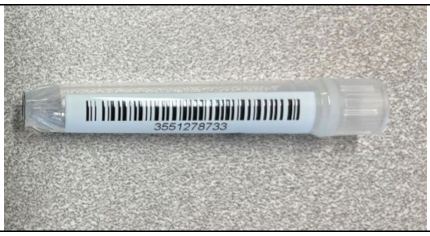
25mL Fecal Container with spoon	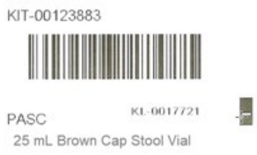	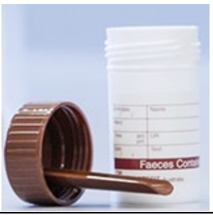
Tissue Cryosette™	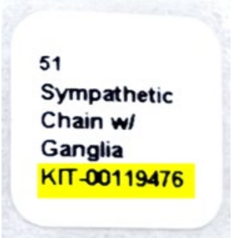 Pre-labeled with Tissue Block Designation number, anatomic site, and KIT-ID on bottom	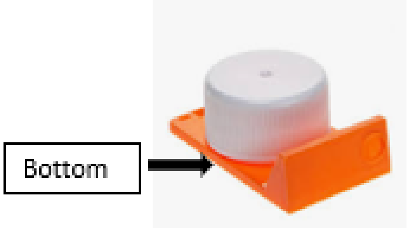
FFPE Tissue Cassette	Engraved with Tissue Block Designations number (1-56), KIT-ID, and anatomic site	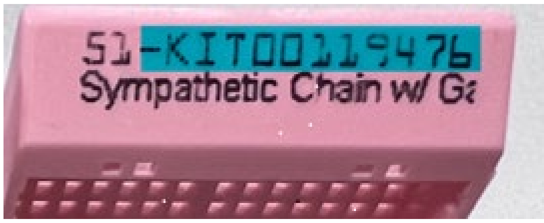

[RETURN TO TABLE OF CONTENTS](#)

C. G-001.03 RECOVER AUTOPSY TEMPLATE (LAMINATED)

Patient Name:	Subject ID:

EXTERNAL APPEARANCE
Date of External Appearance form collection:

Race:	[Black | Asian | White | Native American | Asian Pacific Islander | ***]
Sex:	[***]
Genitalia	[male | female | ambiguous | intersex]
Weight:	[***] kg
Height:	[***] cm
Hair:	[None | Balding | Short| Medium| Long| Black | Brown | Blonde |Grey | Other (specify)] [Curled? Yes or No]
Sclerae:	[Icteric | Anicteric]
Eyes:	[Normal | Abnormal (specify)]
Ears:	[Normal | Abnormal (specify)]
Nose:	[Normal | Abnormal]
Oral cavity:	[Good dentition | Poor dentition | Dentures | No teeth | Exam not performed due to rigor | Other (specify)]
Nails:	[Normal | Onychomycosis | Splinter hemorrhages | Cyanotic |Other (specify)]
Edema:	[None | Peripheral | Generalized |Other (specify)]
Skin:	[Scars| Incisions| Lesions (number, location, size) | Scars consistent with ECMO]
Evidence of therapy:	[None | NG tube | PEG tube | ET tube | Foley catheter| Rectal tube| IV catheters]
Back:	[No lesions| Decubitus ulcer, depth [***] cm

INCISIONS AND BODY CAVITIES

Date of Incisions and Body Cavities form:
Tissue Fixation time (hours):

Incision:	[Standard Y-shaped | Biparietal | ***]
Was the appendix surgically absent?	[Yes | No]
Was the gallbladder surgically absent?	[Yes | No]
Was the thymus absent?	[Yes | No |Indeterminate]
Any other organs surgically absent or partially removed:	Yes (which organ?)| No]
Organs in normal anatomic positions:	[Yes | No, describe]
Peritoneal fluid:	[None | *** ml | Serous | Cloudy | Serosanguinous | Sanguinous | Purulent]
Peritoneal surfaces:	[Smooth | Adhesions | Lesions]
Pleural fluid	<u>Right:</u> [None | *** ml | Serous | Serosanguinous | Sanguinous | Purulent]<br><br><u>Left:</u> [None | *** ml | Serous | Serosanguinous | Sanguinous | Purulent]
Pleural cavity:	<u>Right</u>: [Smooth | Adhesions | Lesions]<br><br><u>Left</u>: [Smooth | Adhesions | Lesions]
Cultures taken	[organ; location - result]

CARDIOVASCULAR SYSTEM
Date of Cardiovascular System form:

Aorta (Thoracic, Abdominal):	[No atherosclerosis | Mild | Moderate | Severe atherosclerosis]
Ascending Aorta:	Appropriate caliber? [Yes | No] Normal configuration? [Yes | No]

Venae cavae:	[Thin walled | Patent | Thrombi present]
Pulmonary artery:	[Normal | Contains embolus]
Pericardium:	[Intact | Adhesions (loose or tight) | Granular | Thickened]
Pericardial fluid:	[None | *** ml | Serous | Cloudy | Sanguinous |Sanguinous | Purulent]
Epicardium:	[Smooth and thin | Thickened]
Heart weight:	[***] g
LAD:	[None | Mild | Moderate | Severe Arthrosclerosis]<br><br>[LAD Stenosis present? (Yes or No) (Partial or Complete)]
LCX:	[None | Mild | Moderate | Severe Arthrosclerosis]<br><br>[LCX Stenosis present? (Yes or No) (Partial or Complete)]
RCA:	[None | Mild | Moderate | Severe Arthrosclerosis]<br><br>[RCA Stenosis present? (Yes or No) (Partial or Complete)]
Coronary circulation:	[Right dominant | Left dominant | Co-dominant]
Coronary ostia:	[Normally positioned? (Yes or No) | Patent? (Yes or No)]
Foramen ovale:	[Probe patent | Closed]
Chamber dilation:	[Yes (Right atrium, Left atrium, Right ventricle, Left Ventricle) | No]
Tricuspid circumference:	[***] cm REF RANGE: 10.0 - 12.5 cm
Tricuspid:	Abnormal Yes/No
Tricuspid valve leaflets/chordae:	Thickened [Yes (Mild, Moderate, Severe) | No]

Tricuspid valve:	Lesions [Yes (Number, location (free edge, body, annulus), size (cm) | No
Pulmonic:	Circumference: [***] cm REF RANGE: 7.0 - 9.0 cm
Pulmonic abnormalities:	[Yes | No]
Pulmonic valve leaflets/chordae:	Thickened [Yes (Mild, Moderate, Severe) | No]
Pulmonic valve:	Lesions [Yes (Number, location (free edge, body, annulus), size (cm) | No
Mitral:	Circumference: [***] cm REF RANGE: 8.0 - 10.5 cm
Mitral:	Abnormal Yes/No
Mitral valve leaflets/chordae:	Thickened [Yes (Mild, Moderate, Severe) | No]
Mitral valve:	Lesions [Yes (Number, location (free edge, body, annulus), size (cm) | No
Aortic:	Circumference: [***] cm REF RANGE: 6.0 - 7.5 cm
Aortic:	Abnormal Yes/No
Aortic valve leaflets/chordae:	Thickened [Yes (Mild, Moderate, Severe) | No]
Aortic valve:	Lesions [Yes (Number, location (free edge, body, annulus), size (cm) | No
Left ventricular free wall:	[***] cm NORMAL: Less than 1.5 cm
Septum:	[***] cm NORMAL: Less than 1.5 cm
Right ventricular free wall:	[***] cm NORMAL: Less than 0.5 cm
Myocardium:	[Normal firm, red-brown | Abnormal]
Myocardium:	Lesions [Yes (Number, location (right, left, septum, anterior, posterior, lateral free wall), size (cm) | No

RESPIRATORY SYSTEM
Date of Respiratory System form:

Epiglottis, larynx, trachea:	[Lesions (number) | No lesions] [Location of lesion: Epiglottis | Larynx | Trachea] [Location (specify): Superior | Inferior] [Location (specify): Right | Left] [Size of lesion (centimeters):]
Right lung weight:	[***] g, REF RANGE: 360 - 570 g
Left lung weight:	[***] g, REF RANGE: 325 - 480 g
Fixation:	[Fixed in distention | Cut fresh | Other (specify)]
Right lung parenchyma:	[Soft and pale red | Consolidated| Cavitary lesions, or abscesses | Hemorrhagic/Infarcted | Fibrotic, |Emphysematous]
Left lung parenchyma:	[Soft and pale red | Consolidated| Cavitary lesions, or abscesses | Hemorrhagic/Infarcted | Fibrotic, |Emphysematous]
Bronchi:	[Lesions (number) | No Lesions | Obstructed? describe] [Lesion Location: Right | Left]<br><br>[Location specify: Branch | Mainstem] [Size (cm)]
Vasculature:	[Pulmonary emboli, describe location | Atherosclerosis, describe location]

DIGESTIVE SYSTEM
Date of Digestive System form:

Tongue:	[Papillated | Smooth]
Esophagus:	[Normal anatomic configuration | Abnormal anatomic configuration]
Squamocolumnar junction:	[Sharply defined | Indistinct]

Stomach:	[Empty | Distended | Contains partially digested food and liquids | Mucosal lesions | No lesions,<br><br>Lesion number, location [Antrum | Body | Fundus], size (cm)<br><br>[Stomach contents: Empty | Contains partially digested foods and liquids]
Superior mesenteric artery:	[No | Minimal | Mild | Moderate | Severe atherosclerosis| Patent | Not patent]
Liver weight	***] g REF RANGE: 1500 - 1800 g
Liver	[Smooth and glistening (Yes or No) | Slightly firm | Firm | Soft | Nodular (Yes or No), if yes, Micro or Macro | Lesions (number, location [Right lobe | Left Lobe | Caudate], size (cm) | Maroon-brown | Green tinged | Yellow orange | Mottled red | Nutmeg liver appearance (Yes or No)]
Pancreas dimensions:	*** , give 3 dimensions] cm Length/Width/Depth
Pancreas:	[Tan | Firm and lobulated | Autolyzed| Lesions (number)]
Pancreatic/Common bile duct	[Probe patent | Not probed patent]
Gallbladder:	[Present | Surgically absent| <br>[Gallbladder wall: Thin | Thick | Fibrous]<br><br>[Gallbladder Mucosa: Velvety | Trabeculated | Necrotic]<br><br>[Gallbladder mucosa color: Green | Yellow | Brown]<br><br>[Gallbladder calculi: Yes | No] [Gallstones: Yes | No] [Color of stones: Black | Green | Yellow]<br><br>[Gallbladder Contents – Dark green mucoid bile: Yes | No]

DIGESTIVE SYSTEM (Bowel Examination)
Date of Digestive System Bowel Examination form:

Appendix:	[Present | Surgically absent | Lesions? (number, Location - Tip | Mid-portion | Base] [Size (cm)]

Small bowel:	[Usual caliber | Dilated | Stricture | Tan/pink shiny serosa | Red/brown dusky serosa | Serosal adhesions | Tan mucosa | Polyps | Diverticula | Lesions (number, location [Duodenum | Jejunum | Ileum], size (cm)]<br><br>[Adhesions? Adhesions | No adhesions]
Large bowel:	[Usual caliber | Dilated | Stricture | Tan/pink shiny serosa | Red/brown dusky serosa | Serosal adhesions | Tan mucosa | Polyps | Diverticula | Lesions (number, location (Cecum| Ascending | Transverse | Descending | Sigmoid | Rectum), size (cm)

URINARY SYSTEM
Date of Urinary System form:

Bladder:	[Collapsed | *** ml of urine | congested mucosa | Trabeculated | Lesions (number, location (dome, right lateral wall, left lateral wall, bladder neck)]
Ureters:	[Patent | Not patent | Dilated | Not dilated]
Renal arteries:	[No | Mild | Moderate | Severe Atherosclerosis| Patent]
Right kidney weight:	[***] g
Right kidney:	[Smooth | Granular and pitted |Scarred | Clearly- or Ill-defined corticomedullary junctions | Red-brown (Yes or No) | Parenchyma lesions]
Left kidney weight:	[***] g
Left kidney:	[Smooth | Granular and pitted |Scarred | Clearly- or Ill-defined corticomedullary junctions | Red-brown (Yes or No) | Parenchyma lesions]

GENITAL SYSTEM – MALE/FEMALE

Prostate (3 dimensions/describe):	[*** x *** x ***cm| Normal | Enlarged| Nodular]
Testes:	[Normal size | Enlarged | Nodular | Brown parenchyma | Tubules string in normal manner | Tubules do not string | Testes cut surface lesions]

Right ovary	[Not identified | Size (cm) *** x *** x ***cm]
Left ovary	[Not identified | Size (cm) *** x *** x ***cm]
Left fallopian tube	[Normal | Hemorrhagic | Edematous]
Right fallopian tube	[Normal | Hemorrhagic | Edematous]
Uterus	[Normal | Enlarged | Atrophic]
Endometrium	[Pale | Red | Thickened]
Vagina	Without lesion | Other
Cervix	Without lesion | Other

ENDOCRINE SYSTEM
Date of Endocrine System form:

Right adrenal weight:	[***] g AVERAGE: 6 g (trimmed)
Left adrenal weight:	[***] g AVERAGE: 6 g (trimmed)
Adrenal parenchyma:	[Uniform yellow cortices | Good demarcation from the medullae | Autolyzed]
Thyroid weight:	[***] g, REF RANGE: 30-70 g
Thyroid parenchyma:	[Symmetric | Red-brown | Firm | Nodular | Lesions (number, location [Right lobe | left lobe | isthmus | pyramidal lobe], specify location [superior | middle | inferior], size (cm)]
Thymus	Hypertrophic (Yes or No)

LYMPHORETICULAR SYSTEM
Date of Lymphoreticular System form:

Spleen weight:	[***] g REF RANGE: 150 - 200 g unless over 80 years old, then AVERAGE 100 g
Spleen:	[Smooth and intact | Dark red | firm or soft | lesions, (number, location [capsule | parenchyma], size)]
Bone marrow:	[Yes (dark red) or No | Hard | Softer than usual | lesions, (number, location [name of bone], size)]
Bone marrow squeeze:	[Inadequate (less than 0.25 cc) | Adequate (0.25 cc or more)]
Lymph nodes:	[Not enlarged | Lymphadenopathy, describe location]

MUSCULOSKELETAL SYSTEM
Date of Musculoskeletal System form:

Ribs:	[Fractures (specify location) | No fractures]
Diaphragm:	[Normal | Abnormal | lesions (number, location [thoracic | peritoneal surface], size (cm)]
Skeletal muscles:	[Red-brown and firm | Appropriate mass for age/gender | Atrophied]
Vertebral column:	[Normal curvature | Kyphosis | Scoliosis]

CENTRAL NERVOUS SYSTEM
Date of Central Nervous System form:

Brain weight:	[***] g, REF RANGE: 1100 - 1700 g
Dura:	[Epidural hemorrhage | Subdural hemorrhage | Dural sinus thrombus | Bony abnormalities | Lesions Y/N]
Cerebral hemispheres:	[Herniation | Asymmetric | Atrophy (Global, Frontal, Parietal, Temporal, Occipital) | Edema | Lesions | Normal development or abnormal development |
Hemorrhage:	[None | Epidural | Subdural | Subarachnoid | Other (specify)]
Circle of Willis:	[Normal | Atherosclerosis (mild, moderate, severe) | Aneurysm] (location]
Cerebellum:	[Tonsillar Herniation | Atrophy | Normal development | Abnormal development | Lesions]
Spinal Cord:	[Lesions | Length *** cm from cut superior to cornus]
Brain stem:	[Normal development | Abnormal development | Hemorrhage (Yes or No)]
Substantia nigra:	[Normally pigmented?] (If no, pallor – mild, moderate or severe)

Locus ceruleus	[Normally pigmented?] (If no, pallor – mild, moderate or severe)

RETURN TO TABLE OF CONTENTS

D. SOP-021.03 RECOVER AUTOPSY SPECIMEN KEY (Laminated)

2.0 TISSUE STANDARDS

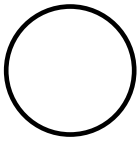

Cryosette™ size guideline

- For the FFPE tissue blocks, tissue should not exceed 2.0 x 2.0 x 0.4 cm.
- For the snap frozen tissue for the Cryosette™, the tissue should not exceed 17 mm in diameter, and should not exceed a thickness of 4 mm.
- The sural nerve and peroneal nerve sections can be up to 3 mm in length.

Tissues #19, #27, #34 require additional data entry in REDCap in the corresponding data fields. Fields #52 through #55 will require the same if additional tissue submitted.

NOTE: For any transplanted tissue, do not use the assigned number below in REDCap. Instead, use one of the additional tissue fields, designations 52 through 55.

3.0 BLOCK SUMMARY BY TISSUE TYPE

1	Sural nerve [Up to3 mm, **Embed on End**]		28	Spleen
2	Peroneal nerve [Up to 3 mm, **Embed on End**]		29	Bone Marrow (rib squeeze)
3	Skin, Calf including subcutis, 5 mm punch		30	Pancreas
4	Muscle, Gastrocnemius		31	Liver, right lobe (central)
5	Muscle, Psoas		32	Mesenteric fat
6	Duodenum		33	Kidney, right or left
7	Ileum		34	Renal Lesional tissue [**describe**]
8	Colon, right		35	Testis
9	Colon, descending/sigmoid		36	Ovary
10	Adrenal		37	Fallopian Tube
11	Aorta, Ascending		38	Olfactory bulb
12	Right Ventricle, Posterior		39	Frontal cortex with leptomeninges
13	Left Ventricle, Lateral		40	Basal ganglia
14	Left Ventricle, Basal		41	Thalamus
15	Right Atrium/SVC (near SA node)		42	Hippocampus
16	Left Atrium		43	Occipital cortex
17	Coronary artery, right		44	Amygdala
18	Coronary artery, left		45	Pons
19	Cardiac Lesional tissue [**describe**]		46	Choroid plexus
20	Hilar/Mediastinal Lymph nodes		47	Medulla (area postrema)
21	Trachea		48	Cerebellum with dentate
22	Right upper lobe, with pleura		49	Dura with sinus
23	Right middle lobe		50	Spinal cord (thorax)
24	Right lower lobe		51	Sympathetic chain with ganglia
25	Left upper lobe, with pleura		56	Dorsal Root ganglia
26	Left lower lobe			
27	Lung Lesional Tissue [**describe**]		52-55	Additional tissues [**describe**] *Any transplanted tissue should be noted in these additional sections, not using the designated numbers listed above in this block summary.

RETURN TO TABLE OF CONTENTS

E. CL-002.04 RECOVER AUTOPSY CHECKLIST

RECOVER

Researching COVID to Enhance Recovery

RECOVER Initiative Autopsy Checklist for Packaging & Shipping

Checklist does not need to be returned to the PBC

Important Reminders

- ☐ **REDCap data must be in a Complete status and saved prior to handing off the temperature specific shippers to the carrier.**
- ☐ Ensure fields for tissue designations #19, #27, #34, and #52 - #55 (if applicable), are completed and saved in REDCap and on the shipping manifests.
- ☐ Ensure all required fields (*) are completed on the Requisition Form and the manifests are completed accordingly.
- ☐ **Make at least 4 copies of the Requisition Form.** One is needed for each temperature specific return shipper, and the brain specimen (if applicable). The site should retain a copy for their records as well.
- ☐ Ensure the decedent's FFPE blocks and the Cryosettes™ are labeled with the KIT-ID.
- ☐ When packaging the FFPE blocks and the Cryosette™, package them in sequential order into the FFPE Block Transport Containers and Cryosette™ 21-count racks, respectively. Ensure Cryosette™ racks and 96-well matrix storage box are labeled with the decedent's RECOVER Subject ID.
- ☐ If you are dropping off the package at FedEx, confirm with shipping authorities that the FedEx pick up location accepts UN3373 Biological Substances, Category B. This would not apply if using a scheduled FedEx pick-up.
- ☐ Both temperature shippers should only be shipped **Monday – Wednesday** for arrival the PBC **Tuesday – Thursday.**
- ☐ In the 96-count matrix tube storage box, ensure the serum aliquots are in Row A; and the CSF aliquots are in Row C. Print 2 copies of the REDCap Biospecimen Form PDF – one copy per manila envelope to include in refrigerate and frozen shippers.
- ☐ The frozen return shipper should contain (**for one decedent only**):
  - Cryosettes™ in the labeled Cryosette™ 21-count racks
  - Serum aliquots in the 96-well Matrix storage box
  - CSF aliquots in the 96-well Matrix storage box
  - Stool specimen
  - Bronchial swab in the transport media
  - **Requisition Form & REDCap Biospecimen Form PDF in a manila envelope on top of Styrofoam lid, underneath the cardboard flap.**
- ☐ The refrigerate return shipper should contain (**for one decedent only**):
  - FFPE blocks in the Block Transport Containers
  - Labeled Blood spot card
  - **Requisition Form & REDCap Biospecimen Form PDF in a manila envelope on top of Styrofoam lid, underneath the cardboard flap.**
- ☐ **Send an email to the RECOVERPBC@mayo.edu inbox with the Subject ID(s) and respective FedEx tracking numbers of the frozen and refrigerate shippers on the day the case is sent to PBC.**

RETURN TO TABLE OF CONTENTS

F. MASTER TROUBLESHOOTING GUIDE

Blood Collection/Processing		
**Problem**	**Cause**	**Solution**
Unable to centrifuge SST or CPT tubes within desired window	---	1. Please annotate the actual centrifuge date and time.<br>2. Record the procedural deviation in the Kit Annotation field in REDCap.<br>3. Send samples to the PBC
Failed centrifugation on SST or CPT tube (red blood cell contamination or gel layer did not migrate properly)	---	1. Discard specimen and annotate in the Kit Annotation field in REDCap.<br>2. Leave the Collected radio button selected in REDCap.
I did not use all of my allotted aliquot tubes for a specific specimen type.	---	1. Discard any unused aliquots tubes. They cannot be used for another participant.<br>2. Do not send empty aliquot tubes to the PBC.
Matrix or aliquot tube issue – broken tube or cracked tubes, etc.	---	1. Do not send any broken, cracked or defective matrix aliquot tubes containing samples to the PBC.<br>2. Annotate in REDCap the problem with the associated matrix tube ID.<br>3. Discard the defective tube per site's disposal practices.
Kit was used for a different participant than it was registered to in REDCap	---	1. Submit a RECOVER Help Desk Support ticket and choose topic category Biospecimen.<br>2. In the ticket description, include:<br>a. KIT-ID used for the collection.<br>b. Subject ID of the participant the kit was registered to in REDCap.<br>c. Subject ID of the participant the biospecimens were collected on.

		d. Print and include a PDF version of the biospecimen form for this subject. Exact PDF to include in the image below:<br>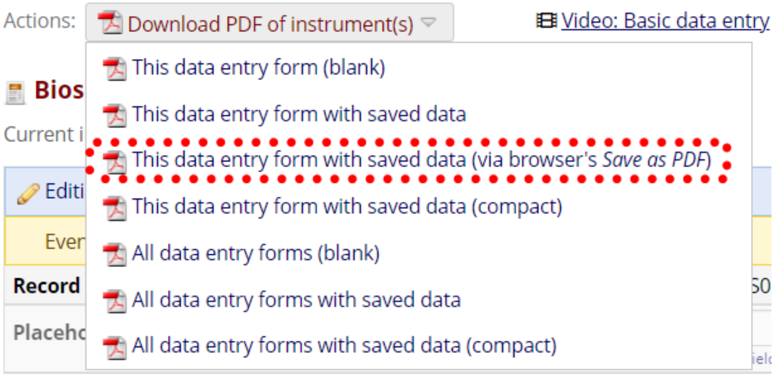<br><br>3. Maintain the collected biospecimens at proper storage temperature pending response from the PBC.
**Shipping**		
**Problem**	**Cause**	**Solution**
When preparing the batch shipping manifest, we do not have a copy of the kit requisition form for a subject.	No copies were made at time of collection and DOC shipping.	1. Print and include a PDF version of the biospecimen form for this subject. Exact PDF to include in the image below:<br>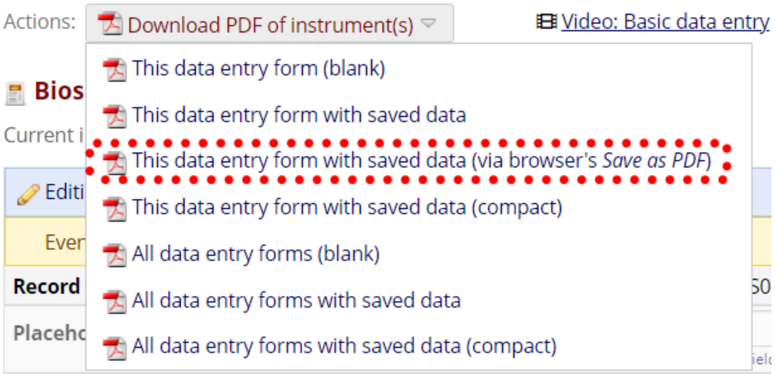
Cannot determine the Subject and KIT-ID for aliquot tubes while preparing the batch shipping manifest.	---	1. Submit a RECOVER Help Desk Support ticket and choose topic category REDCap.<br>2. Provide information in description so DRC can provide corresponding information about the REDCap form.<br>3. Ensure the Biospecimen form is accurate.<br>4. Based on DRC response:<br>a. If subject information is found, samples can be processed. Enter as appropriate into manifest. Verify in REDCap using the information provided by the DRC.<br>b. If subject information NOT found to connect to subject, dispose samples.<br>c. **DO NOT** send aliquots with no linked subject information to the PBC.
Missed last FedEx pick-up time of the day	Late day participant collection or FedEx did not pick up on DOC	Standard Workflow solution:<br>1. If biospecimen collection has already occurred, the collection site should stabilize the specimens at the shipping temperature per the MOP until the next available pick-up time.<br>2. If samples have not been stored at the correct temperature for 48 hours or longer or shipper has been at room temperature for 48 hours or longer:

		a. Submit a RECOVER Help Desk Support ticket and choose topic category Biospecimen.<br>b. Provide information in description.<br>c. Annotate in REDCap<br>d. Ship to the PBC
Missed our batch shipping week because of dry ice availability	Shipping delay	1. If local storage allows, hold batch shipment until the next scheduled batch shipping week for the following month.<br>If local storage space is not sufficient to hold samples, then ship the following week in the Monday-Wednesday batch shipping timeframe.
**IT Outage**		
**Problem**	**Cause**	**Solution**
Unable to access REDCap	Unplanned system outage	1. Submit an Ivanti Help Desk ticket and choose REDCap topic category REDCap.<br>https://nyulangone-recover-amc.ivanticloud.com/Modules/SelfService/?AnonymousRequest=yes#serviceCatalog<br>2. The NIH RECOVER staff will make the final decision as to whether sample collection should be continued or discontinued until the event has cleared.
IT outage occurs during visit but *before* to assignment of Subject ID in REDCap	Unplanned system outage	1. Reschedule collection visit.<br>2. Submit a RECOVER Help Desk Support ticket and choose REDCap topic category
IT outage occurs during visit but *after* linkage of Subject ID in REDCap	Unplanned system outage	1. Record Subject ID information on requisition form and one REDCap downtime form.<br>2. Submit a RECOVER Help Desk Support ticket and choose REDCap topic category<br>3. Once issue is resolved, enter all appropriate information in REDCap.
**Weather Delays**		
**Problem**	**Cause**	**Solution**
Severe weather conditions severely impair or prevent delivery of biospecimens to PBC	---	1. When a severe weather event is expected in impact FedEx operations, the PBC will provide collection guidance via Microsoft Teams.<br>2. If biospecimen collection has already occurred, the collection site is expected to stabilize the specimens until the next available shipping day once shipping is no longer affected by the severe weather.<br>3. The NIH RECOVER staff will make the final decision as to whether sample collection should be continued or discontinued until the event has cleared.

RETURN TO TABLE OF CONTENTS

IV. FORMS

A. F-013.04 PASC Biorepository Core Requisition Form: Autopsy Cohort

PASC Biorepository Core Requisition Form
Research Specimen

MAYO CLINIC

Ext. IRB (RECOVER_PASC_Autopsy) Cicek R3006135

**Study Information:**<br>**RECOVER Autopsy Cohort**<br>**Study ID:** 21-001403<br><br>**Study Contact Information**<br>Please direct all questions to the Study Contact listed below:<br>**Email:** RECOVERPBC@Mayo.edu	**KIT ID:**<br><br>**Subject ID*:** ☐☐☐☐☐☐☐-☐☐☐☐☐
**Study Investigator Information**<br><br>**Investigator:** Mine Cicek, Ph.D.<br>**Email:** Cicek.Mine@Mayo.edu<br><br>**Investigator:** Thomas Flotte, M.D.<br>**Email:** Flotte.Thomas@Mayo.edu<br><br>**Collection Site Contact: (Please print legibly)**<br>**Name:** ____________________<br>**Phone:** ____________________<br>**Email:** ____________________<br>**Collection Notes:**	**Date and Time of Autopsy*** (will serve as specimen collection time)<br>**Date:** ☐☐-☐☐-☐☐☐☐ (Month, Day, Year)<br>**Time:** ☐☐ : ☐☐ (24-hour clock)<br><br>**Hours Since Death***<br>**Hours:** ☐☐

***required information**

FROZEN MATRIX TUBE ALIQUOT MANIFEST (Up to 20 matrix tubes)

1. From the Actions dropdown at the top of the REDCap form, select the "This data entry form with saved data (via browser's Save as PDF)" to print the completed form. See Section 4.1 of the Manual of Procedures (MOP) for image.

2. Print completed REDCap Biospecimen PDF file. The printed PDF should resemble the REDCap screen and will function as a manifest for the matrix tubes, and as a QC check for the FFPE and Cryosette manifests.

3. Enclose printed PDFs with completed Requisition Forms when packaging and shipping the RECOVER 20lb Frozen Autopsy Shipper and the RECOVER 10lb Refrigerate Autopsy Shipper.

REFRIDGERATE FFPE BLOCK MANIFEST (Up to 56 FFPE Blocks)

**Tissue Block Designation**	**Tissue Description**	**Present or Absent** (Please check appropriate box)
01	Sural nerve [up to 3 mm, **Embed on End**]	☐ Present ☐ Absent
02	Peroneal nerve [up to 3mm **Embed on End**]	☐ Present ☐ Absent
03	Skin, Calf including subcutis, 5 mm punch	☐ Present ☐ Absent
04	Muscle, Gastrocnemius	☐ Present ☐ Absent
05	Muscle, Psoas	☐ Present ☐ Absent
06	Duodenum	☐ Present ☐ Absent
07	Ileum	☐ Present ☐ Absent
08	Colon, right	☐ Present ☐ Absent
09	Colon, descending/sigmoid	☐ Present ☐ Absent
10	Adrenal	☐ Present ☐ Absent
11	Aorta, Ascending	☐ Present ☐ Absent
12	Right Ventricle, Posterior	☐ Present ☐ Absent
13	Left Ventricle, Lateral	☐ Present ☐ Absent
14	Left Ventricle, Basal	☐ Present ☐ Absent
15	Right Atrium/SVC (near SA node)	☐ Present ☐ Absent
16	Left Atrium	☐ Present ☐ Absent
17	Coronary artery, right	☐ Present ☐ Absent
18	Coronary artery, left	☐ Present ☐ Absent
19	Cardiac Lesional tissue [**describe**]	☐ Present ☐ Absent
20	Hilar/Mediastinal Lymph nodes	☐ Present ☐ Absent
21	Trachea	☐ Present ☐ Absent
22	Right upper lobe, with pleura	☐ Present ☐ Absent
23	Right middle lobe	☐ Present ☐ Absent
24	Right lower lobe	☐ Present ☐ Absent
25	Left upper lobe, with pleura	☐ Present ☐ Absent
26	Left lower lobe	☐ Present ☐ Absent
27	Lung Lesional Tissue [**describe**]	☐ Present ☐ Absent
28	Spleen	☐ Present ☐ Absent
29	Bone Marrow (rib squeeze)	☐ Present ☐ Absent
30	Pancreas	☐ Present ☐ Absent

31	Liver, right lobe (central)	☐ Present ☐ Absent
32	Mesenteric fat	☐ Present ☐ Absent
33	Kidney, right or left	☐ Present ☐ Absent
34	Renal Lesional tissue [**describe**]	☐ Present ☐ Absent
35	Testis	☐ Present ☐ Absent ☐ N/A
36	Ovary	☐ Present ☐ Absent ☐ N/A
37	Fallopian Tube	☐ Present ☐ Absent ☐ N/A
38	Olfactory bulb	☐ Present ☐ Absent
39	Frontal cortex with leptomeninges	☐ Present ☐ Absent
40	Basal ganglia	☐ Present ☐ Absent
41	Thalamus	☐ Present ☐ Absent
42	Hippocampus	☐ Present ☐ Absent
43	Occipital cortex	☐ Present ☐ Absent
44	Amygdala	☐ Present ☐ Absent
45	Pons	☐ Present ☐ Absent
46	Choroid plexus	☐ Present ☐ Absent
47	Medulla (area postrema)	☐ Present ☐ Absent
48	Cerebellum with dentate	☐ Present ☐ Absent
49	Dura with sinus	☐ Present ☐ Absent
50	Spinal cord (thorax)	☐ Present ☐ Absent
51	Sympathetic chain with ganglia	☐ Present ☐ Absent
56	Dorsal root ganglia	☐ Present ☐ Absent
**Additional Tissue** – Enter tissue source/block description. *Any transplanted tissue should be noted in these additional sections, not using the designated numbers or checkboxes listed above in this block summary.		
52		☐ Present ☐ Absent ☐ N/A
53		☐ Present ☐ Absent ☐ N/A
54		☐ Present ☐ Absent ☐ N/A
55		☐ Present ☐ Absent ☐ N/A

FROZEN CRYOSETTE™ BLOCK MANIFEST (Up to 112 Cryosettes™)

**Tissue Block Designation** (in duplicate)	**Tissue Description**	**Present or Absent** (Please check appropriate box)	**Tissue Not Sufficient** (check if not enough tissue for one or both Cryosettes™)
01, 01	Sural nerve [up to 3 mm, **Embed on End**]	☐ Present ☐ Absent	☐
02, 02	Peroneal nerve [up to 3 mm **Embed on End**]	☐ Present ☐ Absent	☐
03, 03	Skin, Calf including subcutis, 5 mm punch	☐ Present ☐ Absent	☐
04, 04	Muscle, Gastrocnemius	☐ Present ☐ Absent	☐
05, 05	Muscle, Psoas	☐ Present ☐ Absent	☐
06, 06	Duodenum	☐ Present ☐ Absent	☐
07, 07	Ileum	☐ Present ☐ Absent	☐
08, 08	Colon, right	☐ Present ☐ Absent	☐

09, 09	Colon, descending/sigmoid	☐ Present ☐ Absent	☐
10, 10	Adrenal	☐ Present ☐ Absent	☐
11, 11	Aorta, Ascending	☐ Present ☐ Absent	☐
12, 12	Right Ventricle, Posterior	☐ Present ☐ Absent	☐
13, 13	Left Ventricle, Lateral	☐ Present ☐ Absent	☐
14, 14	Left Ventricle, Basal	☐ Present ☐ Absent	☐
15, 15	Right Atrium/SVC (near SA node)	☐ Present ☐ Absent	☐
16, 16	Left Atrium	☐ Present ☐ Absent	☐
17, 17	Coronary artery, right	☐ Present ☐ Absent	☐
18, 18	Coronary artery, left	☐ Present ☐ Absent	☐
19, 19	Cardiac Lesional tissue [**describe**]	☐ Present ☐ Absent<br>☐ Same as paraffin	☐
20, 20	Hilar/Mediastinal Lymph nodes	☐ Present ☐ Absent	☐
21, 21	Trachea	☐ Present ☐ Absent	☐
22, 22	Right upper lobe, with pleura	☐ Present ☐ Absent	☐
23, 23	Right middle lobe	☐ Present ☐ Absent	☐
24, 24	Right lower lobe	☐ Present ☐ Absent	☐
25, 25	Left upper lobe, with pleura	☐ Present ☐ Absent	☐
26, 26	Left lower lobe	☐ Present ☐ Absent	☐
27, 27	Lung Lesional Tissue [**describe**]	☐ Present ☐ Absent<br>☐ Same as paraffin	☐
28, 28	Spleen	☐ Present ☐ Absent	☐
29, 29	Bone Marrow (rib squeeze)	☐ Present ☐ Absent	☐
30, 30	Pancreas	☐ Present ☐ Absent	☐
31, 31	Liver, right lobe (central)	☐ Present ☐ Absent	☐
32, 32	Mesenteric fat	☐ Present ☐ Absent	☐
33, 33	Kidney, right or left	☐ Present ☐ Absent	☐
34, 34	Renal Lesional tissue [**describe**]	☐ Present ☐ Absent<br>☐ Same as paraffin	☐
35. 35	Testis	☐ Present ☐ Absent ☐ N/A	☐
36, 36	Ovary	☐ Present ☐ Absent ☐ N/A	☐
37, 37	Fallopian Tube	☐ Present ☐ Absent ☐ N/A	☐
38, 38	Olfactory bulb	☐ Present ☐ Absent	☐
39, 39	Frontal cortex with leptomeninges	☐ Present ☐ Absent	☐
40, 40	Basal ganglia	☐ Present ☐ Absent	☐
41, 41	Thalamus	☐ Present ☐ Absent	☐
42, 42	Hippocampus	☐ Present ☐ Absent	☐
43, 43	Occipital cortex	☐ Present ☐ Absent	☐
44, 44	Amygdala	☐ Present ☐ Absent	☐
45, 45	Pons	☐ Present ☐ Absent	☐
46, 46	Choroid plexus	☐ Present ☐ Absent	☐
47, 47	Medulla (area postrema)	☐ Present ☐ Absent	☐
48, 48	Cerebellum with dentate	☐ Present ☐ Absent	☐
49, 49	Dura with sinus	☐ Present ☐ Absent	☐
50, 50	Spinal cord (thorax)	☐ Present ☐ Absent	☐
51, 51	Sympathetic chain with ganglia	☐ Present ☐ Absent	☐

56, 56	Dorsal root ganglia	☐ Present ☐ Absent	☐
**Additional Tissue** – Enter tissue source/block description. *Any transplanted tissue should be noted in these additional sections, not using the designated numbers or checkboxes listed above in this block summary.			
52, 52		☐ Present ☐ Absent ☐ N/A ☐ Same as paraffin	
53, 53		☐ Present ☐ Absent ☐ N/A ☐ Same as paraffin	
54, 54		☐ Present ☐ Absent ☐ N/A ☐ Same as paraffin	
55, 55		☐ Present ☐ Absent ☐ N/A ☐ Same as paraffin	

RETURN TO TABLE OF CONTENTS

V. REVISION/DOCUMENT HISTORY

Effective Date	Version	Synopsis of Change
12/8/2021	1.0	Document created and assigned SOP-047.
04/14/2022	2.0	**Major Change:** Added specific procedure for snap freezing. Added equipment for snap freezing to the Equipment Guide. Added step in frozen shipping section to cover specimens and fill Styrofoam completely with dry ice. Edited shipping days to be only Monday – Wednesday. Updated shipping temperature for 10 lb RECOVER Autopsy Shipper from ambient to refrigerate throughout.<br><br>**Minor Change:** Removed individual SOP numbers and reassigned numbers to forms, template, key, and checklist. Replaced requisition form with updated version (replace Mayo Clinic Bio Services log with Mayo Clinic logo). Removed mention of Imitative to align with Program language. Replaced updated CL-002.02. Removed steps for affixing Waybill. Added Profile Management steps to Ordering RECOVER PBC Kits section. Updated product description and item number for blood spot card.
04/22/2022	2.1	**Minor Change:** Added options for snap freezing using liquid nitrogen. Added fixation guidelines. Added note to send FedEx tracking numbers to PBC on day of shipment.
01/16/2023	3.0	**Major Change:** Added dorsal root ganglia source to designated FFPE and snap frozen tissue collection – spot #56. Edited additional tissue block numbers to 52-55. Updated and added Ivanti Help Desk hyperlink throughout MOP. Updated 96-well matrix storage box images for matrix tube storage. Added SST tube fixed-angle centrifuge processing specifications. Updated refrigerate and frozen shipper images. Added writing Subject ID on 96-well matrix tube storage box and Cryosette™ 21-count Racks. Added enclosing REDCap Biospecimen PDF and the completed requisition form in a manila envelope for all return shippers. Highlighted in yellow all MOP updates and edits. Fixed hyperlinks. Removed hardcopy recording of date and time of fixation, SST centrifuge processing date and time, and serum and CSF freeze date and time data on the PASC Biorepository Core Requisition Form. Added reminder to not send empty biospecimen collection containers to the PBC.<br>**Minor Change:** Minor formatting changes made throughout document.
2/27/2023	3.1	**Minor change:** KIT-IDs are required on all cassettes and cryosettes containers. Sites will hand label current kits in their inventory. PBC will add KIT-IDs to all cassettes and cryosettes for all kit orders starting immediately. Sites should include either the REDCap biospecimen PDF with all biospecimen collections or the completed requisition FFPE and Cryosette block manifests with each shipper. The first page of the requisition form must always be included with shipment. Added page numbers to MOP. Updated Troubleshooting guide. Highlighted in yellow all MOP updates and edits. Updated F-013.04 with enlarged text boxes and removed extra box in date, month portion.

VI. APPROVALS

Nicole Zahnle Digitally signed by Nicole Zahnle
Date: 2023.02.27 11:11:02 -06'00'

AUTHOR – PBC EDUCATION SPECIALIST

Samantha Wirkus Digitally signed by Samantha Wirkus
Date: 2023.02.24 16:38:01 -06'00'

PBC PROGRAM MANAGER

Mine Cicek, Ph.D. Digitally signed by Mine Cicek, Ph.D.
Date: 2023.02.25 15:32:20 -06'00'

PBC CO-PRINCIPAL INVESTIGATOR

Thomas Flotte, M.D. Digitally signed by Thomas Flotte, M.D.
Date: 2023.02.27 10:35:57 -06'00'

PBC CO-PRINCIPAL INVESTIGATOR
